# The every woman study™ low- and middle-income countries edition protocol: A multi-country observational study to assess opportunities and challenges to improving survival and quality of life for women with ovarian cancer

**DOI:** 10.1371/journal.pone.0298154

**Published:** 2024-05-29

**Authors:** Frances Reid, Tracey Adams, Rafe Sadnan Adel, Carlos E. Andrade, Anmol Bajwa, Ian G. Bambury, Nada Benhima, Raikhan Bolatbekova, David Cantu-De Leon, Phaedra Charlton, Carlos Chávez Chirinos, Robin Cohen, Mary Eiken, Erick Estuardo Estrada, Dilyara Kaidarova, Iren Lau, Clara MacKay, Precious Takondwa Makondi, Asima Mukhopadhyay, Aisha Mustapha, Florencia Noll, Martin Origa, Jitendra Pariyar, Shahana Pervin, Ngoc T. H. Phan, Basel Refky, Afrin F. Shaffi, Eva-Maria Strömsholm, Yin Ling Woo, Sook-Yee Yoon, Nargiza Zakirova, Runcie C. W. Chidebe, Garth Funston, Isabelle Soerjomataram

**Affiliations:** 1 World Ovarian Cancer Coalition, Toronto, Ontario, Canada; 2 Department of Obstetrics and Gynaecology, Groote Schuur Hospital, Cape Town, South Africa; 3 South African Medical Research Centre Gynaecological Cancer Research Centre (SA MRC UCT GCRC), Cape Town, South Africa; 4 CancerBD, Dhaka, Bangladesh; 5 Department of Gynecologic Oncology, Barretos Cancer Hospital, Barretos, Brazil; 6 University of the West Indies, Kingston, Jamaica; 7 Department of Medical Oncology, Mohammed VI University Hospital, Marrakech, Morocco; 8 Kazakh Institute of Oncology and Radiology, Almaty, Kazakhstan; 9 Instituto Nacional de Cancerología, Ciudad de México, México; 10 Instituto Regional de Enfermedades Neoplásicas, Arequipa, Perú; 11 Sandy Rollman Ovarian Cancer Foundation, Wynnewood, Pennsylvania, United States of America; 12 International Gynecologic Cancer Society, Chicago, Illinois, United States of America; 13 Hospital General San Juan de Dios, Guatemala City, Guatemala; 14 Ovarian Cancer Malaysia, Kuala Lumpur, Malaysia; 15 National Cancer Center, Kamuzu Central Hospital, Kamuzu, Malawi; 16 Kolkata Gynaecological Oncology Trials and Translational Research Group, Kolkata, India; 17 Ahmadu Bello University Teaching Hospital, Zaria, Kaduna State, Nigeria; 18 Sanatorio Allende, Cordoba, Argentina; 19 Uganda Cancer Institute, Kampala, Uganda; 20 Civil Service Hospital of Nepal, Kathmandu, Nepal; 21 National Institute of Cancer Research & Hospital, Dhaka, Bangladesh; 22 Danang Oncology Hospital, Danang, Vietnam; 23 Mansoura University Oncology Center, Mansoura, Egypt; 24 Moi University/Moi Teaching and Referral Hospital, Eldoret, Kenya; 25 Gynecological Cancer Patients in Finland, Helsinki, Finland; 26 Department of Obstetrics and Gynaecology, University Malaya Medical Centre, Kuala Lumpur, Malaysia; 27 Republican Specialized Scientific and Practical Medical Center of Oncology and Radiology of the Ministry of Health of the Republic of Uzbekistan, Tashkent, Uzbekistan; 28 Project PINK BLUE, Abuja, Nigeria; 29 Department of Sociology & Gerontology, Miami University, Oxford, Ohio, United States of America; 30 Wolfson Institute of Population Health, Queen Mary University of London, London, United Kingdom; 31 Cancer Surveillance Branch, International Agency for Research on Cancer, Lyon, France; South African Medical Research Council, SOUTH AFRICA

## Abstract

**Background:**

Ovarian cancer is a challenging disease to diagnose and treat effectively with five-year survival rates below 50%. Previous patient experience research in high-income countries highlighted common challenges and opportunities to improve survival and quality of life for women affected by ovarian cancer. However, no comparable data exist for low-and middle-income countries, where 70% of women with the disease live. This study aims to address this evidence gap.

**Methods:**

This is an observational multi-country study set in low- and middle-income countries. We aim to recruit over 2000 women diagnosed with ovarian cancer across multiple hospitals in 24 countries in Asia, Africa and South America. Country sample sizes have been calculated (n = 70–96 participants /country), taking account of varying national five-year disease prevalence rates. Women within five years of their diagnosis, who are in contact with participating hospitals, are invited to take part in the study. A questionnaire has been adapted from a tool previously used in high-income countries. It comprises 57 multiple choice and two open-ended questions designed to collect information on demographics, women’s knowledge of ovarian cancer, route to diagnosis, access to treatments, surgery and genetic testing, support needs, the impact of the disease on women and their families, and their priorities for action. The questionnaire has been designed in English, translated into local languages and tested according to local ethics requirements. Questionnaires will be administered by a trained member of the clinical team.

**Conclusion:**

This study will inform further research, advocacy, and action in low- and middle-income countries based on tailored approaches to the national, regional and global challenges and opportunities. In addition, participating countries can choose to repeat the study to track progress and the protocol can be adapted for other countries and other diseases.

## Introduction

### Global incidence and mortality

Ovarian cancer is the eighth most common cancer and eighth most common cause of cancer death in women worldwide [1]. GLOBOCAN estimates that in 2020 there were approximately 314,000 cases diagnosed, 207,000 deaths, and more than 823,000 women living within five years of their diagnosis. Women in low- and middle-income countries (LMICs) account for 70% of ovarian cancer cases [2]. A 42% increase in ovarian cancer incidence, and a 52% increase in mortality, has been predicted by 2040, with the greatest increase expected in LMICs driven by projected population increases [3, 4]. For example, Zambia is predicted to have an increase of 122% in ovarian cancer incidence and 132% in mortality. Further increases are expected linked to the changing prevalence of cancer risk factors, such as lack of physical activity, changing diets, additional body weight, and increasing urbanisation [5–8].

### Challenges associated with ovarian cancer

Five-year survival for ovarian cancer is below 50% even in high-income countries (HICs), and there has been little progress in improving survival in recent years [9]. Nearly two-thirds of women are diagnosed with advanced disease (FIGO stage III or IV) [10]. A major screening trial using ultrasound and serial measurements of Ca 125 did not demonstrate a mortality benefit and most women are diagnosed after they develop symptoms [11]. Ovarian cancer is a difficult disease to diagnose, and patient and doctor delays are common, in part due to the non-specific nature of the symptoms [12].

At present there is little data from LMICs on the burden of ovarian cancer, including information on types of cancer and stage at diagnosis, pathways to diagnoses, clinical outcomes and the perceptions and experiences of women and their families. Even basic information in incidence is lacking. The proportion of the world’s total population covered by cancer registries included in Cancer Incidence in 5 continents volume XI is just 15%, varying by continent with just 1% of Africa, 7% of Asia and 8% of South America [13]. In addition, many LMICs have prioritised certain cancers in women such as breast and cervical cancer where screening could have a major impact on mortality [14, 15]. The lack of a clear understanding of the burden of ovarian cancer in LMICs and the subsequent lack of focus could hamper the development of approaches aimed at improving ovarian cancer outcomes.

### Known opportunities for progress

Despite these challenges, there are opportunities for short term progress to improve survival, morbidity, and quality of life. Symptom patterns and the nuances of current diagnostic tests are now better understood [16–18], which could contribute to more timely diagnosis. Improved organisation of diagnostic and treatment services and adherence to guidelines show the potential to reduce delays in diagnosis and improve access to treatment, with a likely positive impact on mortality [19–22].

New targeted treatments specifically PARP-inhibitors are emerging for sub-groups of patients. In high-income countries. genetic testing is increasing access to these treatments and improving identification of those most at risk of developing the disease due to their family history [23]. It has been shown that 18% of women with high-grade serous ovarian cancer, the most common and lethal form of the disease, have inherited mutations in BRCA1 or BRCA2 genes [10]. Identifying family members who are at risk, and taking action (e.g. preventative surgery), has the potential to prevent many cases and this approach has already been deemed cost effective in both high- and upper-middle income countries [24]. However, accessibility and affordability of genetic testing and associated targeted treatments for women with ovarian cancer is still very limited in LMICs, thus currently impacting negatively on their outcomes. However this is an opportunity for progress in these settings.

The World Ovarian Cancer Coalition (Coalition) is a partnership organisation of over 200 patient advocacy and support groups from 50 countries with a mission to improve survival and quality of life for women, no matter where they live. In 2018 it undertook The Every Woman Study^TM^, the largest patient experience study to date of women with ovarian cancer. The study included 1531 women from 44 countries, who took part in an in-depth online survey. While the published results showed a common set of challenges, it revealed wide variation between countries on key metrics, highlighting opportunities for improvement [12]. Domains within the study included the route to diagnosis; time to diagnosis; women’s knowledge of the disease pre-diagnosis; family history and genetic testing; access to treatments, surgery and clinical trials; support needs; women’s quality of life; and their priorities for action. However, a key limitation of the study was that 95% of responses came from high-income countries (HICs).

### Aim

In this study, we aim to establish a large patient experience evidence base, and characterise the key challenges and opportunities to improve survival and quality of life for women in LMICs. We will test our hypothesis that key factors vary by country, requiring bespoke potential health strategies. With the evidence obtained from this study we expect to raise the profile of ovarian cancer in LMICs. We hope to enable national teams to engage governments and health policy makers; secure extra funding for diagnostics, research and awareness campaigns; and improve support for women. Examples of this type of approach have been successfully undertaken in high-income countries [25–28].

## Methods

### Design and setting

The study has been developed by the Coalition, in partnership with the International Gynecologic Cancer Society (IGCS). It is overseen by a global oversight committee (OC) comprising clinical and patient advocates from six geographic areas, together with additional data and diagnostic expertise.

This study adopts a low risk, observational, cross-sectional design. A standardised questionnaire, derived from a previously published set of questions used in an online survey with participants recruited via social media in 2018 [12], has been developed to explore the experiences of women with ovarian cancer in LMICs based on symptoms, route to diagnosis, treatment, and quality of life. This study is designed as a one-off interaction with no follow-up, with recruitment of women via participating hospital clinics to maximise uptake.

### Survey development and modification

The 2018 survey questions were originally developed by analysing structured interviews with eight women who had ovarian cancer. The draft survey underwent repeated testing by 23 women with ovarian cancer from 13 countries, including four middle-income countries [12]. In 2021, a panel of 13 clinicians from eight LMICs were questioned regarding the content validity, cultural sensitivity and importance of the topics and questions used in the 2018 study. Subsequent expert input from the OC, which includes six patient representatives, was obtained. This process reduced the original survey from 148 questions to 59; those considered most relevant and important for the LMIC setting. Almost all questions were multiple-choice, but two questions allowed for free-text input. This reduction in length makes data collection within hospital clinics feasible either on paper, online, or by interview, increasing accessibility for those with limited literacy skills or digital access. Face validity of the survey in English was then tested by eight women with ovarian cancer in hospitals in South Africa, Kazakhstan, Vietnam, and Argentina. All found it an acceptable length, appropriate to their setting and experiences, and not missing any key information. Table 1 shows the question domains, and some sample questions. The OC approved the review process and the final version of the survey (S1 File).

**Table 1:** Every woman study^TM^ question domains and examples.

Domain	Example questions	
About You 10 questions	Q.6	Just before you were diagnosed, in your view what was your household income? • Below average for your country • Average for your country • Above average for your country • Prefer not to say
Family History 3 questions	Q.11	Have any of the following family relatives (i.e., blood relatives on either your mother or your father’s side of the family) had ovarian cancer? **TICK ALL THAT APPLY** • Mother • Daughter • Sister • Aunt • Cousin • Grandmother (mother’s side) • Grandmother (father’s side) • Other more distant relatives (mother’s side) • Other more distant relatives (father’s side) • No, none of my close family have been affected
Route to diagnosis 14 questions	Q.21	For women who said they experienced symptoms Which type of person, other than a family member, did you **first** seek advice from, about your symptoms? **SELECT ONLY** **ONE** **ANSWER** • A local healer • An alternative health practitioner • A family doctor • A gynaecologist • A gynaecologic oncologist (a doctor specialising in the treatment of ovarian cancer) • A gastroenterologist • An emergency room or accident and emergency doctor • A nurse • A pharmacist • Someone else
Treatments for ovarian cancer 9 questions	Q.29	In deciding what, if any, treatments you will have to control your ovarian cancer or deal with side effects from treatment, which of the following will affect your decision? **TICK ALL THAT APPLY** • The opinion of the doctor • The opinion of my family • I will make up my own mind • The cost of treatment drugs • Other costs associated with treatment such as transport or accommodation • The chance to cure or extend my life • The side effects of treatment • None of the above
Emotional support needs 7 questions	Q.38	For women who have experienced support needs Are there particular issues you have faced? **TICK ALL THAT APPLY** • Fear of the cancer returning • Fear that treatment will not work • Fear of dying • Difficulty with getting back to ‘normal life’ after treatment • Partner or spouse leaving • Other issues relating to family and friends • Feelings of isolation • Feeling unable to talk to others • Loss of fertility • Regaining sexual intimacy with a partner • Coping with the menopause • Dealing with stigma because of the cancer diagnosis • Other • None in particular
Practical support needs 5 questions	Q.47	Has having a diagnosis of ovarian cancer had an impact on your financial situation? **SELECT ONLY** **ONE** **ANSWER** • Yes to a great extent • Yes to some extent • Not much • Not at all • I would prefer not to say
Information needs 5 questions	Q53	If this hospital were able to provide women with information about living with ovarian cancer, what do you think it should include? **TICK ALL THAT APPLY** • Information about treatments and diagnosis • Information about living with ovarian cancer and what to expect • Information on how to manage physical and mental health • Managing ovarian cancer that can no longer be treated • Sources of local or national support • A way to meet other women with ovarian cancer in person or online • The hospital already supplies the information I need • Other • I would not like them to provide information
Final Questions 6 questions including COVID-19, trials, priorities for action	Q.59	Is there something that is particularly important to you about your experience of ovarian cancer that you would like to share with the study team?

### Study infrastructure

**Fig 1:**
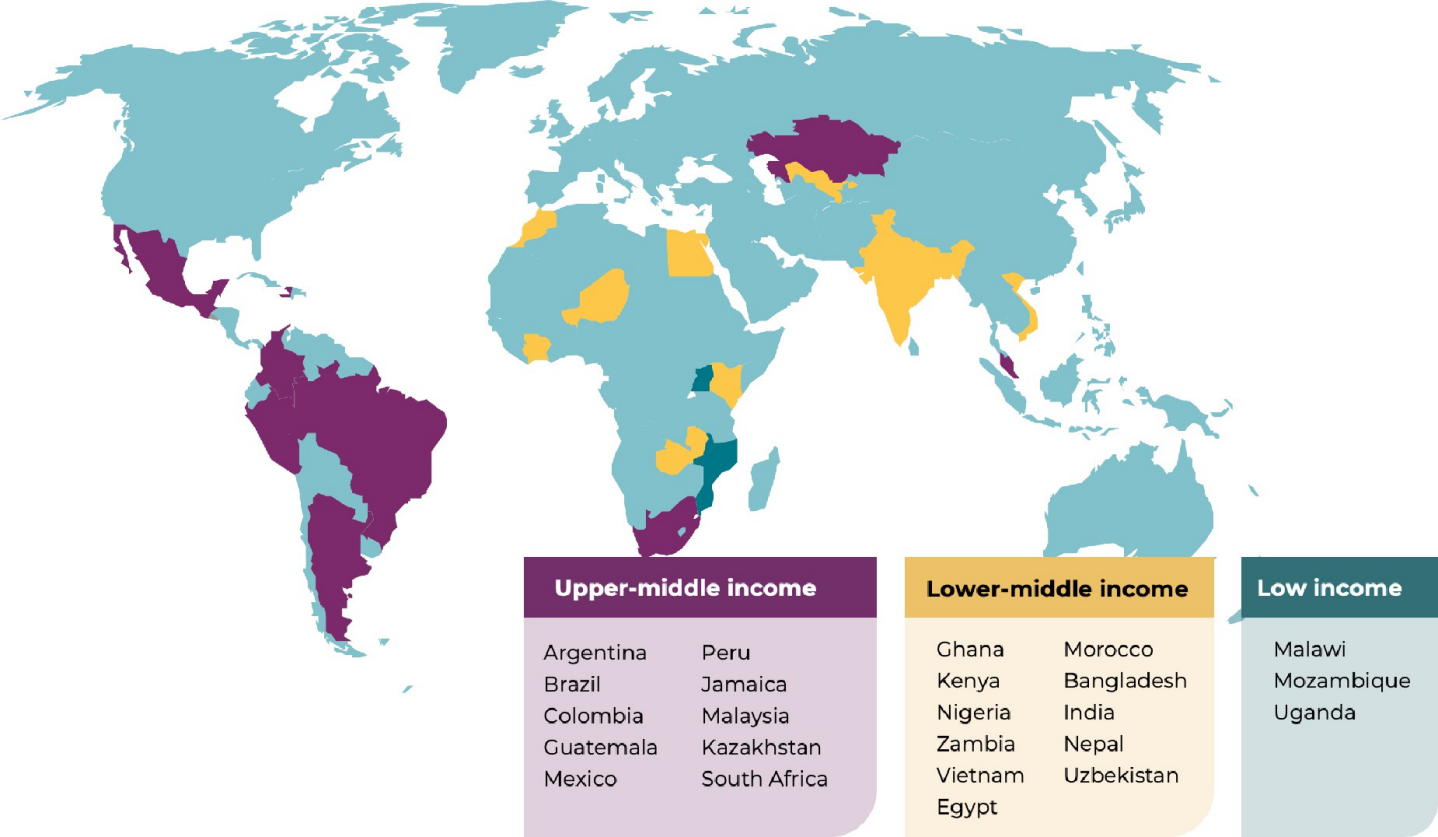
Participating countries by income-level World Bank status June 2021.

To reach sufficient participants and maximise their ability to participate, the OC proposed collaborating with a selection of clinicians treating women with ovarian cancer either known to the Coalition, or through the extensive network of IGCS members. An initial target list of 31 LMICs was drawn up and approaches made to clinicians and national and regional gynecologic oncology societies. In total, 113 clinical sites have been recruited using snowballing methods across 24 countries (Fig 1). Classification of country income level was determined using the World Bank open data, accessed in June 2021 [29].

**Fig 2:**
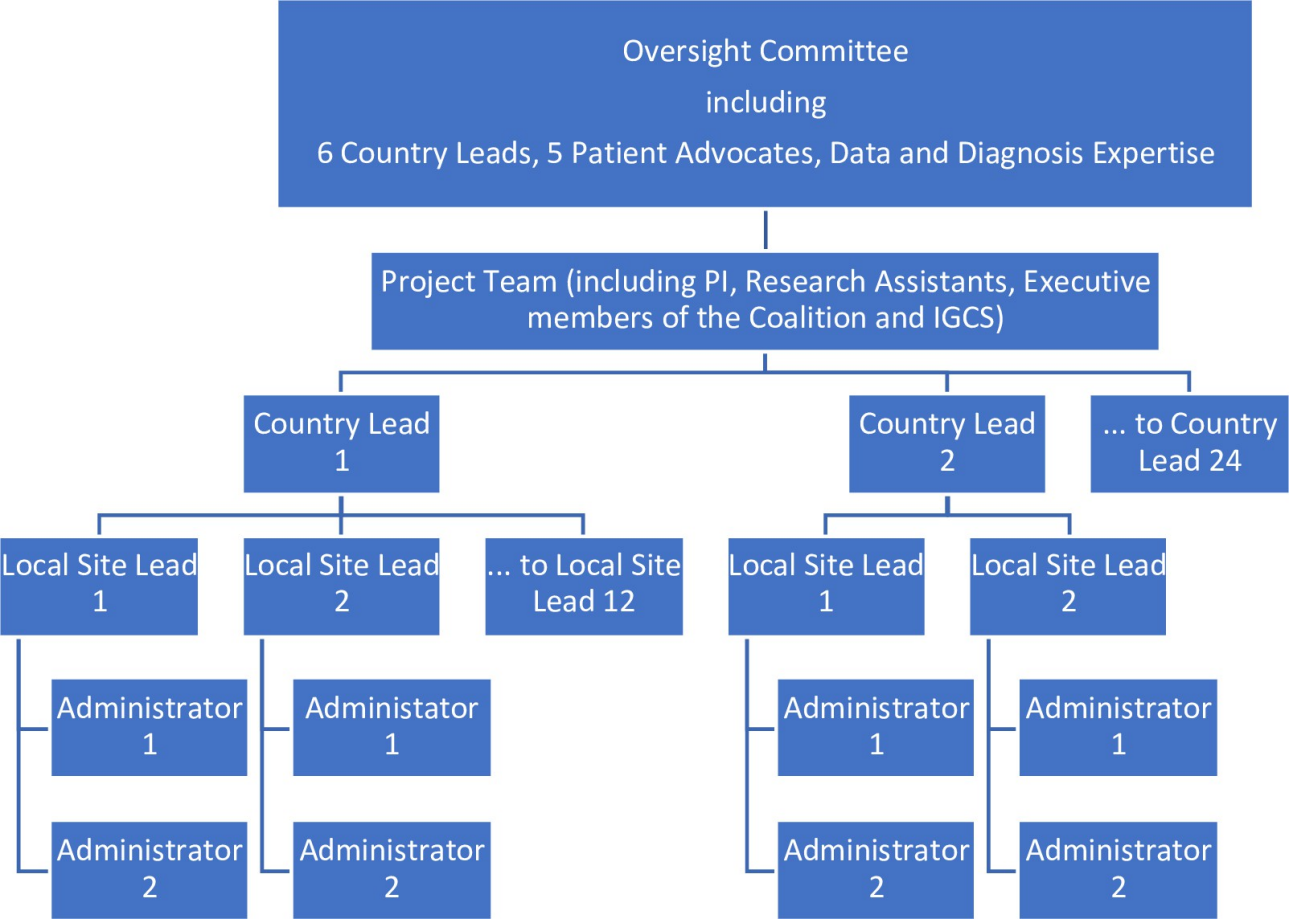
Governance structure of the study.

Within each country, a lead clinician (CLC) was assigned to coordinate the national activity and secure approvals by local or national ethics committees as required. As an example, the protocol submitted in Kenya, is included as a S2 File. CLCs advised on the languages needed and provided contacts for translators who could meet the requirements of the various ethics committees. The survey has been translated into 28 languages using the agencies recommended by the CLCs, and translations checked by back translation where required by national ethics, the clinical team, the study team, and by one or two local patients. The CLC is responsible for the implementation of the protocol, choosing optimal methods of sample selection and data collection for their setting. The CLC is responsible for building their network of participating sites, each with a local site lead (LSL). Depending on the health infrastructure and resources available, countries may have between one and 12 sites, ideally representing the typical range of care women might receive within the country. This is summarised in Fig 2.

As part of the study, each CLC also participates in a qualitative study using semi-structured interviews to explore the care of women with ovarian cancer in their country and the opportunities for progress. They also provide additional information about care in their country collected in survey format (S3 and S4 Files). These elements will be analysed separately and used to help provide context for the global analysis of the Every Woman Study™ LMIC Edition, and content for the country reports.

Each LSL is responsible for identifying research assistants for the study (nurse, researcher, doctor, social worker or similar with experience in speaking with patients with ovarian cancer), to administer the questionnaire and enter data onto the REDCap electronic data capture tools hosted at the International Gynecologic Cancer Society [30, 31]. REDCap (Research Electronic Data Capture) is a secure, web-based software platform designed to support data capture for research studies, providing 1) an intuitive interface for validated data capture; 2) audit trails for tracking data manipulation and export procedures; 3) automated export procedures for seamless data downloads to common statistical packages; and 4) procedures for data integration and interoperability with external sources.

The research assistants are known as an administrator of the study. To keep consistency in terms of data collection, the administrators are kept to a minimum number per site, ideally one or two. It is important for local teams to consider which person or role will be able to communicate most effectively with the women, bearing in mind their situation and who they would feel most comfortable talking to. This can vary between countries and was highlighted during the testing phase. Each CLC, LSL and administrator is provided with access to REDCap. Training on the system is provided by the Project Management team (live sessions, video recordings and/or PowerPoint guides).

### Sample selection

Initially the OC recommended a standardised single approach to sample selection, with all patients diagnosed within the last five years at a participating site being approached for inclusion in the study. This would have allowed for inclusion of survivors with a wide-spread time since diagnosis, and reached patients who might otherwise have stopped treatment or be in remission. However, given the diversity of resources, attendance patterns, and access to patient records between countries and even participating centres within a country, the OC decided a single approach was not feasible. A randomised approach was also not deemed suitable given the challenges ensuring consistency in implementation across many sites. Instead, a convenience sampling approach was approved, offering three different models listed below. To compensate for the variety of approaches, the method of sample selection will be recorded for each patient to allow for data stratification if required. CLCs suggest all participating centres in their country follow the same approach, but this is not mandated. The sample selection approaches, and associated caveats are as follows:

1. The site team reviews all cases on their records that have been seen in the previous five years, with a view to contacting eligible ovarian cancer patients and inviting them to participate. This approach is dependent on availability of staff resource, accessible patient records and reliable communication methods with patients. It might also catch patients who have previously stopped treatment but may also require them to attend a new appointment if they cannot be reached by email, or WhatsApp, and so completion rates may be lower;
2. The site team reviews all cases of ovarian cancer patients who have recently attended an appointment, working backwards in a sequential manner, until they meet the minimum or preferred sample size. This requires extra resource and good record-keeping, and may mean the sample selected is skewed to those with a more recent diagnosis; and
3. The site team reviews all cases of ovarian cancer patients due to attend a forthcoming appointment in person or virtually, inviting eligible patients to participate at that appointment. This approach requires less staff resources, as patient notes and attendance in clinic is already factored in. It may also improve completion rates. However, this approach may result in the inclusion of a greater proportion of patients with a more recent diagnosis, those on active treatment or experiencing complications, and can add time to clinic appointments.

### Sample size

The minimum sample size for each country is based on GLOBOCAN estimates of five-year disease prevalence in the country, producing results at the 95% confidence level (+/- 10%) [2]. CLCs can choose to recruit more patients to improve confidence intervals in their standalone results. In total, the study aims to collect data from at least 1534 women, which would provide results at a 95% confidence level, +/- 2.5%, based on global five-year prevalence. Should each of the 24 countries (3 low, 11 lower-middle and 10 upper-middle income countries) collect their minimum data, the sample size would be 2,160 (Table 2).

**Table 2:** Expected sample sizes by country and income level.

REGIONAL AREA	COUNTRY	WORLD BANK INCOME STATUS (JUNE 2021) [29]	MINIMUM SAMPLE SIZE	PREFERRED SAMPLE SIZE	FIVE-YEAR PREVALENCE OF OVARIAN CANCER
**Sub-Saharan Africa**	Ghana	Lower-middle	92	327	2190
	Kenya	Lower-middle	92	330	2314
	Malawi	Low	79	203	431
	Mozambique	Low	70	155	259
	Nigeria	Lower-middle	95	361	6079
	South Africa	Lower-middle	94	347	3559
	Uganda	Low	89	297	1242
	Zambia	Lower-middle	78	197	403
**North Africa**	Egypt	Lower-middle	95	364	6854
	Morocco	Lower-middle	93	340	395
**South-East Asia**	Malaysia	Upper-middle	94	357	4989
	Vietnam	Lower-middle	95	346	3468
**Western Asia**	Kazakhstan	Lower-middle	93	343	3207
	Uzbekistan	Lower-middle	92	322	1997
**Caribbean, Central, South America**	Argentina	Upper-middle	95	362	6192
	Brazil	Upper-middle	96	377	18912
	Colombia	Upper-middle	95	362	6344
	Guatemala	Upper-middle	83	236	604
	Jamaica	Upper-middle	72	165	290
	Mexico	Upper-middle	95	374	13529
	Peru	Upper-middle	93	345	3398
**South-central Asia**	Bangladesh	Lower-middle	95	364	7044
	India	Lower-middle	96	383	103716
	Nepal	Lower-middle	89	297	1297
Total		Lower-middle	2160	6815	198,713

### Inclusion and exclusion criteria

The inclusion criteria for the study are as follows:

1. The woman with ovarian cancer is a patient at one of the participating hospitals;
2. The woman with ovarian cancer is willing and able to give informed consent for participation in the study;
3. The woman is biologically female, aged between 18 and 99 years at the time of recruitment;
4. The woman has been diagnosed with any stage or type (including borderline) of primary ovarian, fallopian tube or primary peritoneal cancer within the previous five years (of the date of completing the survey); and
5. The woman has already been informed of their diagnosis of ovarian cancer at a previous appointment and the administrator is confident the woman with ovarian cancer understands what they have been told. If there is any doubt, the administrator must check with the clinician caring for the patient.

The participant is excluded from the study if any of the following apply:

1. The woman is attending the hospital or clinic to receive their diagnosis of ovarian cancer;
2. The woman is deemed too unwell to be able to cope with the demands of filling in the survey or responding to questions;
3. The woman is identified as being unable to cope with the demands of filling in the survey or responding to questions, or does not understand her diagnosis as a result of learning difficulties, medical conditions or mental health concerns; and
4. The woman has already completed the survey at a previous visit to the hospital.

### Data collection procedure

The administrator generates a unique identifier number (UIN) for the ovarian cancer patients on REDCap and some basic demographic and clinical data is entered to determine eligibility (S6 File). This information includes age at diagnosis, month and year of diagnosis, type and stage of ovarian cancer and current treatment status. This provides an overview of women attending the clinics and allows basic comparisons between the groups who do and don’t consent.

Women with ovarian cancer are then approached and taken through an informed consent process. They are given written and verbal information about the study. If they are not attending clinics directly, they can be sent this information by email or be spoken to by phone. They are taken through the purpose and outline of the study and the risks and benefits of taking part are discussed. They are informed that they do not have to participate, they do not have to answer every question, and that they can withdraw their information up to 3 months after the close of data collection. They are then asked whether or not they consent to participate in the study and if they consent, whether they wish to i) self-complete a paper survey, ii) self-complete the survey via a secure email link, iii) self-complete the survey via a secure WhatsApp link (or other online messaging app), or iv) whether they wish to have the administrator ask them the questions. Their decision is recorded. Women who receive the information in person, or by phone need to record their consent in writing, and a copy is kept in a secure location on site. In addition to the standard consent form for the study, some local sites require patients to also complete their own informed consent forms, as part of the ethics approval. One country asked for women to consent with a thumbprint. Women who receive the information by email or WhatsApp can consent electronically at the start of the survey, or they can request an administrator contact them if they have questions before deciding.

**Fig 3:**
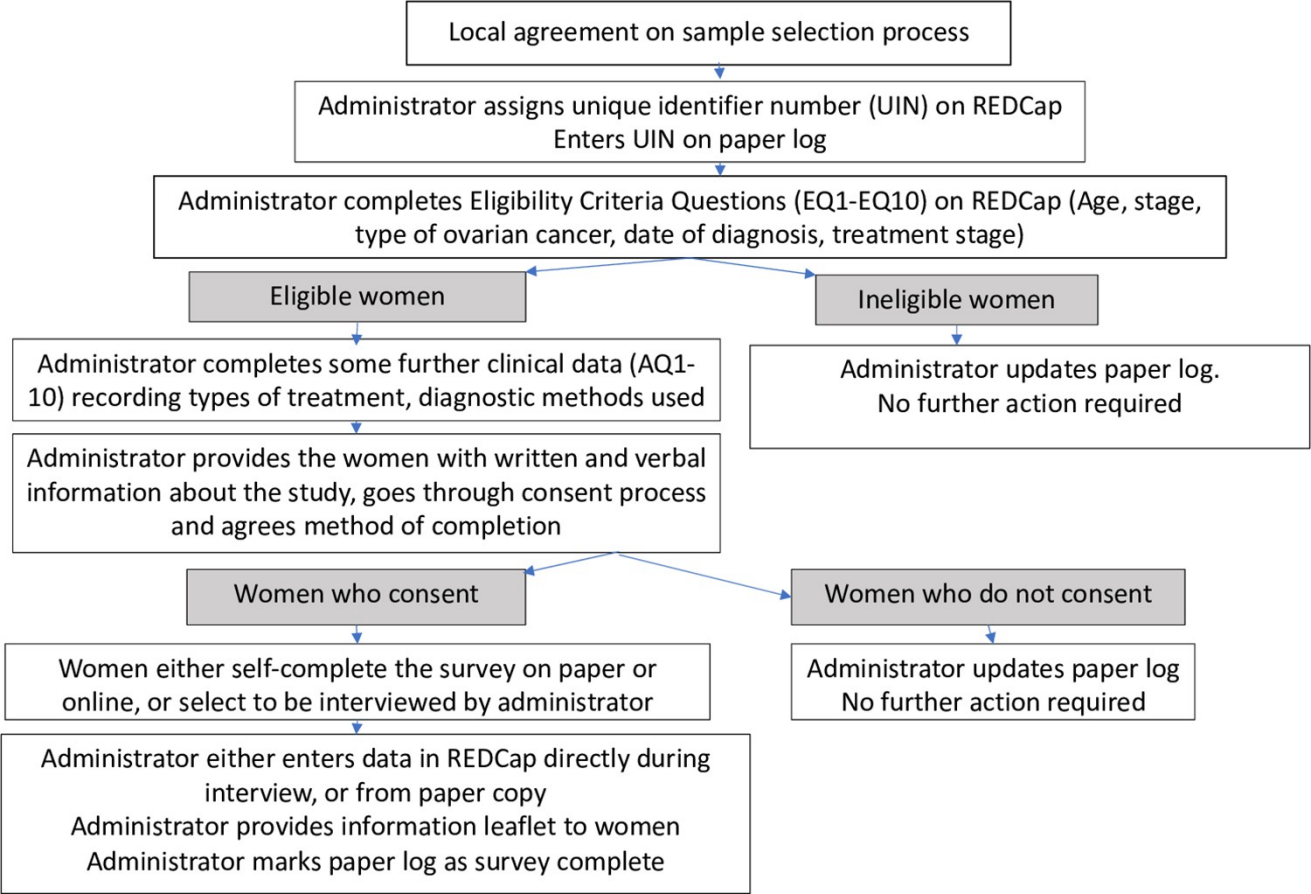
Flowchart outlining the recruitment and data collection process.

Local teams are encouraged to supply the women with some written as well as verbal information about ovarian cancer, including at least two sources of potential support and information, be they non-governmental organisations (NGO), charities, or other groups (S6 File). The process is summarised in Fig 3.

### Data management

A paper log is created at each site, recording women with ovarian cancer considered for the study. It lists their name, UIN, eligibility, consent status and whether they went on to complete the survey. This is the only document that has identifiable information about participants, and the protocol requires it be kept in a locked cabinet by the LSL at the local site along with any paper copies of consent and survey answers. These records will be destroyed by the LSL six months after data collection closes. REDCap does not collect the patient’s name, nor does the paper copy of the survey have the patient’s name recorded on it.

Where surveys are completed on paper, or by interview, the administrator is responsible for entering relevant data onto REDCap using a protected login and password. This contains only non-identifiable information and is stored on the Amazon Cloud. All CLCs, LSLs and administrators involved must adhere to local or national guidelines on data privacy, as well as those included in the protocol. LSLs and administrators will only see their local site data, while the CLC can view all relevant data in their country, with the ability to download and export their non-identifiable data and see basic summary charts and statistics. The global data set is stored on REDCap system, with a protected login and password, seen only by the Principal Investigator and study team. Within REDCap, the Principal Investigator can view data at site, country and global levels. When data is downloaded for any review or analysis, the files are password protected.

The dataset for each country will be cleaned, and the data reviewed for any potential local bias or problems by the Principal Investigator and CLC. A descriptive summary report will then be produced for each country. The final dataset will then be exported from REDCAp and analysed using conventional statistical software, such as Stata or R.

### Safety considerations

This is a low-risk observational study, however, there remains a small but significant risk that participants will become uncomfortable when being questioned about their ovarian cancer experiences. Study teams are alerted to this and advised to have contact details of appropriate resources for the women. This may include referral for counselling, to a social worker, psychologist or to an NGO for support. The women will not be asked to complete the survey on the day they are given their diagnosis. Training is provided for the administrators, on administering the questionnaire, and on respectful conduct with the women.

### Data analysis

In our analysis, we seek to examine variation in key metrics (such as stage of ovarian cancer, time to diagnosis and levels of awareness) by country, region and by the socio-economic country group. In addition, we will explore associations between patient and country characteristics and key outcome measures. Table 3 gives some examples of the outcome variables and expected areas for further analysis.

**Table 3:** Outcome variables and expected further analysis.

Outcome variables		
• Access to chemotherapy • Access to optimal cytoreductive surgery • Access to genetic testing • Level of ovarian cancer knowledge • Time to seek help • Time from seeking help to obtaining a diagnosis • Time from diagnosis to treatment • Information needs •Impact on family finances •Priorities for action		
**Further analysis**		
**Outcome variables**	**Predictor variables**	**Possible confounders**
Access to chemotherapy	Age on diagnosis Family income Distance to hospital Who pays for ovarian cancer care Resource level of participating site	Stage of ovarian cancer Date of diagnosis Use of neoadjuvant chemotherapy
Access to optimal cytoreductive ovarian cancer	Age on diagnosis Family income Distance to hospital Who pays for ovarian cancer care Resource level of participating site	Date of diagnosis Use of neoadjuvant chemotherapy
Levels of awareness of ovarian cancer	Education level Family income level Distance to hospital Family history	
Time to seek help for symptoms	Age Education level Family income level Who pays for ovarian cancer care First person consulted re symptoms Number of visits prior to diagnosis Level of concern over symptoms Type of symptoms Number of symptoms	

We will perform descriptive and inferential statistics, overall and by country and economic grouping using statistical softwares such as STATA and R. Categorical variables will be summarised with counts and percentages, with Chi Squared Tests or Fisher’s Exact Test used to compare proportions between groups. Continuous variables, such as time to diagnosis, will be examined graphically and summarised using appropriate metrics (mean and standard deviation or median and interquartile range depending on the distribution of the variable data). Tests, such as Kruskal-Wallis, will be applied to continuous variables. Outlying countries will be identified and described.

Appropriate regression analysis approaches will be used to examine assocations between independent and outcome variables. Binary, ordinal or nominal regression will be used depending on the nature of the outcome variable. Univariable regression will be employed to examine individual associations and multivariable regression to examine combined assocations between predictor and outcome variables.

The two free-text answers will be translated, coded and analysed using qualitative content analysis to identify categories and possible themes.

Each country will receive a bespoke report summarising their results, how these compare with the average for all participants at a global and/or regional level, and provide information about the context of ovarian cancer care in their country obtained through interviews and surveys with the CLCs. CLCs will ‘own’ the country specific data and will be encouraged to share their national findings, and utilise them for healthcare planning, further research and engagement activities in their country.

### Ethical considerations and declarations

The study is being conducted in accordance with the principles of the Declaration of Helsinki (2013). The protocol, survey, informed consent form, and participant information sheet have been submitted to an appropriate research ethics committee, health research authority as required in each country/site, and host institution(s) for written approval. Where necessary the CLC has submitted and obtained approval from the above parties and the Principal Investigator for all substantial amendments to the original approved documents. In three of the 24 countries, local ethics committees have made a requirement that patients be compensated for their time. This compensation level varies between US$3 and US$8 by country and is to be given on completion of the survey, with each patient receiving the same amount within a given country.

### Status and timeline of the study

At the time of writing, 24 countries are progressing with the study, with 19 actively collecting data on the REDCap platform. Some countries will stop collecting data in September 2023, but others, for whom translations, ethics approvals or local circumstances meant delayed starts or slow progress, data collection will continue up until the end of December 2023.

## Discussion

There are a number of strengths to the study. Participating countries will have the opportunity to compare key challenges and opportunities to improve survival and quality of life for women with ovarian cancer with other LMICs. For some of the teams, this is the first time they have contributed to research at their centre or been involved in collaborations with others in their country or internationally. For several countries, it provides the first dataset on ovarian cancer in their country, which could be used to inform clinical and policy decisions, drive academic research and develop interventions to improve outcomes for women with ovarian cancer. In addition, for women with ovarian cancer in many of the participating countries, this is the first time their experiences and opinions have been sought for research, bringing visibility to their plight and providing the opportunity to help other women in the same situation. Already there has been the formation of Ovarian Cancer Malaysia involving a patient member of the OC and the CLC, and a survivorship group is being planned in Kenya. The study also provides clinical staff with insights about important patient perspectives beyond treatment.

There are several limitations to the study. First, it will only capture experiences from women who have been able to afford or access a diagnosis and subsequent treatment. In some participating countries, there is just one centre that diagnoses and treats the condition so there are many economic, geographic, societal and cultural barriers preventing symptomatic women from getting medical help. Second, the varied approach of sample selection may naturally introduce biases, but it is not feasible to apply a uniform approach in all participating sites. To account for this, sensitivity analyses will be conducted by including and excluding certain selection methods where distinct patterns are observed. Third, the minimum sample size per country will only provide a broad overview with wide confidence intervals at a standalone country level. However, the regional and global analysis will be able to determine how a country differs from the others in the study with greater accuracy, helping focus priorities for action. Finally, by running the study through known contacts of the Coalition and IGCS, there may be biases in terms of women’s experiences, particularly around levels of care, as the CLC and LSL have access to training and networks of specialist clinicians.

The results of the study with analyses at global, regional, and thematic levels, will be disseminated through peer reviewed papers, with presentations at key relevant conferences. In this way it will contribute to the knowledge base on ovarian cancer globally, and in participating LMICs. It will be supported by a media outreach campaign. Each of the CLCs will be encouraged to submit papers for publication and presentations at national and regional level in local languages to extend the reach of the study and its findings. Subject to funding, the Coalition plans to work directly with some of the country teams to develop action plans and engagement with health policy makers, which will be informed by the study findings. Some of the participating countries plan to continue data collection, subject to the appropriate permissions, and/or adapt the study for other disease areas. By publishing the protocol, survey, and findings, the authors also hope to encourage other countries to consider running the study themselves.

## Supporting information

S1 Checklist Inclusivity in global research. (DOCX)

S1 File Every woman study survey. (PDF)

S2 File Kenya protocol. (PDF)

S3 File Country lead interview questions. (PDF)

S4 File Country lead survey. (PDF)

S5 File Additional information for women. (PDF)

S6 File Eligibility criteria and clinical data instrument. (PDF)
