# Supplementary material for: The every woman study™ low- and middle-income countries edition protocol: A multi-country observational study to assess opportunities and challenges to improving survival and quality of life for women with ovarian cancer
Source: PLoS One. 2024 May 29;19(5):e0298154. doi: 10.1371/journal.pone.0298154 (PMC11135759; doi:10.1371/journal.pone.0298154)
Supplement: S1 File — (PDF) [file pone.0298154.s002.pdf]

THE **EVERY WOMAN** STUDY™
LOW- and MIDDLE-INCOME EDITION

WORLD OVARIAN CANCER COALITION   IGCS INTERNATIONAL GYNECOLOGIC CANCER SOCIETY

A JOINT INITIATIVE FOR WOMEN

INSERT HOSPITAL LOGO AND/OR HOSPITAL NAME

The Every Woman Study: Patient Information and Consent

This hospital is participating in a research study called **The Every Woman Study™** in low- and middle-income countries, and we would like to invite you to participate. The aim of the Study is to identify the challenges and opportunities to improve survival for women with ovarian cancer. It does not involve you trying new medicines or procedures but is a survey that will take at least 20 minutes to complete. It will ask about any symptoms you experienced, how you were diagnosed, your needs since diagnosis, and where you would like to see improvements made in the diagnosis and care of women with ovarian cancer.

You do not have to take part in the Study. It is entirely voluntary, so you can choose not to take part. The care you receive here will not be affected in any way. If you decide to take part and then change your mind, that is also fine.

Your answers will be joining those of over two thousand women from 31 countries around the world, and together, they will help prioritise improvements to the diagnosis and care of women with ovarian cancer at this hospital, in this country, and more widely in other low- and middle-income countries.

The Study is supported and funded by the World Ovarian Cancer Coalition and the International Gynecologic Cancer Society. These two global non-profits are strategic advocacy partners who together have hundreds of members, patient survivors, and partner organisations around the world. Through their own work and with this joint Study, both organizations are committed to ensuring that women who have ovarian cancer get the best possible care, no matter where they live.

Risks and benefits of taking part
There is no direct benefit to you in taking part in the Study. However, many women find it beneficial to share their experiences with others and the findings of the study may help this hospital improve its care in the future and may impact on policy changes benefitting women with ovarian cancer in your country.

Occasionally some women may find it upsetting to think about their experiences, but please be reassured that the doctors and nurses will help where they can and will provide you with details of people or organisations you can contact if you need further support. You do not have to provide an answer to every question, but we hope you will want to provide as many answers as possible.
You may be asked to fill in the survey either online, or on paper in the hospital or clinic setting. Alternatively, you may be given the option of filling in the survey

online from home, or of having someone ask you the questions, and that person making a record of your answers.

Whilst we will have to retain a separate paper record of your name and unique identifier number, your name will not be uploaded to the Study electronic database with any of your answers. This means you will not be identifiable from your answers. In December 2023 we will destroy any physical information that links your name to your Study number. If you change your mind about taking part in the Study, you can withdraw your consent by telling a member of your hospital team. They will use your unique identifier number to extract your answers from the study. You can do this at any point up to 30th June 2023.

Please enter your Unique Identifier Number (UIN)............................ *(paper copy only)*

Please select only ONE of the following statements

- I have understood the purpose of this Study and my role in it. I give my consent that the researchers may use my answers (not linked to my name) as part of the study.
- I have understood the purpose of this Study and my role in it. I consent to the presence of the survey administrator for the purpose of facilitating my participation in the study
- I still have some questions about the Study I would like answered before I decide
- I have understood the purpose of this Study, and what you are asking me to do. However, I do not consent to taking part.

  If you would like to share the reason for this, please do so here.

Patient signature and date *(for paper copy only)*

Administrator signature and date *(for paper copy only)*

UNIQUE IDENTIFIER NUMBER (UIN)..

Q1	Are you filling in this questionnaire about your own experience of ovarian cancer?<br><br>Yes – **GO TO Q2**<br>No – **GO TO Q1A**
Q1a (if answered No to Q1)	If you are filling in this questionnaire with someone who has ovarian cancer, what is your role?<br><br>Doctor/Trainee Doctor/Nurse/Trainee Nurse/Researcher/Social Worker/Other (please specify)<br><br>The questions that follow are those that you should ask the woman with ovarian cancer about her experience. Other than a simple explanation, please ensure you do not suggest which answers they should give.
Q2	Please indicate your ethnicity. By ethnicity we mean your feeling of belonging and attachment to a distinct group of a larger population that shares their ancestry, colour, language, or religion. You may select more than one option<br><br>***Each Country Lead Clinician will be asked to supply a standard but useful list from their own country, and include /Mixed/Other/I'd rather not say.***
Q3	On average, how long does it take you to travel to the hospital where you are seen in connection to your ovarian cancer?<br><br>Under 15 minutes<br>15-30 minutes<br>30-60 minutes<br>1-2 hours<br>2-5 hours<br>Over 5 hours<br>Over 24 hours
Q4	What is the **highest** level of education you have achieved?<br><br>No formal education<br>Primary education (childhood, elementary, early years, ISCED level 1)<br>Secondary education (middle school, high school, ISCED levels2,3)<br>Post-secondary, non-tertiary education (technical college, community college, ISCED levels 4, 5)<br>Tertiary education (university or similar professional skills, ISCED level 6)<br>Post-graduate education (University Master's or Doctorate or senior professional qualification, ISCED levels 7 or 8)
Q5	Just before you were diagnosed with ovarian cancer, were you?<br><br>Married or living with a partner<br>Widowed<br>Divorced or Separated

	In a relationship but not living together<br>Single (never married)<br>Prefer not to say
Q6	Just before you were diagnosed, in your view what was your household income?<br><br>Below average for your country<br>Average for your country<br>Above average for your country<br>Prefer not to say
Q7	Around the time of your diagnosis, did you have any caring responsibilities for others in your family? **TICK ALL THAT APPLY**<br><br>Yes, a child or children under the age of 18<br>Yes, an elderly relative<br>Yes, a sick partner<br>Other<br>No, I have not had caring responsibilities
Q8	Just before you were diagnosed with ovarian cancer, were you?<br><br>In paid full-time employment<br>In part-time employment<br>Self-employed<br>Not in paid or self-employment<br>Retired<br>Other
Q9	How have you paid for your medical care/ovarian cancer care up to this point?<br>**TICK ALL THAT APPLY**<br><br>Private Medical Insurance<br>State Medical Insurance<br>I have paid<br>Family members have paid<br>I have raised money to cover costs by crowdfunding<br>I have sought financial help from a Non-profit/Non-Governmental Organisation/Charity<br>Treatments for ovarian cancer are free<br>Diagnostic tests for ovarian cancer are free
Q10	In relation to your gynaecological history before you were diagnosed with ovarian cancer, **TICK ALL THAT APPLY**<br><br>I had entered the menopause where I no longer have monthly periods<br>I have never given birth to a child<br>I have given birth once

	I have given birth twice<br>I have given birth three or more times<br>I breast fed my children<br>I used the oral contraceptive pill for up to 5 years<br>I used the oral contraceptive pill for between 5 and 10 years<br>I received treatment for infertility

**Family History**	
Q11	Have any of the following family relatives (i.e. blood relatives on either your mother or your father's side of the family) had ovarian cancer?<br>**TICK ALL THAT APPLY**<br><br>Mother<br>Daughter<br>Sister<br>Aunt<br>Cousin<br>Grandmother (mother's side)<br>Grandmother (father's side)<br>Other more distant relatives (mother's side)<br>Other more distant relatives (father's side)<br>No, none of my close family have been affected<br>I do not know or cannot remember
Q12	Have any of the following family relatives (i.e. blood relatives on either your mother or your father's side of the family) had breast cancer?<br>**TICK ALL THAT APPLY**<br>Mother<br>Father<br>Daughter<br>Son<br>Sister<br>Brother<br>Aunt (either side)<br>Uncle (either side)<br>Cousin (either side)<br>Grandmother (either side)<br>Grandfather (either side)<br>No, none of my close family have been affected<br>I do not know or cannot remember
Q13	Are you aware that having blood relatives with ovarian, breast, pancreatic, prostate, bowel or womb cancer might increase the risk of a woman developing ovarian cancer?<br><br>Yes<br>No<br>Not Sure

**Leading up to diagnosis**	
Q14	Before you were diagnosed with ovarian cancer, how much if anything did you know about ovarian cancer?<br><br>I had heard of it and knew something about it<br>I had heard of it but did not know anything about it<br>I had never heard of it<br>I do not know or cannot remember
Q15	Before your diagnosis of ovarian cancer, which, if any, of the following symptoms did you experience?<br>**TICK ALL THAT APPLY**<br>Pain in the abdomen (stomach)<br>Urinary frequency<br>Difficulty eating<br>Changes in bowel habit (e.g., diarrhoea or constipation)<br>Extreme fatigue<br>Increased abdominal size<br>Feeling full<br>Unexplained weight loss<br>Urinary urgency<br>Pain in the pelvis<br>Persistent bloating<br>Other symptoms<br>**(IF YOU SELECTED ANY OF THE ABOVE OPTIONS GO TO Q17)**<br>None of the above **(GO TO Q16)**
Q16 only if they answer None of the Above in Q15	As you **did not** experience symptoms outlined in the previous question before you were diagnosed, what led to your diagnosis?<br>**TICK ALL THAT APPLY**<br><br>A routine examination<br>A routine scan<br>A routine blood test<br>The cancer was discovered whilst I was being treated for something else<br>Other<br>**NOW GO TO Q27**
Q17 for women who had symptoms listed in Q15	When did you first notice any of the symptoms selected above?<br>Please enter the month, and the year.<br>If you do not remember, please leave this blank.<br><br>........................ Month symptoms began<br>....................... Year symptoms began
Q18	How concerned about your symptoms were you before your diagnosis?

	Concerned<br>Somewhat concerned<br>Not very concerned<br>Not at all concerned<br>I do not know or cannot remember
Q19	Did you seek advice from someone about your symptoms, for example a doctor, nurse, pharmacist or healer?<br><br>Yes **(GO TO Q21)**<br>No **(GO TO Q20)**
Q20<br>If they answer no to Q19	As you did not seek advice from someone about your symptoms, what led to your diagnosis?<br>**TICK ALL THAT APPLY**<br><br>A routine examination<br>A routine scan<br>A routine blood test<br>The cancer was discovered whilst I was being treated for something else<br>Other<br>**(GO TO Q26)**
Q21	Which type of person, other than a family member, did you **first** seek advice from, about your symptoms?<br>**SELECT ONLY ONE ANSWER**<br><br>A local healer<br>An alternative health practitioner<br>A family doctor<br>A gynaecologist<br>A gynaecologic oncologist (a doctor specialising in the treatment of ovarian cancer)<br>A gastroenterologist<br>An emergency room or accident and emergency doctor<br>A nurse<br>A pharmacist<br>Someone else
Q22	In addition to the first person, you sought advice from about symptoms, who else did you visit or talk to about your symptoms in the time before you were diagnosed?<br>**TICK ALL THAT APPLY**<br><br>A local healer<br>An alternative health practitioner<br>A family doctor<br>A gynaecologist<br>A gynaecologic oncologist (a doctor specialising in the treatment of ovarian cancer)

	A gastroenterologist<br>An emergency room or accident and emergency doctor<br>A nurse<br>A pharmacist<br>A family member<br>Someone else<br>No one else
Q23	When did you first visit **a medical doctor** about your symptoms?<br><br>Please enter the month, and the year.<br>If you do not remember, please leave this blank.<br><br>...................... Month visited Medical Doctor for Symptoms<br>...................... Year visited Medical Doctor for Symptoms
Q24	In your view, how seriously did the medical doctor you first saw take your concerns about symptoms?<br><br>Very seriously<br>Fairly seriously<br>Not very seriously<br>Not at all seriously<br>I do not know or cannot remember
Q25	Approximately how many times did you talk or visit medical doctors (any type of doctor, not just the one you saw first) before you were told you had ovarian cancer?<br><br>If you cannot remember, please leave this blank<br><br>___ (Enter a number)
Q26	Do you feel that the time from you first experiencing symptoms to being diagnosed with ovarian cancer could have been shortened?<br><br>Yes<br>No<br>Not sure<br><br>Please use the COMMENT box to let us know how you think the time could have been shortened (for example doctor delay, health system delay such as having to wait for tests or appointments) or factors that may have delayed you seeking help.

	COMMENTS
Q27	When were you told by a medical doctor that you had ovarian cancer?<br><br>Please enter the month, and the year.<br>If you do not remember, please leave this blank.<br><br>……………….. Month told by a medical doctor you had ovarian cancer<br>……………….. Year told by a medical doctor you had ovarian cancer

**Treatments for ovarian cancer**	
Q28	When did you first begin any treatment for ovarian cancer, such as surgery or chemotherapy?<br><br>If you cannot remember, or have not had any treatment please leave this blank<br><br>……………….Month treatment began<br>……………….Year treatment began
Q29	In deciding what, if any, treatments you will have to control your ovarian cancer or deal with side effects from treatment, which of the following will affect your decision?<br>**TICK ALL THAT APPLY**<br><br>The opinion of the doctor<br>The opinion of my family<br>I will make up my own mind<br>The cost of treatment drugs<br>Other costs associated with treatment such as transport or accommodation<br>The chance to cure or extend my life<br>The side effects of treatment<br>**(IF YOU HAVE SELECTED ANY OF THE OPTIONS ABOVE PLEASE CONTINUE TO Q 30)**<br>None of the above **(GO TO Q31)**

Q30	Which of the statements you selected in the previous question is **most** important to your decision making?<br>**SELECT ONLY ONE ANSWER**<br><br>The opinion of the doctor<br>The opinion of my family<br>I will make up my own mind<br>The cost of treatment drugs<br>Other costs associated with treatment such as transport or accommodation<br>The chance to cure or extend my life<br>The side effects of treatment
Q31	Overall, do you feel as involved as you would like to be, in decisions about your treatment throughout your cancer care, such as deciding which treatments, or whether to have any treatment?<br><br>Yes always<br>Yes most of the time<br>Only some of the time<br>Not at all<br>I do not know or cannot remember
Q32	Which, if any, of the following side effects of treatment for ovarian cancer have been **most** difficult to deal with at any point since you started treatment?<br>**SELECT UP TO TWO ANSWERS**<br><br>I have not had any treatment (GO TO Q35)<br>Tiredness or fatigue<br>Anxiety<br>Feeling or being sick (nausea or vomiting)<br>Diarrhoea<br>Loss of appetite<br>Inability to fight infection<br>Hair thinning or hair loss<br>Dry skin<br>Sore mouth<br>Tingling or numbness in hands or feet<br>Sleep loss<br>Metal taste in mouth<br>Constipation<br>Allergic reaction<br>Joint aches or pains<br>Swelling<br>Muscle aches or pains<br>Menopausal symptom<br>Complications following surgery<br>Another side effect<br>**(IF YOU HAVE SELECTED ANY OF THE ABOVE GO TO Q33)**<br>No side effects in particular **(GO TO Q35)**

Q33 if they had side effects	Would you describe these most difficult side effects as 'long-term', in other words, persisting for some weeks or months after treatment has ended?<br><br>Yes<br>No<br>I am still in treatment so cannot say if they are long term<br>I am not sure
Q34	Have health professionals been able to help reduce the impact of side effects with other medicines, or advice and information?<br><br>Yes very much so<br>Yes to some extent<br>Not at all<br>I did not seek help, or I did not need to seek help about side effects<br>I do not know or cannot remember
Q35	Have you at any time since your diagnosis, used herbal, complementary, or alternative treatments not prescribed by your doctor at the hospital, to try and control your ovarian cancer, or any pain caused by your ovarian cancer?<br><br>Yes all the time<br>Yes at some point<br>No, but I have thought about it<br>No, not at all<br>I do not know
Q36	Have you ever wanted to get another opinion from a second doctor about your ovarian cancer and treatment?<br><br>No<br>Yes and I have been able to<br>Yes but I don't know who to ask<br>I have not thought about it

**Emotional Support needs**	
Q37	Up to now, have there been times when **you** have felt in need of emotional support?<br>**TICK ALL THAT APPLY**<br><br>At the time of diagnosis<br>During treatment<br>After treatment ended<br>When the cancer returned<br>When told the cancer was not curable<br>Other<br>**(IF YOU HAVE SELECTED ANY OF THE ABOVE GO TO Q38)**<br>I have not needed any emotional support **(GO TO Q 42)**

Q38<br>If they have needed emotional support	Are there particular issues you have faced?<br>**TICK ALL THAT APPLY**<br><br>Fear of the cancer returning<br>Fear that treatment will not work<br>Fear of dying<br>Difficulty with getting back to 'normal life' after treatment<br>Partner or spouse leaving<br>Other issues relating to family and friends<br>Feelings of isolation<br>Feeling unable to talk to others<br>Loss of fertility<br>Regaining sexual intimacy with a partner<br>Coping with the menopause<br>Dealing with stigma because of the cancer diagnosis<br>Other<br>**(IF YOU HAVE SELECTED ANY OF THE ABOVE GO TO Q 39)**<br>None in particular **(GO TO Q41)**
Q39	Which issue have you found most challenging?<br>**SELECT ONLY <u>ONE</u> ANSWER**<br><br>Fear of the cancer returning<br>Fear that treatment will not work<br>Fear of dying<br>Difficulty with getting back to 'normal life' after treatment<br>Partner or spouse leaving<br>Other issues relating to family and friends<br>Feelings of isolation<br>Feeling unable to talk to others<br>Loss of fertility<br>Regaining sexual intimacy with a partner<br>Coping with the menopause<br>Dealing with stigma because of the cancer diagnosis<br>Other<br>None in particular
Q40	In connection to your emotional support needs, have you?<br>**TICK ALL THAT APPLY**<br><br>Asked for help from a doctor or nurse<br>Been offered help from a doctor or nurse<br>Been offered psychotherapy from a psychologist<br>Asked for help from family or friends<br>Been offered help from family or friends<br>Asked for help from a charity or non-governmental organisation (NGO)<br>Been offered help from a charity or non-governmental organisation (NGO)<br>Asked for help from a religious organisation/person<br>Been offered help from a religious organisation/person

	None of the above
Q41	Have you been able to get the emotional support you needed?<br><br>Yes<br>Yes, to some extent<br>Not enough<br>None
Q42	Have you ever met and talked to another woman, or group of women with ovarian cancer since your diagnosis? **TICK ALL THAT APPLY**<br><br>Yes, in person<br>Yes, in person as part of a cancer group<br>Yes online (social media, chat room, forum)<br>Yes, on the telephone<br>No
Q43	Would you like to be able to meet with other women who have ovarian cancer?<br><br>Yes<br>No<br>Not sure

Practical Support needs	
Q44	Which, if any of the following forms of practical support do you feel you need, or have you needed, because of your diagnosis of ovarian cancer?<br>**TICK ALL THAT APPLY**<br><br>Help with daily chores at home (e.g., shopping, cleaning, preparing food, gardening)<br>Help with personal care (e.g., getting dressed, washed, wound care<br>Home adaptations (e.g., using a wheelchair, handrails)<br>Help caring for dependants (parents, siblings, children)<br>Help with transport including travel to and from hospital<br>Financial support<br>Other<br>**(IF YOU HAVE SELECTED ANY OF THE ABOVE GO TO Q45)**<br>I have not needed any practical support **(GO TO Q47)**
Q45 if they had support needs	Which, if any, of the following forms of practical support have you received following your diagnosis and treatment for ovarian cancer.<br>**TICK ALL THAT APPLY**<br><br>Help with daily chores at home (e.g., shopping, cleaning, preparing food, gardening)<br>Help with personal care (e.g., getting dressed, washed, wound care<br>Home adaptations (e.g., using a wheelchair, handrails)<br>Help caring for dependants (parents, siblings, children)<br>Help with transport including travel to and from hospital<br>Financial support<br>Other
Q46	Which group or person has given you the most practical support?<br>**TICK ALL THAT APPLY**<br><br>Family member(s)<br>Friends<br>A charity<br>A non-governmental organisation (NGO)<br>A government agency<br>A religious organisation or person<br>A carer<br>Someone else<br>No one in particular
Q47	Has having a diagnosis of ovarian cancer had an impact on your financial situation?<br><br>Yes to a great extent<br>Yes to some extent<br>Not much

	**(IF YOU SELECTED ANY OF THE ABOVE GO TO Q 48)**<br>Not at all **(GO TO Q49)**<br>I would prefer not to say **(GO TO Q49)**
Q48 if there has been some financial impact	In what way has your financial situation been impacted by your diagnosis? **TICK ALL THAT APPLY**<br><br>I have been unable to work<br>My household income has dropped below what we need to live on<br>I or my family have had to pay for treatment or tests<br>I or my family have had to spend extra money on travelling to or accommodation near the hospital<br>I have had to ask for financial help from family members<br>I have had to ask for financial help from a charity or NGO<br>I now find it hard to find the money for food, rent and bills<br>My partner has been unable to work because they care for me<br>Other

**Information needs**	
Q49	Since being diagnosed, have you felt in need of information about ovarian cancer? **TICK ALL THAT APPLY**<br><br>Ovarian cancer in general<br>Treatments for ovarian cancer<br>Coping with long term side effects of treatment<br>Clinical trials<br>Genetic testing<br>Living with ovarian cancer<br>Reducing anxiety<br>Symptoms that might indicate a recurrence<br>Survival rates<br>Managing ovarian cancer that can no longer be treated<br>How to talk to family and friends<br>Other<br>**(IF YOU HAVE SELECTED ANY OF THE ABOVE GO TO Q50)**<br>I have not needed any information **(GO TO Q53)**
Q50 (if they needed info)	Have you found the information you needed when you needed it?<br><br>Yes always<br>Yes sometimes<br>Just a little information<br>**(IF YOU HAVE SELECTED ANY OF THE ABOVE GO TO Q51)**<br>Not at all **(GO TO Q52)**
Q51 If they found some	What have been the most important sources of information for you?<br>**SELECT UP TO TWO ANSWERS**

of the information	Your doctor<br>Your nurse<br>Another health professional<br>A charity<br>A non-governmental organisation<br>A Government Agency<br>A website<br>An ovarian cancer support group (online or face to face)<br>A cancer support group (online or face to face)<br>Other women who have had ovarian cancer<br>Radio<br>Other<br>None in particular
Q52	Have you ever searched for information on the internet about your diagnosis?<br>**TICK ALL THAT APPLY**<br><br>Yes, and found good information in my language<br>Yes, but did not find any good information in my language<br>Yes, but did not find useful information<br>Yes, but found information that made me scared<br>Yes, but I could not find information in my language<br>I do not have easy access to the internet<br>Other<br>No, I have not used the internet to search for information
Q53	If this hospital were able to provide women with information about living with ovarian cancer, what do you think it should include?<br>**TICK ALL THAT APPLY**<br><br>Information about treatments and diagnosis<br>Information about living with ovarian cancer and what to expect<br>Information on how to manage physical and mental health<br>Managing ovarian cancer that can no longer be treated<br>Sources of local or national support<br>A way to meet other women with ovarian cancer in person or online<br>The hospital already supplies the information I need<br>Other<br>I would not like them to provide information

Final questions	
Q54	At this point in time, what factors would mean you have a good quality of life given you are living with ovarian cancer?<br>**TICK ALL THAT APPLY**<br><br>Feeling physically well<br>Feeling mentally well<br>Being able to work<br>Being able to care and support your family<br>Not being a burden on your family<br>Being able to maintain or have a physical relationship with your partner<br>Being able to engage in hobbies and activities<br>Being able to socialise<br>Having a positive self-image<br>Feeling in control of your life<br>Being able to return to 'normal'<br>To be free from the fear of cancer<br>Other
Q55	Do you feel COVID-19 pandemic has had an impact on your treatment and feelings in relation to your cancer? **TICK ALL THAT APPLY**<br><br>I am or have been scared to visit a hospital<br>I worry about catching COVID-19<br>I am worried the pandemic might affect my chance of getting treatment<br>I have worried about whether I should get a vaccine<br>The COVID-19 pandemic has affected my treatment<br>The COVID-19 pandemic contributed to a delay in my diagnosis<br>It has made me feel more isolated<br>Other<br>No, the pandemic has not impacted me in this way
Q56	Would you be willing to consider taking part in a clinical trial, comparing new treatments to the normal standard of care, if they were available at this or another hospital?<br>**TICK ALL THAT APPLY**<br><br>I would not be interested<br>I would like to find out more information first<br>I would be interested in taking part in a trial at this hospital<br>I would consider taking part in a clinical trial even if it meant travelling to another hospital
Q57	If money could be invested in improving diagnosis and care of women with ovarian cancer in your country, which areas are most in need of improvement?<br>**SELECT UP TO THREE OPTIONS**

	Development of a screening programme to detect the disease before symptoms develop<br>Ensuring women have free access to diagnostic tests<br>Reducing delays in diagnosis<br>Raising awareness of ovarian cancer and the symptoms<br>Ensuring women have free access to treatments<br>Increasing the number of experienced surgeons<br>Getting access to new drugs that are approved in high income countries<br>Ensuring women at risk of ovarian cancer are identified due to their family history<br>Ensuring women can access clinical trials<br>Funding for research<br>Other
Q58	Do you feel your government could do more to help women with ovarian cancer live a long and good life?<br><br>Definitely<br>Yes to some extent<br>Not really<br>They could not do any more
Q59	Is there something that is particularly important to you about your experience of ovarian cancer that you would like to share with the study team?

Thank you so much for sharing your experiences. If you have any questions or concerns, please speak to the person who invited you to take part in this survey.
