# Supplementary material for: The every woman study™ low- and middle-income countries edition protocol: A multi-country observational study to assess opportunities and challenges to improving survival and quality of life for women with ovarian cancer
Source: PLoS One. 2024 May 29;19(5):e0298154. doi: 10.1371/journal.pone.0298154 (PMC11135759; doi:10.1371/journal.pone.0298154)
Supplement: S2 File — (PDF) [file pone.0298154.s003.pdf]

THE EVERY WOMAN STUDY IN KENYA (EWS): IDENTIFYING CHALLENGES AND OPPORTUNITIES TO IMPROVE SURVIVAL AND QUALITY OF LIFE FOR WOMEN WITH OVARIAN CANCER

Version 1.3, 24.04.2022

Principal Investigators:

Afrin Fatima Shaffi, MBBS, MMed
Dept. of Reproductive Health
Moi University, School of Medicine
Kenya
Ph: +254797167864
afrinfatima2014@gmail.com

Frances Reid
World Ovarian Cancer Coalition
205 – 145 Front Street East
Toronto, Ontario Canada M5A 1E3
+44 7957 495958
frances@worldovariancancercoalition.org

Co-investigators:

Investigators	Institution	Role
Anisa Mburu	Aga Khan Hospital	Co-investigator, Kenya
Benjamin E.Odongo	Kenyatta University	Co-investigator, Kenya
Amina Rashid	Moi University	Co-investigator, Kenya

Short title: EWS - KENYA

Funder: The World Ovarian Cancer Coalition and the International Gynecologic Cancer Society

Project duration: June 2022-June 2023

Please declare any/no potential conflicts of interest.

Confidentiality Statement
This document contains confidential information that must not be disclosed to anyone other than the Sponsor, the Investigator Team, host organisation, and members of the Research Ethics Committee, HRA (where required) unless authorised to do so

Contents

1. Synopsis 4
2. List of Abbreviations 5
3. Background 6
4. Rationale/Justification 7
5. Objectives 7
6. Methods 8
  6.1 Study setting 8
  6.2 Study design 8
  6.3 Study population 8
  6.4 Eligibility Criteria 8
  Exclusion Criteria 9
  6.5 Sample size calculation 9
  6.6 Sampling 9
  6.7 Study Procedure and data collection 9
  6.8 Research Assistant 11
  6.9 Recruitment 11
  6.10 Privacy and Confidentiality 11
  6.11 Data Management 12
  6.12 Analysis 13
7. Assumptions and limitations 14
8. Ethical and Regulatory Considerations 15
  8.1 Informed consent 15
  8.2 Institutional Review Board 15
  8.3 Reporting 16
  8.4 Potential Risks and Benefits 16
  8.5 Discontinuation/Withdrawal of Participants from Study 16
9. Definition of End of Study 17
10. Funding 17
11. Dissemination of Research Findings 17
12. References 17
13. Time Frame 17
14. Budget 18
15. Signatures of Principal Investigators 18

16. Appendices 19
16.1. Appendix I 19
16.2. Appendix II: Patient Study Information and Consent Form (English version) 24

1. Synopsis

**Study title**	The Every Woman Study in Kenya: Identifying challenges and opportunities to improve survival and quality of life for women with ovarian cancer
**Internal ref. no. / short title**	EWS - KENYA
**Study design**	A low-risk observational cross-sectional study using a standardised questionnaire derived from a previously published set of questions exploring the experiences of women with ovarian cancer (The Every Woman Study^{TM}), based on symptoms, route to diagnosis, treatment and quality of life.<br><br>The aim of the study is to establish the first ever patient experience evidence base of women with ovarian cancer living in Kenya and a selection of low- and middle-income countries, and identify the key challenges and opportunities to improve survival and quality of life at national and continent levels.
**Study participants**	All eligible women attending Moi Teaching and Referral Hospital and Aga khan Hospital with incorporation of its satellite clinics, with ovarian cancer diagnosed within the previous 5 years of survey completion.
**Planned sample size**	Minimum sample size of 92, with a maximum of 330
**Study period**	1 year
**objectives**	To gather sufficient survey responses to make robust statements about the experiences of women with ovarian cancer in Kenya, and provide a baseline of evidence for those attending MTRH and Aga Khan Hospital, Mombasa in relation to their ovarian cancer<br><br>To explore the similarities and variations in the experience of women in Kenya compared to those in other low- and middle-income countries (LMIC), participating in this standardized survey.<br><br>To explore the context in which the women are being treated, and how this varies between participating countries, and to showcase the findings in the context of the final report.

2. List of Abbreviations

AQ	Administrator Questions (Participant Clinical Data, e.g., AQ1, AQ2 etc)
CL	Country Lead
EQ	Eligibility Criteria Questions (e.g., EQ1, EQ2 etc.)
EWS	Every Woman Study
GCP	Good Clinical Practice
HIC	High-Income Country
ICF	Informed Consent Form
IGCS	International Gynecologic Cancer Society
LMIC	Lower- and Middle-Income Countries
MTRH	Moi Teaching Referral Hospital
NGO	Non-Governmental Organisation
OC	Oversight Committee
OCP	Ovarian Cancer Patient
PIL	Participant/ Patient Information Leaflet
REC	Research Ethics Committee
SOP	Standard Operating Procedure
UIN	Unique Identifier Number
WOCC	World Ovarian Cancer Coalition

3. Background

In 2018, the World Ovarian Cancer Coalition (the Coalition), led by Principal Investigator and Programme Director Frances Reid, developed the Every Woman Study: a comprehensive and extensive survey for women within five years of their cancer diagnosis. The patient experience survey covered knowledge of ovarian cancer, family history, symptom experience, time and routes to diagnosis, care received including treatments and access to clinical trials, and quality of life issues such as long-term side effects, support and information needs and priorities for action.

Overseen by an Expert Advisory Panel, the 2018 survey was developed following thematic analysis of a range of guided interviews with women from around the world, and two stages of testing. The final online survey in 15 languages, taking over one hour to complete was shared by social media and patient advocacy groups and attracted some 1531 responses from 44 countries. Women self-selected to participate, if they had been diagnosed in the previous five years.

The published results[i] identified a set of six key challenges to overcome in order to improve the survival and quality of life for women diagnosed with the disease. Most of the respondents lived in high-income countries but despite this there was very wide variations in the extent to which each challenge was an issue. Reducing the variation can provide a route for progress. For example, in some countries women are more aware of symptoms, or more likely to visit a doctor about symptoms, but not in others. In some countries doctors are slow to act on symptoms or have varied approaches to diagnostic testing. Access to specialist surgery and drug treatments also can vary by and within countries, as can access to genetic testing and clinical trials. Women in different parts of the world have some common but some different support needs depending on their location, age and culture.

At the time the Expert Advisory Panel acknowledged that the respondents to the survey were on the whole, younger, wealthier and healthier than expected, and predominantly came from high-income countries, but none the less the results presented an astonishing and to date unique account of patient experience that has been instrumental in bringing together patient and clinical advocacy to drive change.

The lack of ovarian cancer patient experience from LMIC in the 2018 study and published literature was noted by the Expert Advisory Panel, and since that time the Coalition has partnered with the International Gynecological Cancer Society to develop a version of the survey for use in low- and middle-income country settings. Adaptation of the original survey was overseen by the Oversight Committee (OC) to retain validity. The OC comprises clinicians and advocates from each of the large geographic areas (South Asia, East Asia, Central Asia, Africa, North Africa and the Middle East, Latin America, and the Caribbean), in addition to global experts on cancer data and early detection.

Magnitude of the problem in Kenya

Ovarian cancer is the third most common cause of death among gynaecological cancer patients in Kenya and accounts for 3% deaths in these women (*Kenya, Globocan*, 2020). In Kenya, most cases of ovarian cancer are diagnosed at advanced stages III and IV of disease (Cheserem *et al.*, 2013). Over the last few years there have been a rise in the number of ovarian cancer cases and deaths in Kenya (Cheserem *et al.*, 2013), and between 2020 and 2040 Cases are predicted to rise from 1,130 per year to 2,600 per year (*Kenya, Globocan, 2020*). Kenya is facing the growing high demand for cancer treatments, but the nation's very limited supply capacity with respect to diagnosis and treatment poses serious health-care policy challenges to the government.

Multicentre Study

This proposal is being submitted for the Kenyan study site, which is a multicentre study being conducted in 31 Low-and-Middle income countries (LMICs) across the globe. This study is being coordinated by the World Ovarian Cancer Coalition and the International Gynecologic Cancer Society

4. Rationale/Justification

Whilst the risk of developing the disease is highest in high-income countries, most women in the world with the condition live in low- or middle-income countries, and their personal risk rises as countries develop economically. As such, this new version of the Every Woman Study in low- and middle-income countries presents a major opportunity to gather novel information on patient experience, to inform and drive national and international patient and clinical advocacy efforts.

The main research question will be to identify the challenges and opportunities to improving survival and quality of life for women diagnosed with ovarian cancer in Kenya, and to provide some level of comparability amongst participating regions and countries.

5. Objectives

Broad Objective

The aim of this study is to establish the first-ever evidence base of the experiences of women with ovarian cancer living in Kenya with the purpose of identifying the key challenges and opportunities to improve survival and quality of life.

Specific objectives

1. To gather sufficient survey responses to make robust statements about the experiences of women with ovarian cancer in Kenya

2. To explore the similarities and variations in experience of women in Kenya versus the group of respondents in other participating LMIC with a view at looking for opportunities for progress

3. To explore the context in which the women are being treated, and how this varies between countries, and to showcase the findings in the context of the final report.

6. Methods

6.1 Study setting

The study will be carried out at Moi Teaching and Referral Hospital (MTRH) in Eldoret and Aga Khan Hospital in Mombasa, in Kenya. At MTRH mainly at Chandaria Cancer and Chronic Centre (CCCDC). It is the country's second largest referral hospital located in Uasin Gishu County about 300 kilometers west of the country's capital Nairobi. Being the main referral hospital in Western Kenya, it has a catchment population of 20 to 24 million people, which comprises about 40 percent of the Kenyan population and also serves parts of Eastern Uganda and Southern Sudan. It serves as a teaching hospital for Moi University, School of medicine and Kenya Medical Training College. Moi University and MTRH have two gynaecologic oncologists and five gynaecologic oncology fellow who manage the gynaecologic oncology patients. Patients will be recruited from the CCCDC clinic, on average, 100-130 patients' new cases of ovarian cancer are seen yearly in the gynaecological oncology clinic. The Aga Khan Hospital, Its satellite clinics and partner hospitals in the coastal region of Kenya, serve a catchment area of over 5 million and is only one of the two hospitals in the region with a gynecologic oncologist and a dedicated clinic for women's cancer and will enrol all-comers with a hsitological diagnosisof ovarian cancer.

6.2 Study design

This is a low risk, observational, cross-sectional study using mixed methods, primarily collection of quantitative data with some qualitative data.

6.3 Study population

Women over the age of 18 who have had a diagnosis of ovarian cancer within five years of the date of their participation in the Study will be eligible to take part as long as they are not attending on the day of diagnosis.

6.4 Eligibility Criteria

Inclusion Criteria

- Participant is willing and able to give informed consent for participation in the study.
- Female, aged between 18 and 99 years
- Diagnosed with any stage or type of ovarian, fallopian tube or primary peritoneal cancer within the previous five years (of the date of completing the survey)

- Women have already been informed of their diagnosis of ovarian cancer at a previous appointment and administrator is confident the woman understands what she has been told. If there is any doubt, the administrator must check with the clinician caring for the woman.

Exclusion Criteria

The participant may not enter the study if any of the following apply

- They are attending the hospital or clinic to receive their diagnosis of ovarian cancer.
- They are deemed too unwell to be able to cope with the demands of filling in the survey or responding to questions
- They are identified as having mental health concerns, learning difficulties, or medical conditions such as dementia, delirium, or psychosis to the extent that they would be unable to cope with the demands of filling in the survey or responding to questions, or do not understand their diagnosis
- They have already completed the survey on a previous visit to the hospital

Exclusion criteria and consent status will be recorded by the Administrator on the Redcap system, to allow for accurate calculations of response rates.

6.5 Sample size calculation

Minimum sample size have been constructed based on the five-year prevalence of ovarian cancer in Kenya, as calculated by GLOBOCAN in June 2021. The minimum sample size is based on 95% confidence +/- 10%.

The Globocan five-year prevalence of ovarian cancer in Kenya is 2,314, therefore

The calculated minimum sample size is 92.

It is hoped recruitment to the study shall continue beyond this minimum point, until June 2023, or a sample size 95% confidence +/- 5% is reached, whichever is sooner.

6.6 Sampling

Convenience sampling will be used, all consecutive women who meet the eligibility criteria and consent will be enrolled in the study until the desired sample size is reached.

6.7 Study Procedure and data collection

The PI and the research assistant (RA) will review the records of women diagnosed with ovarian cancer over the past five years, assign a Unique Identifier Number and determine eligibility using a set of questions defined, with answers recorded on the REDCAP study database (Eligibility

Criteria Instrument). This will include information regarding the diagnosis and treatment of the ovarian cancer and will be abstracted from the patients' medical charts. The RA then enters all the information but the name onto the REDCAP system. This is so that the investigators can interrogate what proportion of women were eligible to participate, and of those who were, participation rates. It also allows the investigators to compare participants v nonparticipants in terms of basic information

The RA will then give eligible women verbal and written information about the study when they attend the hospital or clinic and ask for their consent to participate. This will be done after they have seen the primary doctor, so as not to interfere with their care. Following informed consent each participant will be given the survey. This will be done in a location (room) where confidentiality will be assured. The RA will complete study procedures and collect data electronically using pretested coded questionnaires on Computers and Tablets. They can withdraw from the study at any time, either during the survey, or at any time within six months of the closure of the study.

Accordingly the survey may be completed in any of the following manners:

- Woman consents and fills in a paper survey by hand whilst attending the hospital or clinic
- A doctor, nurse or appropriate other (RA) gains consent on paper and then asks the woman the questions and records their answers either on paper, or electronically

The **quantitative data** collection will involve a 20-minute survey of women with ovarian cancer. Answers will almost exclusively be multiple choice. Just two out of 59 questions will offer free-text. Depending on their answers, women will be answering between 36 and 55 questions. Key domains include knowledge of ovarian cancer prior to diagnosis, symptoms of ovarian cancer, route and time to diagnosis, treatment choices, patient information and support needs and priorities for action.

The survey questions were from the original Every Woman Study™, an hour-long survey which was developed following qualitative work with women from a wide variety of settings including low-income settings, and subsequent thematic analysis. The original survey underwent two phases of testing with numerous women in different settings before being translated and tested once again. Ultimately the original survey attracted 1531 completions from 44 countries. The OC reviewed the processes for reducing the survey to ensure content validity is retained before translation occurs. Face validity of the English version was tested by 8 women from 4 countries (Argentina, Kazakhstan, South Africa and Vietnam). All found it an acceptable length, appropriate to their setting and experiences, and not lacking any key information. The OC approved the review process and final version in November 2021. The Kiswahili version of this survey will be tested again before data collection starts. Given the nature of the disease, and the variability of women taking up treatment due to financial and other considerations, it is appropriate ask women to submit their answers all at one time, as many may not have repeated visits to clinics.

The **qualitative elements** of the study are as follows:

Free-text answers (2) in the survey will be analysed and coded thematically by the Study Team.

The survey will be translated from English to Kiswahili and will be piloted before commencement of the study. Care will be taken to ensure that women are best placed to answer the questions without fear for their treatment, so in different settings the administrator role may be taken by different team members.

At the end of the survey women will be offered information about ovarian cancer in written form, plus contact details of Kenya Networks of Cancer Organizations (KENCO), which is a community based cancer organization active in cancer awareness that can offer support, and who have agreed to support the study.

6.8 Research Assistant

One research assistant (e.g. nurse, clinical officer, medical officer, social worker, trainee doctor or research nurse), with experience of communicating and interacting most effectively with women with ovarian cancer bearing in mind their situation will be trained on the purpose of the study, and administration of the survey to patients before the commencement of the study. They will be trained on how to upload the information on Redcap study database. The RA will be provided with the survey both in English and Swahili, as both are official languages in Kenya. The research assistants will be responsible for outlining the participant information and informed consent process, and documenting accordingly.

Patients will be allowed to answer the questionnaire in their preferred language. For patients who are not able to read in either language, the questionnaire will be administered by the RA. The RA who will be involved in consenting and enrolment of study participants will not be a member of the care team at the gynaecologic oncology or radiotherapy departments. This will ensure that there will be no perceived power imbalance by study participants.

6.9 Recruitment

Recruitment to the study will continue until the minimum sample size. Recruitment may continue beyond this point until a sample size to provide a country figure with 95% confidence +/- 5% (the ‘preferred sample size’) or the end June 2023, whichever is sooner.

6.10 Privacy and Confidentiality

The survey will be filled in a dedicated room in the CCCDC where confidentiality will be assured. I have made arrangements with the unit in-charge to gain full access of a side room at the department. This side room will only be used for the purpose of research activities associated with this study with no interruptions from facility staff. Since the door can be locked, this room provided adequate privacy for the participant being enrolled

Figure 1: Overview of Patient Recruitment and Consent

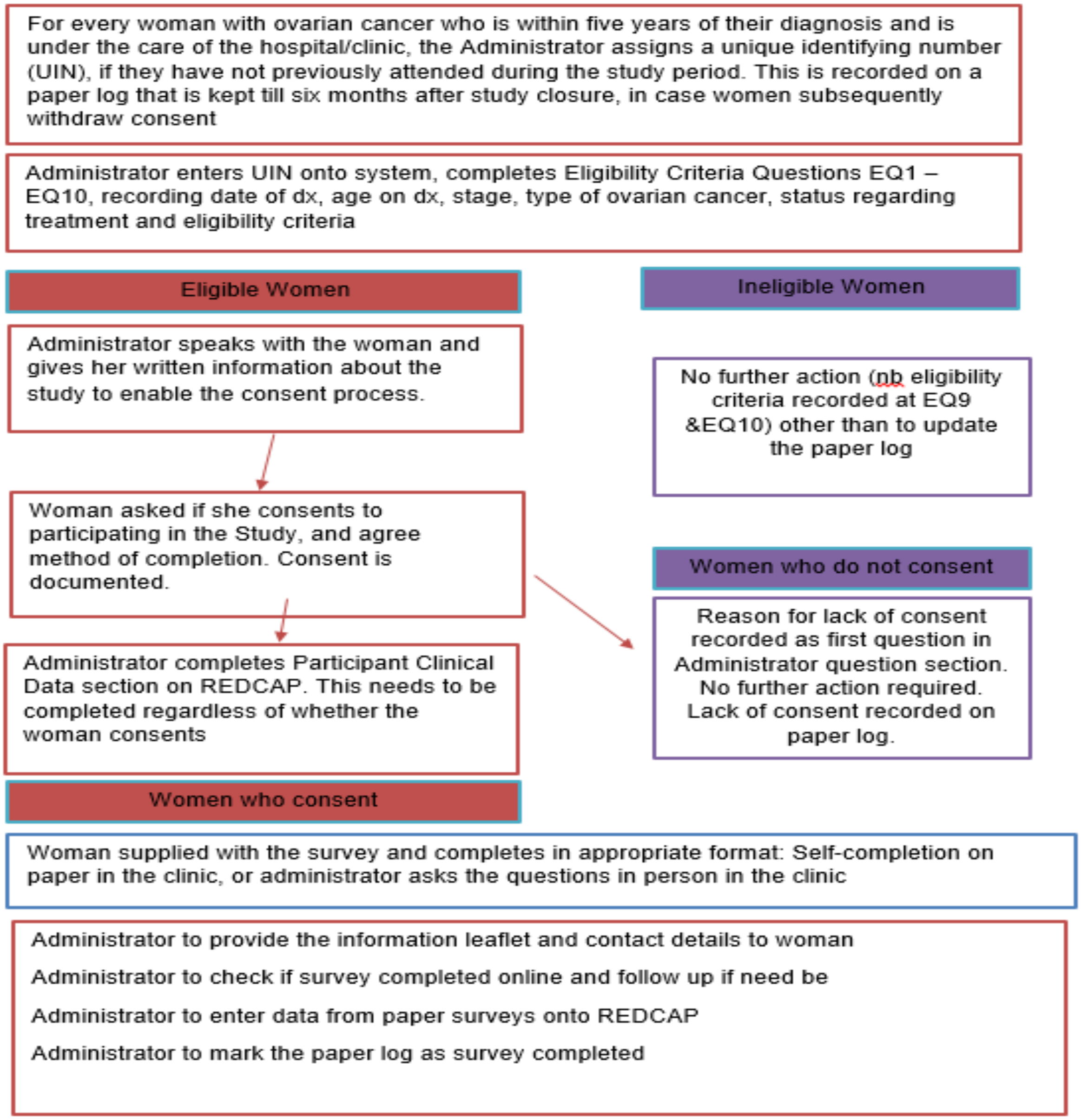

6.11 Data Management

Patient identifiable information will be destroyed by paper shredders 6 months after completion of study. As a UIN is assigned to each patient, a manual paper log is kept of the women's names and their UIN until six months after completion of the study when it will be destroyed. Data will be uploaded from any paper surveys to the survey platform, which will be based on the secure IGCS REDCAP server based in the Amazon Cloud. REDCAP is a secure web platform for building and

managing online databases and surveys. Names, email addresses and telephone numbers will not be included in the Survey Data stored on REDCAP.

Paper Log Example (to be destroyed six months after end of study)

UIN (country identifier, centre identifier, patient number	Eligibility	Participated
ABP001	No (reason recorded)	n/a
ABP002	Yes	Yes
ABP003	Yes	No (reason recorded if given)

Care will be taken at every level to maintain confidentiality and security of the database. Paper copies of competed surveys which contain patient identifiers (such as patient's hospital number) will be accessible only to the Kenyan study team and will be stored in a locked cabinet in a locked office in the CCCDC. All electronic databases used in this study will be protected by procedures consistent with applicable laws, directives, policies, regulations, and standard in Kenya. Each entry will be assigned a unique identification number and all quantitative and qualitative data collected as part of the study will be identified with this number. De-identified Data in tablets and computers will be encrypted and password protected and will be accessed by the study team members. Electronic databases with the delinked and de-identified data will be retained for future analyses and for comparison. We will follow ethical committee's guidance and approval on data collection, anonymization and the extent the data can be used beyond the immediate project.

6.12 Analysis

Statistical considerations: There will be two aspects to the statistical considerations. The global study results, and those relating only to Kenya.

The Global results will be analysed first. Categorical variables will be summarised with counts and percentages, with Chi Squared Tests or Fisher's Exact Test to compare proportions between groups. Tests such as Kruskal-Wallis Test will be used for continuous variables. Statistical significance will be set at p is less than 0.05.

The data will be exported from REDCAP to a suitable statistics programme such as SAS, Stata, R, SPSS or Microsoft Excel.

To identify statistically significant predictors of key metrics (for example time to consult a doctor, time to diagnosis), univariable and multivariable logistic regression analyses will be conducted. Multinomial logistic regression may be used to identify significant predictors, and covariates that were significant in univariable analyses will be entered into multivariable analyses.

The two free-text answers will be translated and coded into broad themes, and then if necessary into sub themes.

The initial global findings will be reviewed by the OC, to identify the key areas of focus for further statistical analysis. Key variables in the first instance will include, but not be limited to:

- Country of diagnosis and treatment
- Age on diagnosis
- Stage on diagnosis
- Type of ovarian cancer
- Education and income levels
- Time to diagnosis as a whole, and in its component parts (time to seek medical help, time from that point to diagnosis)
- Symptoms experienced
- Family history of ovarian and/or breast cancer

In relation to the Kenyan findings, the EWS Study Director will share and discuss the global findings with the PI, and where Kenya is identified as having data that is statistically significant compared to other countries. This, together with the final Kenyan sample size, will determine what further analysis is needed or desired at the Kenyan level.

Each participating country lead will receive a country report of their standalone statistics, highlighting any statistically significant variations from the average for all.

7. Assumptions and limitations

The main assumption in this study is that going via hospital and clinics will provide maximum access to women with ovarian cancer in these settings.

One key limitation may be that a significant proportion of women are likely to go undiagnosed in the community for a number of reasons due to a lack of knowledge about key symptoms, a fear of the stigma of a cancer diagnosis and inability to fund tests and potential treatments. Another limitation may be that having a health professional or other appropriate person asking the questions (administering the survey), as opposed to self-completion, women may respond differently. However, we will be able to compare answers provided directly by women to those input by study administrators. By reducing the length of the survey, and due to the different method of recruiting women, only very broad comparisons may be made to the original study, but the main purpose is to provide the first ever patient experience evidence base in these settings, and to provide some level of comparability to other participating countries, and a baseline for monitoring progress.

Recruiting lead centres in countries that have connections to the International Gynecologic Cancer Society or the World Ovarian Cancer Coalition will mean that by default there will be some focus

on gynaecologic oncology in that setting. This may mean that the results do not reflect fully the experiences of women living in countries with no such connections, and may be more favourable.

8. Ethical and Regulatory Considerations

There are several ethical considerations that have been accounted for in the study:

- Women will not be asked to complete the survey on the day they are given their diagnosis
- There is a small chance that women may become upset when reflecting on their experiences. Administrators will be on hand to support them, and women will be given information and contact numbers for local or national groups that can support them. This may include a social worker, nurse, support group or non-governmental organisation (NGO)
- Having asked women about their information needs, it is only right that participants are then given some information in their local language about ovarian cancer and how it is treated, and details of how to contact NGOs who may be able to help. The study team will work with national or local groups in each country to identify and collaborate with local NGOs on this matter. In this instance KENCO have agreed to provide support on this matter
- Administrators will need to be mindful that ideally women should be able to complete the survey by themselves, and not in the presence of a family member, so that women can share their views openly.

8.1 Informed consent

Written and verbal versions of the Participant Information and Informed Consent will be presented to the participants detailing no less than: the exact nature of the study; what it will involve for the participant; the implications and constraints of the protocol; any risks involved in taking part. It will be clearly stated that the participant is free to withdraw from the study at any time for any reason without prejudice to future care, and with no obligation to give the reason for withdrawal. Information on how they can with draw will be included.

The participant will be allowed as much time as wished to consider the information, and the opportunity to question the administrator, to decide whether they will participate in the study. Written Informed Consent will then be obtained by means of participant dated signature and dated signature of the administrator who presented and obtained the Informed Consent. A copy of the signed Informed Consent will be given to the participant. The original signed form will be retained at the study site. The Patient Study Information and Consent Form can be found in Appendix II.

8.2 Institutional Review Board

The investigator will obtain regulatory approval from the Institutional Research and Ethics Committee (IREC) at Moi Teaching and Referral Hospital and the Aga Khan University Hospital

as well as Research permit for the National Commission for Science, Technology and Innovation (NACOSTI) in Kenya and no data collection will take place prior to approval. All correspondence with the IREC will be retained in the regulatory or trial master file. Copies of IREC approvals will be filed with other study documents.

8.3 Reporting

An End of Study notification and final report will be submitted to the IREC as required, with an interim report six months from the start of data collection, if requested.

8.4 Potential Risks and Benefits

This study carries a minimal risk to participants. However, there is some risk that confidential information can be disclosed. To mitigate this, all study personnel will undergo special training on research ethics and confidentiality. We will ensure that the participants' anonymity is maintained. The participants will be identified only by a Unique Identifier Number (UIN) on all trial documents and any electronic database, with the exception of a central separate eligibility and participation log, where a participant's name will be recorded. All documents will be stored securely and only accessible by study staff and authorised personnel, and will only be retained until 6 months after the study end, until analysis is complete in case consent is withdrawn by a participant. At this point all physical paper work will be destroyed.

Participants will not receive any payments, benefits or expenses relating to this study. However findings of the study may help the hospital improve its care in the future and may impact on policy changes benefitting women with ovarian cancer in Kenya.

8.5 Discontinuation/Withdrawal of Participants from Study

Each participant has the right to withdraw from the study up until 31st June 2023, by informing a member of the study team. In addition, the Investigator may discontinue a participant from the study at any time if the Investigator considers it necessary for any reason including:

- Ineligibility (either arising during the study or retrospectively having been overlooked at screening)
- Significant protocol deviation
- Significant non-compliance with study requirements

Withdrawal from the study will result in the exclusion of the data for that participant from the analysis. This will be done at the local site, as the Principal Investigator and Country Lead Clinician will only see anonymised data. The participant may or may not be replaced by another participant, dependent on whether or not the minimum sample sizes for the country have been met.

The reason for the withdrawal of consent will be logged by the administrator. Participants can choose not to give a reason for withdrawal of consent.

9. Definition of End of Study

The earliest end of the study is the date at which minimum participation rates have been achieved, and as a maximum 31st June 2023. Centres within a country may choose to recruit more participants than the minimum sample size in order to improve data analysis. The CL, in conjunction with the OC will determine that final level and agree the closing point accordingly

10. Funding

Funding for the study is provided by the Sponsors from their core funds. Both are not for profit organisations. This covers all costs relating to translation and ethics submission if pre-agreed, by the sponsors, and on submission of appropriate paperwork. The Sponsors will also support the cost of a RA to cover the one clinic each week at which women with ovarian cancer will be attending. Direct funding by industry sponsors is not being used in this study.

11. Dissemination of Research Findings

Findings from this research work will published in peer-reviewed journals and presented in local and international scientific meetings and/or conferences

12. References

Cheserem, E. J. *et al.* (2013) 'Ovarian cancer in Kenyatta National Hospital in Kenya: Characteristics and management', *Open Journal of Obstetrics and Gynecology*, 03(01), pp. 165–171. doi: 10.4236/ojog.2013.31a031.

Kenya, Globocan (2020) *the Global Cancer Observatory*.

[1] https://ijgc.bmj.com/content/31/2/238
[2] https://gco.iarc.fr/tomorrow/en (Accessed June 21st 2021)

13. Time Frame

Timeline is divided in three monthly segments

Time frame	2022			2023			
Activity	June	September	December	March	June	September	December
IREC Review							
Training of RA							
Data collection							
Quarterly review							

Data analysis							
Dissemination of findings							

14. Budget

Personnel	Unit/pax	Monthly pay	# of months	Total cost
**Position**				
Research assistant	1	$150	12	$1800
**Subtotal personnel**				**$1800**
Equipment				
**Description**	**Unit type**	**# units**	**Unit cost**	**Total coast**
Storage cabinet		1	$150	$150
Laptop		1	$700	$700
Supplies	printing		$200	$200
Internet/stationary			$150	$150
**Subtotal equipment**				**$1200**
Other direct costs				
**Description**	**Unit type**	**#units**	**Unit cost**	**Total cost**
Ethical review cost			$500	$100
NACOSTI cost			$100	$100
Translation cost			$680	$680
**Subtotal other direct cost**				**$880**
Honorarium to MTRH				**$500**
				**TOTAL BUDGET: $4380**

15. Signatures of Principal Investigators

Afrin Fatima Shaffi

[signature]

14.06.2022

Frances Reid

[signature]

13th June 2022

16. Appendices
16.1. Appendix I

The Every Woman Study LMIC Edition – Eligibility Criteria and basic data

**Questions filled in by the administrator**	THIS INFORMATION SHOULD BE COMPLETED FOR ALL WOMEN AS INSTRUCTED, WHETHER OR NOT THE WOMAN CONSENTS TO TAKE PART.<br><br>This will allow us to analyse key factors about those who decline to participate.
**Unique Identifying Number**	_______________
EQ1	Is the patient normally resident in this country?<br><br>Yes, and was diagnosed here<br>Yes, but was diagnosed in another country (enter country of diagnosis)<br>No, but was diagnosed here<br>No, was diagnosed elsewhere and has travelled for treatment (enter country of diagnosis)<br><br>In what country was the patient diagnosed? …………………………………………………
EQ2	When was the patient diagnosed with ovarian cancer?<br>Diagnosis Month…………………..<br>Diagnosis Year……….
EQ3	What age was the patient when she was diagnosed with ovarian cancer?<br><br>(Number)
EQ4	What type of ovarian cancer was the patient diagnosed with?<br><br>Epithelial ovarian cancer (unspecified)<br>Epithelial (clear cell) ovarian cancer<br>Epithelial (high-grade serous) ovarian cancer<br>Epithelial (low-grade serous) ovarian cancer<br>Epithelial (endometrioid) ovarian cancer<br>Epithelial (mucinous) ovarian cancer<br>Germ cell ovarian cancer<br>Sex cord stromal ovarian cancer<br>Primary peritoneal ovarian cancer<br>Fallopian tube cancer

	Granulosa tumour of the ovary<br>Borderline tumour<br>Teratoma of the ovary<br><br>If the patient has another type of ovarian cancer, or an additional type of ovarian cancer please enter it here.<br>…………………………………………………
EQ5	What form of staging of the patient's cancer has taken place?<br>Surgical or pathological staging<br>Clinical staging (based on physical exam, biopsy and imaging)<br>Clinical opinion only<br>I do not know
EQ6	What stage was the ovarian cancer diagnosed at? Select the answer which best describes the stage.<br><br>FIGO stage I (T1, N0, M0) Tumor limited to the ovaries (one or both)<br>FIGO stage IA (T1a, N0, M0)<br>FIGO stage IB (T1b, N0, M0)<br>FIGO stage IC (T1c, N0, M0)<br><br>FIGO stage II (T2, N0, M0) Tumor involves one or both ovaries with pelvic extension below pelvic brim<br>FIGO stage IIA (T2a, N0, M0)<br>FIGO stage IIB (T2b, N0, M0)<br><br>FIGO stage III Tumor involves one or both ovaries with microscopically confirmed peritoneal metastasis outside the pelvis and/or retroperitoneal lymph node involvement<br>FIGO stage IIIA (T1 or T2, N1, M0) or (T3a, N0 or N1, M0)<br>FIGO stage IIIB (T3b, N0 or N1, M0)<br>FIGO stage IIIC (T3c, N0 or N1, M0)<br><br>Figo Stage IV Distant metastasis including cytology-positive pleural effusion; liver or splenic parenchymal involvement; extra-abdominal organ involvement including inguinal lymph nodes; transmural intestinal involvement<br>FIGO Stage IVA (Any T, Any N, M1a)<br>FIGO Stage IVB (Any T Any N, M1b)<br><br>I do not know
EQ7	Is the patient currently?<br><br>Undergoing or recovering from surgery

	Undergoing treatment for newly diagnosed ovarian cancer<br>Undergoing treatment for recurrent ovarian cancer<br>In remission<br>Receiving palliative care<br>Choosing not to receive treatment<br>Other
EQ8	Has the patient's ovarian cancer ever returned?<br>Yes<br>No<br>It never went away<br><br>If the cancer returned, please enter the date it FIRST returned<br><br>Recurrence month……..<br>Recurrence year………….
**Inclusion Criteria**<br><br>**EQ9**	Does the patient meet the inclusion criteria?<br>Tick all that apply<br>• The patient can give informed consent for participation in the study.<br>• The patient is biologically female, aged between 18 and 99 years<br>• The patient has been diagnosed with ovarian, fallopian tube or primary peritoneal cancer within the previous five years (of the date of completing the survey)<br>• The patient has already been informed of their diagnosis of ovarian cancer at a previous appointment and understands the diagnosis<br><br>ALL ANSWERS NEED TO BE SELECTED IN ORDER TO PROCEED
**Exclusion criteria**<br><br>**EQ10**	Does the patient meet any of the following exclusion criteria?<br>Tick all that apply<br>• The patient is attending the hospital or clinic to receive their diagnosis of ovarian cancer.<br>• The patient is deemed too unwell to be able to cope with the demands of filling in the survey or responding to questions<br>• The patient is identified as having mental health concerns, learning difficulties, or medical conditions such as dementia, delirium, or psychosis to the extent that they would be unable to cope with the demands of filling in the survey or responding to questions<br>• The patient has already completed the survey on a previous visit to the hospital<br>• The patient does not meet any of the exclusion criteria.<br><br>IF ANY OF THE EXCLUSION CRITERIA ARE MET, THE PATIENT WILL BE EXCLUDED FROM THE STUDY AT THIS POINT. PLEASE RECORD THE RESULTS ON REDCap AND THE PAPER LOG.

	IF THE PATIENT IS ELIGIBLE TO PARTICIPATE PLEASE COMPLETE THE INFORMATION BELOW.

AQ2	Which tests were undertaken at any point, to decide if the patient had ovarian cancer? TICK ALL THAT APPLY<br><br>Clinical examination<br>CA125 blood test<br>Abdominal Ultrasound<br>Transvaginal ultrasound<br>MRI scan<br>CT scan<br>X-ray<br>Other<br>I do not know
AQ3	Has the patient ever had surgery to treat or control their ovarian cancer?<br><br>No (GO TO AQ5)<br><br>Yes, with complete tumour cytoreduction to no gross residual disease and all macroscopic residual disease removed. *This definition is in line with the ASCO Stratified Guidelines for Ovarian Cancer, Vanderpuye et al 2021*<br><br>Yes, but not with complete tumour cytoreduction to no gross residual disease and all macroscopic residual disease removed
AQ4	Has the patient ever had any of the following? Tick all that apply<br><br>Chemotherapy before first surgery (Neo-adjuvant chemotherapy)<br>A second operation because the first operation did not remove enough of the cancer<br>A second operation for a recurrence of ovarian cancer
AQ5	Has the patient ever had chemotherapy to treat or control their ovarian cancer?<br><br>Yes<br>No<br>(IF NO, Go to AQ7)
AQ6	Which chemotherapy drugs has the patient been treated with at any point since their diagnosis with ovarian cancer. Tick all that apply (list)<br><br>Carboplatin<br>Cisplatin<br>Paclitaxel (Taxol)

	Pegylated Liposomal Doxorubicin Hydrochloride (Caelyx,Myocet,Doxil)<br>Gemcitabine<br>Trabectedin<br>Topotecan<br>Etoposide<br>Cyclophosphamide<br>Bleomycin<br>Other
AQ7	Has the patient ever been tested to find any genetic mutations?<br><br>Yes, pre-diagnosis<br>Yes, post-diagnosis<br>No, they have not but it is available in this country<br>No, it is not available in this country<br>IF NO GO TO AQ9
AQ8 if answered yes to AQ7	Which genetic mutation was found?<br><br>BRCA1<br>BRCA2<br>Lynch Syndrome<br>Other<br>No genetic mutation was found
AQ9	Has the patient ever received one or more of the following hormone treatments? Tick all that apply<br>Tamoxifen<br>Anastrozole<br>Letrozole<br>These drugs are not available
AQ10	Are any of the following treatments available in your country, either routinely or occasionally? TICK ALL THAT APPLY<br><br>Intraperitoneal chemotherapy<br>Hyperthermic chemotherapy<br>Bevacizumab<br>Olaparib<br>Rucaparib<br>Niraparib<br>None of these treatments are available
AQ11	If any of the above treatments were selected, has this patient ever received any of them as part of her treatment in your country? TICK ALL THAT APPLY<br><br>Intraperitoneal chemotherapy<br>Hyperthermic chemotherapy

	Bevacizumab<br>Olaparib<br>Rucaparib<br>Niraparib<br>I don’t know

16.2. Appendix II: Patient Study Information and Consent Form (English version)

Available on paper, or online at the beginning of the Survey. Note the content of the survey is only accessible beyond this point if the patient consents.

This hospital is participating in a research study called **The Every Woman Study™** in low- and middle-income countries, and we would like to invite you to participate. The aim of the study is to identify the challenges and opportunities to improve survival for women with ovarian cancer. It does not involve you trying new medicines or procedures but is a survey that will take at least 20 minutes to complete. It will ask about any symptoms you experienced, how you were diagnosed, your needs since diagnosis, and where you would like to see improvements made in the diagnosis and care of women with ovarian cancer.

You do not have to take part in the study. It is entirely voluntary, so you can choose not to take part. The care you receive here will not be affected in any way. If you decide to take part and then change your mind, that is also fine.

Your answers will be joining those of over two thousand women from 31 countries around the world, and together, they will help prioritise improvements to the diagnosis and care of women with ovarian cancer at this hospital, in this country, and more widely in other low- and middle-income countries.

This Study is supported and funded by the World Ovarian Cancer Coalition and the International Gynecologic Cancer Society. These two global non-profits are strategic advocacy partners who together have hundreds of members, patient survivors, and partner organizations around the world. Through their own work and with this joint Study, both organizations are committed to ensuring that women who have ovarian cancer get the best possible care, no matter where they live.

Risks and benefits of taking part

There is no direct benefit to you in taking part in the study. However, many women find it beneficial to share their experiences with others and the findings of the study may help this hospital improve its care in the future and may impact on policy changes benefitting women with ovarian cancer in your country.

Occasionally some women may find it upsetting to think about their experiences, but please be reassured that the doctors and nurses will help where they can and will provide you with details of people or organisations you can contact if you need further support. You do not have to provide an answer to every question, but we hope you will want to provide as many answers as possible.

You may be asked to fill in the survey either online, or on paper in the hospital or clinic setting. Alternatively, you may be given the option of filling in the survey online from home, or of having someone ask you the questions, and that person making a record of your answers.

Whilst we will have to retain a separate paper record of your name and unique identifier number, your name will not be uploaded to the study electronic database with any of your answers. If you complete the survey via an online link sent to your email address, your email address will be stored on the study database, but not included in the data that is analysed. This means you will not be identifiable from your answers. In June 2023 we will destroy any physical information that links your name to your study number. If you change your mind about taking part in the study, you can withdraw your consent by telling a member of your hospital team. They will use your unique identifier number to extract your answers from the study. You can do this at any point up to 31st December 2022.

Please enter your Unique Identifier Number (UIN)………………………..

Please select only ONE of the following statements

- I have understood the purpose of this study and my role in it. I give my consent that the researchers may use my answers (not linked to my name) as part of the study.
- I have understood the purpose of this study and my role in it. I consent to the presence of the survey administrator for the purpose of facilitating my participation in the study
- I still have some questions I would like answered before I decide
- I have understood the purpose of this study, and what you are asking me to do. However, I do not consent to taking part. If you would like to share the reason for this, please do so here.

Patient signature and date *(for paper copy only)*

Administrator signature and date *(for paper copy only)*

16.3. Appendix III: Maelezo na Ridhaa ya kushiriki katika utafiti. (Kiswahili version)

Hospitali hii inashiriki katika utafiti unaoitwa **The Every Woman Study™** katika nchi za mapato ya chini na kati na tungependa kukualika kushiriki. Lengo la somo hili ni kutambua changamoto na fursa za kuboresha kuendelea kwa kuishi kwa wanawake wenye saratani ya nyumba ya mayai. Utafiti huu haukuhusishi kutaka kujaribu dawa ama taratibu mpya lakini ni utafiti utakaochukua madakika 20 kumaliza. Utauliza juu ya dalili zozote unaeza kua umepata, jinsi ulivyogunduliwa, mahitaji yako toka utambuzi na ni wapi ungetaka kuona maendeleo kwa utambuzi na utunzaji wa wanawake wenye saratani ya nyumba ya mayai.
Sio lazima ushiriki katika utafiti huu. Ni kwa hiari yako kabisa, Kwa hivyo una uhuru kukataa kutoshiriki. Utunzaji utakaopata hapa hautaadhiriwa kwa njia yoyote. Iwapo utaamua kushiriki, kisha ubadilishe mawazo ya kushiriki, ni sawa pia.

Majibu yako yatawekwa pamoja na wanawake wengine zaidi ya elfu mbili kutoka nchi 31 kote duniani, na yatasaidia kuweka kipaumbele maboresho kwa utambuzi na utunzaji wa wanawake wenye saratani ya nyumba ya mayai kwenye hospitali hii, nchi hii na kwa upana katika nchi za mapato ya chini na kati.

Utafiti huu umeungwa mkono na kufadhiliwa na Muungano wa saratani ya nyumba ya mayai na jumuiya ya saratani ya magonjwa ya wanawake kimataifa. Mashirika haya mawili yasiyo ya faida ulimwenguni ni washirika wa utetezi wa kimkakati na wana mamia ya wanachama, wagonjwa walionusurika, na mashirika ya kimarafiki kote duniani. Kupitia kwa kazi yao na utafiti huu wa pamoja, mashirika yote mawili yamejitolea kuhakiksha ya kwamba wanawake wenye saratani ya nyumba ya mayai wanapata utunzaji bora zaidi, bila kujali wanapoishi.

Hatari na faida za kushiriki
Hakuna faida ya moja kwa moja unayopata ukishiriki katika utafiti huu. Dhili ya uzoefu, wanawake wengi wanaona kuna faida kupeana maono yao kwa wengine. Matokeo ya utafiti huu yataweza kusaidia hospitali hii kuboresha utunzaji wake katika siku zijazo na yaweze kuathiri mabadiliko ya sera kufaidisha wanawake wenye saratani ya nyumba ya mayai katika nchi yao.

Mara kwa mara wanawake wataeza kasirika wakifikiria juu ya uzoefu wao, lakini kua na uhakika ya kwamba madaktari na wauguzi watasaidia wawezapo na watawapea maelezo ya watu na mashirika unaweza kuwasiliana nao. Sio lazima upeane jibu kwa kila swali, lakini tuna imani utapeana majibu mengi iwezekanavyo. Waweza kuulizwa kujaza utafiti huu mtandaoni, au kwa karatasi hospitalini au kliniki. Pia waweza pewa chaguo la kujaza utafiti kwenye mtandao ukiwa nyumbani au mtu kukuuliza maswali hayo na kurekodi majibu yako.

Tutahitaji kuhifadhi rekodi yako katika karatasi tofauti ya jina lako na nambari ya kitambulisho cha kipekee, lakini jina lako halitawekwa kwenye maelezo ya kielektroniki ya utafiti huu na majibu yako yoyote. Hii yamaanisha ya kwamba hutaweza kutambulika kutoka majibu yako. Katika mwezi wa Juni mwaka wa 2023 tutaharibu maelezo yoyote yanayounganisha jina lako kwa nambari ya utafiti. Ukibadilisha mawazo yako kushiriki katika utafiti huu, waweza kutoa ridhaa yako ya kushiriki katika utafiti kwa kumwambia mwanachama wa timu ya hospitali yako. Watatumia nambari ya kitambulisho cha kipekee kutoa majibu yako kwenye utafiti. Waweza fanya hivi kwa wakati wowote hadi 31st Desemba 2022.

Tafadhali weka nambari yako ya kitambulisho cha kipekee……………………….. *(nakala ya karatasi)*

Tafadhali chagua MOJA kwa sentensi zifuatazo

- Nimeelewa kusudi wa utafiti huu na jukumu langu kwayo. Napeana idhini yangu kwa watafiti watumie majibu yangu (yasiyounganishwa na jina langu) kwa sehemu ya utafiti.

- Nimeelewa kusudi wa utafiti huu na jukumu langu kwake. Napeana idhini yangu kwa uwepo wa msimamizi wa utafiti kwa ajili ya kuwezesha ushiriki wangu kwa utafiti.

- Bado nina maswali kuhusu utafiti huu ambayo ningetaka yajibiwe kabla uamuzi.
- Nimeelewa kusudi wa utafiti huu, na vile mngetaka nifanye. Hata hivyo, sikubali kushiriki.

  Kama ungependa kueleza sababu, tafadhali fanya hivyo hapa.

Sahihi ya mgonjwa na tarehe (nakala ya karatasi)

Sahihi ya msimamizi na tarehe *(nakala ya karatasi)*

16.4. Appendix IV: Every Woman Survey (English version)

UNIQUE IDENTIFIER NUMBER (UIN)…………………………………………………………

About You	
Q1	Are you filling in this questionnaire about your own experience of ovarian cancer?<br><br>Yes – **GO TO Q2**<br>No – **GO TO Q1A**
Q1a (if answered No to Q1)	If you are filling in this questionnaire with someone who has ovarian cancer, what is your role?<br><br>Doctor/Trainee Doctor/Nurse/Trainee Nurse/Researcher/Social Worker/Other (please specify)

	The questions that follow are those that you should ask the woman with ovarian cancer about her experience. Other than a simple explanation, please ensure you do not suggest which answers they should give.
Q2	Please indicate your ethnicity. By ethnicity we mean your feeling of belonging and attachment to a distinct group of a larger population that shares their ancestry, colour, language, or religion. You may select more than one option<br><br>***Each Country Lead Clinician will be asked to supply a standard but useful list from their own country, and include /Mixed/Other/I'd rather not say. Please use the agreed terms here. No need to translate this instruction. Just the question and answers***
Q3	On average, how long does it take you to travel to the hospital where you are seen in connection to your ovarian cancer?<br><br>Under 15 minutes<br>15-30 minutes<br>30-60 minutes<br>1-2 hours<br>2-5 hours<br>Over 5 hours<br>Over 24 hours
Q4	What is the **highest** level of education you have achieved?<br><br>***(Local teams to select most suitable descriptors – using ISCED levels (International Standard Classification of Education 2011) Please use the agreed levels and get these translated, rather than exact translation of the terms below. If in doubt contact ews@worldovariancancercoalition.org. No need to translate this instruction. Just the question and answers.***<br><br>No formal education<br>Primary education (childhood, elementary, early years, ISCED level 1)<br>Secondary education (middle school, high school, ISCED levels2,3)<br>Post-secondary, non-tertiary education (technical college, community college, ISCED levels 4, 5)<br>Tertiary education (university or similar professional skills, ISCED level 6)<br>Post-graduate education (University Master's or Doctorate or senior professional qualification, ISCED levels 7 or 8)
Q5	Just before you were diagnosed with ovarian cancer, were you?<br><br>Married or living with a partner<br>Widowed<br>Divorced or Separated<br>In a relationship but not living together<br>Single (never married)<br>Prefer not to say

Q6	Just before you were diagnosed, in your view what was your household income?<br><br>Below average for your country<br>Average for your country<br>Above average for your country<br>Prefer not to say
Q7	Around the time of your diagnosis, did you have any caring responsibilities for others in your family? **TICK ALL THAT APPLY**<br><br>Yes, a child or children under the age of 18<br>Yes, an elderly relative<br>Yes, a sick partner<br>Other<br>No, I have not had caring responsibilities
Q8	Just before you were diagnosed with ovarian cancer, were you?<br><br>In paid full-time employment<br>In part-time employment<br>Self-employed<br>Not in paid or self-employment<br>Retired<br>Other
Q9	How have you paid for your medical care/ovarian cancer care up to this point? **TICK ALL THAT APPLY**<br><br>Private Medical Insurance<br>State Medical Insurance<br>I have paid<br>Family members have paid<br>I have raised money to cover costs by crowdfunding<br>I have sought financial help from a Non-profit/Non-Governmental Organisation/Charity<br>Treatments for ovarian cancer are free<br>Diagnostic tests for ovarian cancer are free
Q10	In relation to your gynaecological history before you were diagnosed with ovarian cancer, **TICK ALL THAT APPLY**<br><br>I had entered the menopause where I no longer have monthly periods<br>I have never given birth to a child<br>I have given birth once<br>I have given birth twice

	I have given birth three or more times<br>I breast fed my children<br>I used the oral contraceptive pill for up to 5 years<br>I used the oral contraceptive pill for between 5 and 10 years<br>I received treatment for infertility

Family History	
Q11	Have any of the following family relatives (i.e. blood relatives on either your mother or your father's side of the family) had ovarian cancer?<br>**TICK ALL THAT APPLY**<br><br>Mother<br>Daughter<br>Sister<br>Aunt<br>Cousin<br>Grandmother (mother's side)<br>Grandmother (father's side)<br>Other more distant relatives (mother's side)<br>Other more distant relatives (father's side)<br>No, none of my close family have been affected<br>I do not know or cannot remember
Q12	Have any of the following family relatives (i.e. blood relatives on either your mother or your father's side of the family) had breast cancer?<br>**TICK ALL THAT APPLY**<br>Mother<br>Father<br>Daughter<br>Son<br>Sister<br>Brother<br>Aunt (either side)<br>Uncle (either side)<br>Cousin (either side)<br>Grandmother (either side)<br>Grandfather (either side)<br>No, none of my close family have been affected<br>I do not know or cannot remember
Q13	Are you aware that having blood relatives with ovarian, breast, pancreatic, prostate, bowel or womb cancer might increase the risk of a woman developing ovarian cancer?

	Yes<br>No<br>Not Sure

**Leading up to diagnosis**	
Q14	Before you were diagnosed with ovarian cancer, how much if anything did you know about ovarian cancer?<br><br>I had heard of it and knew something about it<br>I had heard of it but did not know anything about it<br>I had never heard of it<br>I do not know or cannot remember
Q15	Before your diagnosis of ovarian cancer, which, if any, of the following symptoms did you experience?<br>**TICK ALL THAT APPLY**<br>Pain in the abdomen (stomach)<br>Urinary frequency<br>Difficulty eating<br>Changes in bowel habit (e.g., diarrhoea or constipation)<br>Extreme fatigue<br>Increased abdominal size<br>Feeling full<br>Unexplained weight loss<br>Urinary urgency<br>Pain in the pelvis<br>Persistent bloating<br>Other symptoms<br>**(IF YOU SELECTED ANY OF THE ABOVE OPTIONS GO TO Q17)**<br>None of the above **(GO TO Q16)**
Q16 only if they answer None of the Above in Q15	As you **did not** experience symptoms outlined in the previous question before you were diagnosed, what led to your diagnosis?<br>**TICK ALL THAT APPLY**<br><br>A routine examination<br>A routine scan<br>A routine blood test<br>The cancer was discovered whilst I was being treated for something else<br>Other<br>**NOW GO TO Q27**
Q17 for women who had symptoms	When did you first notice any of the symptoms selected above?<br>Please enter the month, and the year.<br>If you do not remember, please leave this blank.

listed in Q15	…………………... Month symptoms began<br>………………….. Year symptoms began
Q18	How concerned about your symptoms were you before your diagnosis?<br><br>Concerned<br>Somewhat concerned<br>Not very concerned<br>Not at all concerned<br>I do not know or cannot remember
Q19	Did you seek advice from someone about your symptoms, for example a doctor, nurse, pharmacist or healer?<br><br>Yes **(GO TO Q21)**<br>No **(GO TO Q20)**
Q20<br>If they answer no to Q19	As you did not seek advice from someone about your symptoms, what led to your diagnosis?<br>**TICK ALL THAT APPLY**<br><br>A routine examination<br>A routine scan<br>A routine blood test<br>The cancer was discovered whilst I was being treated for something else<br>Other<br>**(GO TO Q26)**
Q21	Which type of person, other than a family member, did you **first** seek advice from, about your symptoms?<br>**SELECT ONLY ONE ANSWER**<br><br>A local healer<br>An alternative health practitioner<br>A family doctor<br>A gynaecologist<br>A gynaecologic oncologist (a doctor specialising in the treatment of ovarian cancer)<br>A gastroenterologist<br>An emergency room or accident and emergency doctor<br>A nurse<br>A pharmacist<br>Someone else

Q22	In addition to the first person, you sought advice from about symptoms, who else did you visit or talk to about your symptoms in the time before you were diagnosed?<br>**TICK ALL THAT APPLY**<br><br>A local healer<br>An alternative health practitioner<br>A family doctor<br>A gynaecologist<br>A gynaecologic oncologist (a doctor specialising in the treatment of ovarian cancer)<br>A gastroenterologist<br>An emergency room or accident and emergency doctor<br>A nurse<br>A pharmacist<br>A family member<br>Someone else<br>No one else
Q23	When did you first visit **a medical doctor** about your symptoms?<br><br>Please enter the month, and the year.<br>If you do not remember, please leave this blank.<br><br>………………….. Month visited Medical Doctor for Symptoms<br>………………….. Year visited Medical Doctor for Symptoms
Q24	In your view, how seriously did the medical doctor you first saw take your concerns about symptoms?<br><br>Very seriously<br>Fairly seriously<br>Not very seriously<br>Not at all seriously<br>I do not know or cannot remember
Q25	Approximately how many times did you talk or visit medical doctors (any type of doctor, not just the one you saw first) before you were told you had ovarian cancer?<br><br>If you cannot remember, please leave this blank<br><br>___ (Enter a number)

Q26	Do you feel that the time from you first experiencing symptoms to being diagnosed with ovarian cancer could have been shortened?<br><br>Yes<br>No<br>Not sure<br><br>Please use the COMMENT box to let us know how you think the time could have been shortened (for example doctor delay, health system delay such as having to wait for tests or appointments) or factors that may have delayed you seeking help.<br><br>COMMENTS
Q27	When were you told by a medical doctor that you had ovarian cancer?<br><br>Please enter the month, and the year.<br>If you do not remember, please leave this blank.<br><br>………………….... Month told by a medical doctor you had ovarian cancer<br>………………….. Year told by a medical doctor you had ovarian cancer

Treatments for ovarian cancer	
Q28	When did you first begin any treatment for ovarian cancer, such as surgery or chemotherapy?<br><br>If you cannot remember, or have not had any treatment please leave this blank<br><br>………………….Month treatment began<br>………………….Year treatment began

Q29	In deciding what, if any, treatments you will have to control your ovarian cancer or deal with side effects from treatment, which of the following will affect your decision?<br>**TICK ALL THAT APPLY**<br><br>The opinion of the doctor<br>The opinion of my family<br>I will make up my own mind<br>The cost of treatment drugs<br>Other costs associated with treatment such as transport or accommodation<br>The chance to cure or extend my life<br>The side effects of treatment<br>**(IF YOU HAVE SELECTED ANY OF THE OPTIONS ABOVE PLEASE CONTINUE TO Q 30)**<br>None of the above **(GO TO Q31)**
Q30	Which of the statements you selected in the previous question is **most** important to your decision making?<br>**SELECT ONLY ONE ANSWER**<br><br>The opinion of the doctor<br>The opinion of my family<br>I will make up my own mind<br>The cost of treatment drugs<br>Other costs associated with treatment such as transport or accommodation<br>The chance to cure or extend my life<br>The side effects of treatment
Q31	Overall, do you feel as involved as you would like to be, in decisions about your treatment throughout your cancer care, such as deciding which treatments, or whether to have any treatment?<br><br>Yes always<br>Yes most of the time<br>Only some of the time<br>Not at all<br>I do not know or cannot remember
Q32	Which, if any, of the following side effects of treatment for ovarian cancer have been **most** difficult to deal with at any point since you started treatment?<br>**SELECT UP TO TWO ANSWERS**<br><br>I have not had any treatment (GO TO Q35)<br>Tiredness or fatigue<br>Anxiety<br>Feeling or being sick (nausea or vomiting)<br>Diarrhoea

	Loss of appetite<br>Inability to fight infection<br>Hair thinning or hair loss<br>Dry skin<br>Sore mouth<br>Tingling or numbness in hands or feet<br>Sleep loss<br>Metal taste in mouth<br>Constipation<br>Allergic reaction<br>Joint aches or pains<br>Swelling<br>Muscle aches or pains<br>Menopausal symptom<br>Complications following surgery<br>Another side effect<br>**(IF YOU HAVE SELECTED ANY OF THE ABOVE GO TO Q33)**<br>No side effects in particular **(GO TO Q35)**
Q33 if they had side effects	Would you describe these most difficult side effects as 'long-term', in other words, persisting for some weeks or months after treatment has ended?<br><br>Yes<br>No<br>I am still in treatment so cannot say if they are long term<br>I am not sure
Q34	Have health professionals been able to help reduce the impact of side effects with other medicines, or advice and information?<br><br>Yes very much so<br>Yes to some extent<br>Not at all<br>I did not seek help, or I did not need to seek help about side effects<br>I do not know or cannot remember
Q35	Have you at any time since your diagnosis, used herbal, complementary, or alternative treatments not prescribed by your doctor at the hospital, to try and control your ovarian cancer, or any pain caused by your ovarian cancer?<br><br>Yes all the time<br>Yes at some point<br>No, but I have thought about it<br>No, not at all<br>I do not know

Q36	Have you ever wanted to get another opinion from a second doctor about your ovarian cancer and treatment?<br><br>No<br>Yes and I have been able to<br>Yes but I don't know who to ask<br>I have not thought about it

**Emotional Support needs**	
Q37	Up to now, have there been times when **you** have felt in need of emotional support?<br>**TICK ALL THAT APPLY**<br><br>At the time of diagnosis<br>During treatment<br>After treatment ended<br>When the cancer returned<br>When told the cancer was not curable<br>Other<br>**(IF YOU HAVE SELECTED ANY OF THE ABOVE GO TO Q38)**<br>I have not needed any emotional support **(GO TO Q 42)**
Q38<br>If they have needed emotional support	Are there particular issues you have faced?<br>**TICK ALL THAT APPLY**<br><br>Fear of the cancer returning<br>Fear that treatment will not work<br>Fear of dying<br>Difficulty with getting back to 'normal life' after treatment<br>Partner or spouse leaving<br>Other issues relating to family and friends<br>Feelings of isolation<br>Feeling unable to talk to others<br>Loss of fertility<br>Regaining sexual intimacy with a partner<br>Coping with the menopause<br>Dealing with stigma because of the cancer diagnosis<br>Other<br>**(IF YOU HAVE SELECTED ANY OF THE ABOVE GO TO Q 39)**<br>None in particular **(GO TO Q41)**
Q39	Which issue have you found most challenging?<br>**SELECT ONLY ONE ANSWER**<br><br>Fear of the cancer returning

	Fear that treatment will not work<br>Fear of dying<br>Difficulty with getting back to 'normal life' after treatment<br>Partner or spouse leaving<br>Other issues relating to family and friends<br>Feelings of isolation<br>Feeling unable to talk to others<br>Loss of fertility<br>Regaining sexual intimacy with a partner<br>Coping with the menopause<br>Dealing with stigma because of the cancer diagnosis<br>Other<br>None in particular
Q40	In connection to your emotional support needs, have you?<br>**TICK ALL THAT APPLY**<br><br>Asked for help from a doctor or nurse<br>Been offered help from a doctor or nurse<br>Been offered psychotherapy from a psychologist<br>Asked for help from family or friends<br>Been offered help from family or friends<br>Asked for help from a charity or non-governmental organisation (NGO)<br>Been offered help from a charity or non-governmental organisation (NGO)<br>Asked for help from a religious organisation/person<br>Been offered help from a religious organisation/person<br>None of the above
Q41	Have you been able to get the emotional support you needed?<br><br>Yes<br>Yes, to some extent<br>Not enough<br>None
Q42	Have you ever met and talked to another woman, or group of women with ovarian cancer since your diagnosis? **TICK ALL THAT APPLY**<br><br>Yes, in person<br>Yes, in person as part of a cancer group<br>Yes online (social media, chat room, forum)<br>Yes, on the telephone<br>No
Q43	Would you like to be able to meet with other women who have ovarian cancer?

	Yes<br>No<br>Not sure

Practical Support needs	
Q44	Which, if any of the following forms of practical support do you feel you need, or have you needed, because of your diagnosis of ovarian cancer?<br>**TICK ALL THAT APPLY**<br><br>Help with daily chores at home (e.g., shopping, cleaning, preparing food, gardening)<br>Help with personal care (e.g., getting dressed, washed, wound care<br>Home adaptations (e.g., using a wheelchair, handrails)<br>Help caring for dependants (parents, siblings, children)<br>Help with transport including travel to and from hospital<br>Financial support<br>Other<br>**(IF YOU HAVE SELECTED ANY OF THE ABOVE GO TO Q45)**<br>I have not needed any practical support **(GO TO Q47)**
Q45 if they had support needs	Which, if any, of the following forms of practical support have you received following your diagnosis and treatment for ovarian cancer. **TICK ALL THAT APPLY**<br><br>Help with daily chores at home (e.g., shopping, cleaning, preparing food, gardening)<br>Help with personal care (e.g., getting dressed, washed, wound care<br>Home adaptations (e.g., using a wheelchair, handrails)<br>Help caring for dependants (parents, siblings, children)<br>Help with transport including travel to and from hospital<br>Financial support<br>Other
Q46	Which group or person has given you the most practical support?<br>**TICK ALL THAT APPLY**<br><br>Family member(s)<br>Friends<br>A charity<br>A non-governmental organisation (NGO)<br>A government agency<br>A religious organisation or person<br>A carer<br>Someone else

	No one in particular
Q47	Has having a diagnosis of ovarian cancer had an impact on your financial situation?<br><br>Yes to a great extent<br>Yes to some extent<br>Not much<br>**(IF YOU SELECTED ANY OF THE ABOVE GO TO Q 48)**<br>Not at all **(GO TO Q49)**<br>I would prefer not to say **(GO TO Q49)**
Q48 if there has been some financial impact	In what way has your financial situation been impacted by your diagnosis? **TICK ALL THAT APPLY**<br><br>I have been unable to work<br>My household income has dropped below what we need to live on<br>I or my family have had to pay for treatment or tests<br>I or my family have had to spend extra money on travelling to or accommodation near the hospital<br>I have had to ask for financial help from family members<br>I have had to ask for financial help from a charity or NGO<br>I now find it hard to find the money for food, rent and bills<br>My partner has been unable to work because they care for me<br>Other

Information needs	
Q49	Since being diagnosed, have you felt in need of information about ovarian cancer? **TICK ALL THAT APPLY**<br><br>Ovarian cancer in general<br>Treatments for ovarian cancer<br>Coping with long term side effects of treatment<br>Clinical trials<br>Genetic testing<br>Living with ovarian cancer<br>Reducing anxiety<br>Symptoms that might indicate a recurrence<br>Survival rates<br>Managing ovarian cancer that can no longer be treated<br>How to talk to family and friends<br>Other<br>**(IF YOU HAVE SELECTED ANY OF THE ABOVE GO TO Q50)**<br>I have not needed any information **(GO TO Q53)**

Q50 (if they needed info)	Have you found the information you needed when you needed it?<br><br>Yes always<br>Yes sometimes<br>Just a little information<br>**(IF YOU HAVE SELECTED ANY OF THE ABOVE GO TO Q51)**<br>Not at all **(GO TO Q52)**
Q51 If they found some of the information	What have been the most important sources of information for you?<br>**SELECT UP TO <u>TWO</u> ANSWERS**<br><br>Your doctor<br>Your nurse<br>Another health professional<br>A charity<br>A non-governmental organisation<br>A Government Agency<br>A website<br>An ovarian cancer support group (online or face to face)<br>A cancer support group (online or face to face)<br>Other women who have had ovarian cancer<br>Other<br>None in particular
Q52	Have you ever searched for information on the internet about your diagnosis?<br>**TICK ALL THAT APPLY**<br><br>Yes, and found good information in my language<br>Yes, but did not find any good information in my language<br>Yes, but did not find useful information<br>Yes, but found information that made me scared<br>Yes, but I could not find information in my language<br>I do not have easy access to the internet<br>Other<br>No, I have not used the internet to search for information
Q53	If this hospital were able to provide women with information about living with ovarian cancer, what do you think it should include?<br>**TICK ALL THAT APPLY**<br><br>Information about treatments and diagnosis<br>Information about living with ovarian cancer and what to expect<br>Information on how to manage physical and mental health<br>Managing ovarian cancer that can no longer be treated<br>Sources of local or national support

	A way to meet other women with ovarian cancer in person or online<br>The hospital already supplies the information I need<br>Other<br>I would not like them to provide information

**Final questions**	
Q54	At this point in time, what factors would mean you have a good quality of life given you are living with ovarian cancer?<br>**TICK ALL THAT APPLY**<br><br>Feeling physically well<br>Feeling mentally well<br>Being able to work<br>Being able to care and support your family<br>Not being a burden on your family<br>Being able to maintain or have a physical relationship with your partner<br>Being able to engage in hobbies and activities<br>Being able to socialise<br>Having a positive self-image<br>Feeling in control of your life<br>Being able to return to 'normal'<br>To be free from the fear of cancer<br>Other
Q55	Do you feel COVID-19 pandemic has had an impact on your treatment and feelings in relation to your cancer? **TICK ALL THAT APPLY**<br><br>I am or have been scared to visit a hospital<br>I worry about catching COVID-19<br>I am worried the pandemic might affect my chance of getting treatment<br>I have worried about whether I should get a vaccine<br>The COVID-19 pandemic has affected my treatment<br>The COVID-19 pandemic contributed to a delay in my diagnosis<br>It has made me feel more isolated<br>Other<br>No, the pandemic has not impacted me in this way
Q56	Would you be willing to consider taking part in a clinical trial, comparing new treatments to the normal standard of care, if they were available at this or another hospital?<br>**TICK ALL THAT APPLY**<br><br>I would not be interested<br>I would like to find out more information first

	I would be interested in taking part in a trial at this hospital<br>I would consider taking part in a clinical trial even if it meant travelling to another hospital
Q57	If money could be invested in improving diagnosis and care of women with ovarian cancer in your country, which areas are most in need of improvement? **SELECT UP TO <u>THREE</u> OPTIONS**<br><br>Development of a screening programme to detect the disease before symptoms develop<br>Ensuring women have free access to diagnostic tests<br>Reducing delays in diagnosis<br>Raising awareness of ovarian cancer and the symptoms<br>Ensuring women have free access to treatments<br>Increasing the number of experienced surgeons<br>Getting access to new drugs that are approved in high income countries<br>Ensuring women at risk of ovarian cancer are identified due to their family history<br>Ensuring women can access clinical trials<br>Funding for research<br>Other
Q58	Do you feel your government could do more to help women with ovarian cancer live a long and good life?<br><br>Definitely<br>Yes to some extent<br>Not really<br>They could not do any more
Q59	Is there something that is particularly important to you about your experience of ovarian cancer that you would like to share with the study team?

Thank you so much for sharing your experiences. If you have any questions or concerns, please speak to the person who invited you to take part in this survey.

16.5. Appendix IV: Every Woman Survey (Kiswahili version)

NAMBARI YA KITAMBULISHO CHA KIPEKEE ……………………………………..

Juu Yako	
Q1	Je unajaza fomu hii kwa uzoefu wako mwenyewe kuhusu saratani ya nyumba ya mayai?<br><br>Ndio – **ENDA KWA Q2**<br>La – **ENDA KWA Q1A**
Q1A (kama jibu ni La kwa swali1)	Kama unajaza fomu hii na mtu ambaye ana saratani ya nyumba ya mayai, ni nini jukumu lako?<br><br>Dakatri/Daktari Mwanafunzi/Muuguzi/Muuguzi Mwanafunzi/Mtafiti/Mfanyikazi wa Kijamii/Nyingine (tafadhali fafanua)<br><br>Maswali yafuatayo ni ya kuuliza mwanamke mwenye saratani ya nyumba ya mayai kuhusu uzoefu wake. Isipokua maelezo machache, tafadhali hakikisha usipendekeze majibu atakayopeana.
Q2	Tafadhali onyesha kabila lako. Kwa ukabila tunamaanisha hisia ya kua kati ya jumuiya ambayo wanaoshiriki ukoo, rangi, lugha, au dini. Waweza chagua chaguo Zaidi ya moja.<br><br>***Each Country Lead Clinician will be asked to supply a standard but useful list from their own country, and include /Mixed/Other/I'd rather not say. Please use the agreed terms here. No need to translate this instruction. Just the question and answers***
Q3	Kwa wastani, inakuchukua muda gani kusafiri kwenda kwa hospitali unayoonekana kuhusiana na saratani ya nyumba ya mayai?<br><br>Chini ya dakika 15<br>Dakika 15-30<br>Dakika 30-60<br>Masaa 1-2<br>Masaa 2-5<br>Zaidi ya masaa 5<br>Zaidi ya masaa 24

Q4	Umesoma shule hadi kiwango gani?<br><br>***(Local teams to select most suitable descriptors – using ISCED levels (International Standard Classification of Education 2011) Please use the agreed levels and get these translated, rather than exact translation of the terms below. If in doubt contact ews@worldovariancancercoalition.org. No need to translate this instruction. Just the question and answers.***<br><br>Hakuna elimu rasmi<br>Shule ya msingi (utotoni, msingi, chekechea, ISCED kiwango cha 1)<br>Shule ya sekondari (shule ya kati, sekondari, ISCED kiwango cha 2,3)<br>Elimu ya juu (chuo cha ufundi, chuo cha jamii, ISCED kiwango cha 4, 5)<br>Chuo kikuu (chuo kikuu au ujuzi sawa wa kitaaluma, ISCED kiwango cha 6)<br>Elimu ya baada ya kuhitimu (Chuo kikuu cha uzamili/Udaktari au sifa za kitaaluma za juu, ISCED kiwango cha 7 or 8)
Q5	Kabla ya kutambulika na saratani ya nyumba ya mayai, ulikua?<br><br>Umeolewa au kuishi na mpenzi<br>Mjane<br>Mpenzi aliyeachwa au aliyetengana<br>Kwenye uhusiano lakini sio kuishi pamoja<br>Pekee yako (hujawai kuolewa)<br>Pendelea kutosema
Q6	Kabla tu ya kutambulika, kwa maoni yako mapato ya mwezi ya famila yako yalikua kiasi gani?<br><br>Chini ya wastani kwa nchi yako<br>Wastani kwa nchi yako<br>Zaidi ya wastani kwa nchi yako<br>Pendelea kutosema
Q7	Karibu na wakati wako wa utambuzi, ulikua na wajibu gani kwa familia yako? **CHAGUA YOTE YANOYAFAA**<br><br>Ndio, mtoto au watoto chini ya umri wa miaka 18<br>Ndio, jamaa wakongwe<br>Ndio, mpenzi mgonjwa<br>Nyingine<br>La, sijakua na wajibu wowote
Q8	Kabla tu utambulike na saratani ya nyumba ya mayai, ulikua?<br><br>Umeajiriwa<br>Umeajiriwa kwa muda

	Umejiajiri<br>Haujaajiriwa<br>Mstaafu<br>Nyingine
Q9	Umewezaje kulipia huduma ya matibabu/utunzaji wa saratani ya nyumba ya mayai hadi wakati huu?<br>**CHAGUA YOTE YANAYOFAA**<br><br>Bima ya matibabu ya kibinafsi<br>Bima ya matibabu ya serikali<br>Nimelipia<br>Wanafamilia wamelipia<br>Nimechangisha pesa ili kufidia gharama kwa njia ya harambee<br>Nimetafuta msaada wa kifedha kutoka shirika lisilo la faida/Shirika lisilo la serikali/Hisani<br>Matibabu ya saratani ya nyumba ya mayai ni bure<br>Vipimo vya uchunguzi vya saratani ya nyumba ya mayai ni bure
Q10	Kuhusiana na historia yako ya uzazi kabla ugunduliwe na saratani ya nyumba ya mayai, **CHAGUA YOTE YANAYOFAA**<br><br>Nilikua nimeingia kwenye ukomo wa hedhi<br>Sijawai kuzaa mtoto<br>Nimejifungua mara moja<br>Nimejifungua mara mbili<br>Nimejifungua mara tatu au zaidi<br>Niliwanyonyesha watoto wangu<br>Nilitumia kidonge cha uzazi wa mpango hadi miaka 5<br>Nilitumia kidonge cha uzazi wa mpango kati ya miaka 5 na 10<br>Nilipata matibabu ya utasa

**Historia ya Familia**	
Q11	Kuna jamaa yeyote kati ya wafuatao (kama, ndugu wa damu kwa upande wa baba ama mama wa familia) wamekua na saratani ya nyumba ya mayai?<br>**CHAGUA YOTE YANAYOFAA**<br><br>Mama<br>Binti<br>Dada<br>Shangazi<br>Binamu<br>Nyanya (pande ya mama)<br>Nyanya (pande ya baba)

	Jamaa wengine wa mbali zaidi (pande ya mama)<br>Jamaa wengine wa mbali zaidi (pande ya baba)<br>La, hakuna hata mmoja wa familia yangu wa karibu ameathiriwa<br>Sijui ama siwezi kumbuka
Q12	Kuna jamaa yeyote kati ya wafuatao (kama, ndugu wa damu kwa upande wa baba ama mama wa familia) wamekua na saratani ya matiti?<br>**CHAGUA YOTE YANAYOFAA**<br>Mama<br>Baba<br>Binti<br>Mwana<br>Dada<br>Ndugu<br>Shangazi (upande wowote)<br>Mjomba (upande wowote)<br>Binamu (upande wowote)<br>Nyanya (upande wowote)<br>Babu (upande wowote)<br>La, hakuna hata mmoja wa familia yangu wa karibu ameathiriwa<br>Sijui ama siwezi kumbuka
Q13	Je unafahamu ya kwamba kua na ndugu wa damu wenye saratani ya nyumba ya mayai, matiti, kongosho, utumbo mpana, nyumba ya kizazi yaweza ongeza hatari ya mwanamke kupata saratani ya nyumba ya mayai?<br><br>Ndio<br>La<br>Sina uhakika

**Kuongoza kufikia utambuzi**	
Q14	Kabla ya kugunduliwa una saratani ya nyumba ya mayai, ulijua nini kuhusu saratani ya nyumba ya mayai?<br><br>Nilikua nimeiskia na nilijua kidogo tu<br>Nilikua nimeskia juu yake lakini sikujua chochote<br>Sikuwai kuwa nimesikia<br>Sijui au siwezi kumbuka
Q15	Kabla ya kugunduliwa na saratani ya nyumba ya mayai kuna dalili zozote zifuatazo ulizopata?<br>**CHAGUA YOTE YANAYOFAA**<br>Maumivu ndani ya tumbo (tumbo)

	Kukojoa mara kwa mara<br>Shida ya kukula/upungufu wa hamu ya kula<br>Mabadiliko kwa kwenda choo (mfano, kuhara au ugumu wa kufanya choo)<br>Uchovu mwingi<br>Kuongezeka kwa ukubwa wa tumbo<br>Kuhisi kushiba kila mara<br>Kupunguza kilo bila sababu<br>Mkojo wa uharaka<br>Maumivu katika nyonga<br>Tumbo kufura mara kwa mara<br>Dalili zingine<br>**(IKIWA UMECHAGUA MOJAWAPO ENDA KWA Q17)**<br>Hakuna kati ya zilizo hapo juu **(ENDA KWA Q16)**
Q16 ikiwa wamejibu 'hakuna kati ya zilizo hapo juu' kwa Q15	Kwasababu hukupata dalili zozote zilizoainishwa katika swali lililotangulia kabla ya utambuzi Q15, ulipata utambuzi wako kivipi?<br>**CHAGUA YOTE YANAYOFAA**<br><br>Uchunguzi wa kawaida<br>Udadisi/uchunguzi wa skan kama kawaida<br>Kipimo cha damu cha kawaida<br>Saratani iligunduliwa wakati nilikua natibiwa kitu kingine<br>Nyingine<br>**ENDA SASA KWA Q27**
Q17 kwa wanawake waliokua na dalili zilizokua zimeorodheshwa katika Q15	Ni wakati gani uligundua dalili zozote zilizochaguliwa hapo juu?<br>Tafadhali andika mwezi na mwaka.<br>Ikiwa hukumbuki, tafadhali wacha tu.<br><br>………………….. mwezi dalili zilianza<br>………………….. mwaka dalili zilianza
Q18	Ulikua na hofu kuhusu dalili ulizokua nazo kabla ya utambuzi?<br><br>Hofu<br>Hofu kiasi<br>Si hofu sana<br>Sikua na hofu<br>Sijui au siwezi kumbuka
Q19	Ulitafuta ushauri kutoka kwa mtu kuhusu dalili zako, kwa mfano daktari, muuguzi, mfamasia au daktari wa kienyeji?<br><br>Ndio **(ENDA KWA Q21)**<br>La **(ENDA KWA Q20)**
Q20<br>Ikiwa watajibu La kwa Q19	Ikiwa hukutafuta ushauri kutoka kwa mtu kuhusu dalili zako, nini kilisababisha utambuzi wako?<br>**CHAGUA YOTE YANAYOFAA**

	Uchunguzi wa kawaida<br>Udadisi/uchunguzi wa skan kwa kawaida<br>Kipimo cha damu cha damu cha kawaida<br>Saratani iligunduliwa wakati nilikua natibiwa kitu kingine<br>Nyingine<br>Other<br>**(ENDA KWA Q26)**
Q21	Ni mtu wa aina gani, kando na mtu wa familia yako, ambaye ulitafuta ushauri kwanza kuhusu dalili zako?<br>**CHAGUA JIBU MOJA TU**<br><br>Daktari wa kienyeji<br>Mtaalamu wa afya mwingine<br>Daktari wa familia<br>Daktari wa magonjwa ya wanawake<br>Daktari wa magonjwa ya uzazi (daktari mtaalamu anayehusika na matibabu ya saratani ya nyumba ya mayai)<br>Daktari wa gastroenterolojia<br>Chumba cha dharura au ajali na daktari wa dharura<br>Muuguzi<br>Mfamasia<br>Mtu mwingine
Q22	Mbali na mtu wa kwanza, ambaye ulitafuta mawaidha kutoka kuhusu dalili, nani mwingine ulitembelea ama kuongea naye kuhusu dalili kabla ya wewe kutambulika?<br>**CHAGUA YOTE YANAYOFAA**<br><br>Daktari wa kienyeji<br>Mtaalamu wa afya mwingine<br>Daktari wa familia<br>Daktari wa magonjwa ya wanawake<br>Daktari wa magonjwa ya uzazi (daktari mtaalamu anayehusika na matibabu ya saratani ya nyumba ya mayao)<br>Daktari wa gastroenterolojia<br>Chumba cha dharura au ajali na daktari wa dharura<br>Muuguzi<br>Mfamasia<br>Mtu wa familia yako<br>Mtu mwingine yeyote<br>Hakuna mtu mwingine

Q23	Ni lini mara ya kwanza ulipomuona **daktari wa matibabu** kuhusu dalili zako?<br><br>Tafadhali weka mwezi, na mwaka.<br>Kama hukumbuki, tafadhali wacha tu.<br><br>………………….. Mwezi uliomtembelea daktari wa matibabu kuhusu dalili<br>………………….. Mwaka uliomtembelea daktari wa matibabu kuhusu dalili
Q24	Kwa maoni yako, ni kwa umakini gani daktari wa matibabu uliyemuona wa kwanza kuhusu hofu za dalili zako?<br><br>Kwa umakini sana<br>Makini kidogo<br>Sio kwa umakini sana<br>Sio kwa umakini<br>Sijui ama siwezi kumbuka
Q25	Takriban mara ngapi uliongea au kuwatembelea dakatari wa matibabu (daktari wa aina yoyote, sio yule tu uliyemuona wa kwanza) kabla uambiwe una saratani ya nyumba ya mayai?<br><br>Ikiwa huwezi kumbuka, tafadhali wacha tu.<br><br>___ (Eka nambari)
Q26	Unahisi kana kwamba muda ulipoanza kuskia dalili hadi kutambulika na saratani ya nyumba ya mayai ungefupishwa?<br><br>Ndio<br>La<br>Sina uhakika<br><br>Tafadhali tumia sanduku la MAONI kutujulisha ni vipi wakati ungefupishwa (kwa mfano kuchelewa kwa daktari, ucheleweshaji wa mfumo wa afya kama vile kungoja vipimo au ziara za hospitali) au mambo ambayo huenda yakakucheleweshsa kutafuta msaada.

	MAONI
Q27	Ni lini uliambiwa na daktari wa matibabu una saratani ya nyumba ya mayai?<br><br>Tafadhali weka mwezi, na mwaka.<br>Kama hukumbuki, tafadhali wacha tu.<br><br>………………….. Mwezi ulioambiwa na daktari wa matibabu unayo saratani ya ovari<br>………………….. Mwaka ulioambiwa na daktari wa matibabu unayo saratani ya ovari

Matibabu ya saratani ya nyumba ya mayai	
Q28	Ni lini kwanza ulipoanza matibabu yoyote ya saratani ya nyumba ya mayai, kama vile upasuaji au kidini?<br><br>Ikiwa huwezi kumbuka, au hujapata matibabu yoyote tafadhali wacha tu<br><br>………………….Mwezi matibabu yalianza<br>………………….Mwaka matibabu yalianza
Q29	Kwa uamuzi wako ni matibabu gani utatumia kudhibiti saratani yako ya nyumba ya mayai au jinsi ya kukabiliana na madhara kutoka kwa matibabu, gani zifuatazo zitaathiri uamuzi huo?<br>**CHAGUA YOTE YANAYOFAA**<br><br>Maoni ya daktari<br>Maoni ya familia yangu<br>Nitaamua mwenyewe

	Gharama ya dawa za matibabu<br>Gharama zingine zinazohusiana na matibabu kama vile usafiri au chumba cha malazi<br>Uwezekano wa kupona au kuongeza miaka ya maisha yangu<br>Madhara ya matibabu<br>**(IKIWA UMECHAGUA MOJAWAPO ENDA KWA Q30)**<br>Hakuna kati ya zilizo hapa juu **(ENDA KWA Q31)**
Q30	Sentensi gani ulizozichagua katika swali lililopita Q29 ndizo za maana sana katika uamuzi wako?<br>**CHAGUA JIBU <u>MOJA</u> TU**<br><br>Maoni ya daktari<br>Maoni ya familia yangu<br>Nitaamua mwenyewe<br>Gharama ya dawa za matibabu<br>Gharama zingine zinazohusiana na matibabu kama vile usafiri au chumba cha kulala<br>Uwezekno wa kupona au kuongeza miaka ya maisha yangu<br>Madhara ya matibabu
Q31	Kwa ujumla, je unajiskia umejishirikisha kwa kiwango ungetaka, kuhusu maamuzi ya matibabu yako katika utunzaji wa saratani, kama vile kuamua matibabu gani au ikiwa utapata matibabu yoyote?<br><br>Ndio kila mara<br>Ndio mara zingine<br>Wakati mwingine tu<br>La<br>Sijui au siwezi kumbuka
Q32	Kati ya madhara yafuatayo ya matibabu ya saratani ya nyumba ya mayai, ni zipi ambazo zimekua ngumu zaidi kukabiliana nazo tangu uanze matibabu?<br>**CHAGUA MAJIBU <u>MAWILI</u>**<br><br>Sijakua na matibabu yoyote (ENDA KWA Q35)<br>Kuchoka au uchovu<br>Wasiwasi<br>Kuhisi/kujiskia kuwa mgonjwa (kichefuchefu au kutapika)<br>Kuhara/kuendesha<br>Kupoteza hamu ya kula<br>Kutokuwa na uwezo wa kupambana na maambukizi<br>Upungufu wa nywele au kupoteza nywele<br>Ngozi kukauka<br>Uchungu kwa kinywa

	Kuwashwa au kufa ganzi kwa mikono au miguu<br>Kupoteza usingizi<br>Ladha ya chuma kinywani<br>Ugumu wa kufanya choo<br>Mtutumko wa mzio<br>Maumivu ya viungo<br>Uvimbe<br>Maumivu ya misuli<br>Dalili ya kukoma hedhi<br>Matatizo baada ya upasuaji<br>Athari nyingine<br>**(IKIWA UMECHAGUA MOJAWAPO ENDA KWA Q33)**<br>Hakuna madhara haswa **(ENDA KWA Q35)**
Q33 ikiwa walikua na madhara	Je waweza eleza madhara haya kuwa 'muda mrefu' au yamekua yakiendelea kwa wiki kadhaa au miezi baada ya matibabu kukamilika?<br><br>Ndio<br>La<br>Bado naendelea na matibabu kwahivyo siwezi sema kanakwamba yatakua ya muda mrefu<br>Sina uhakika
Q34	Je wataalamu wa afya wameweza kusaidia kupunguza athari za madhara kwa kutumia madawa au ushauri na maelezo?<br><br>Ndio<br>Ndio kwa kiasi fulani<br>La<br>Sikutafuta/sikuhitaji kutafuta msaada kuhusu madhara<br>Sijui au siwezi kumbuka
Q35	Je kuna wakati wowote toka utambuzi wako, umetumia dawa za mitishamba, dawa sawa na au matibabu mbadala ambayo hukuandikiwa na daktari hospitalini, kujaribu kudhibiti saratani ya nyumba ya mayai, au uchungu ambao umeletwa na saratani hio?<br><br>Ndio wakati wote<br>Ndio wakati fulani<br>La, lakini sijafikiria bado<br>La<br>Sijui
Q36	Je umewai taka kupata maoni mengine kutoka daktari mwingine kuhusu saratani yako ya nyumba ya mayai na matibabu?<br><br>La

	Ndio na nimeweza kupata<br>Ndio lakini sijui nimuulize nani<br>Sijafikiria bado

Mahitaji ya msaada wa kihisia	
Q37	Je, kuna wakati wowote **umejiskia** iwapo unahaja ya msaada wa kihisia?<br>**CHAGUA YOTE YANAYOFAA**<br><br>Wakati wa utambuzi<br>Wakati wa matibabu<br>Baada ya matibabu kukamilika<br>Wakati saratani ilirejea<br>Nilipoambiwa saratani haina tiba<br>Nyingine<br>**(IKIWA UMECHAGUA MOJAWAPO ENDA KWA Q38)**<br>Sijahitaji msaada wa kihisia **(ENDA KWA Q42)**
Q38<br>Ikiwa wamehitaji msaada wa kihisia	Je, kuna mambo haswa umekabiliana nayo?<br>**CHAGUA YOTE YANAYOFAA**<br><br>Hofu ya saratani kurejea<br>Hofu kuwa matibabu hayatafanya kazi<br>Hofu ya kufa<br>Ugumu wa kurudia 'maisha ya kawaida' baada ya matibabu<br>Mpenzi kukuacha<br>Masuala mengine yanayohusiana na familia na marafiki<br>Hisia za kutengwa<br>Kushindwa kuzungumza na wengine<br>Kupoteza ugumba<br>Kurudisha hamu ya kujamiiana na mumeo<br>Kukabiliana na kukoma kwa hedhi<br>Kukabiliana na unyanyapaa kwa ajili ya utambuzi wa saratani<br>Nyingine<br>**(IKIWA UMECHAGUA MOJAWAPO ENDA KWA Q39)**<br>Hakuna haswa **(ENDA KWA Q41)**
Q39	Ni masuala gani umepata kuwa changamoto zaidi?<br>**CHAGUA JIBU <u>MOJA</u> TU**<br><br>Hofu ya saratani kurejea<br>Hofu kuwa matibabu hayatafanya kazi<br>Hofu ya kufa<br>Ugumu wa kurudia 'maisha ya kawaida' baada ya matibabu<br>Mpenzi kukuacha<br>Masuala mengine yanayohusiana na familia na marafiki

	Hisia za kutengwa<br>Kushindwa kuzungumza na wengine<br>Kupoteza ugumba<br>Kurudisha hamu ya kujamiiana na mumeo<br>Kukabiliana na kukoma kwa hedhi<br>Kukabiliana na unyanyapaa kwa ajali ya utambuzi wa saratani<br>Nyingine<br>Hakuna haswa
Q40	Pamoja na mahitaji yako ya kihisia, umewai?<br>**CHAGUA YOTE YANAYOFAA**<br><br>Omba msaada kutoka kwa daktari au muuguzi<br>Pewa msaada kutoka kwa daktari au muuguzi<br>Pewa matibabu ya kisaikolojia kutoka kwa mwanasaikolojia<br>Omba msaada kutoka familia au marafiki<br>Pewa msaada kutoka familia na marafiki<br>Omba msaada kutoka kwa hisani au shirika lisilo la kiserkali (NGO)<br>Pewa msaada kutoka kwa hisani au shirika lisilo la kiserikali (NGO)<br>Omba msaada kutoka kwa shirika la dini/mtu<br>Pewa msaada kutoka kwa shirika la dini/mtu<br>Hakuna yoyote
Q41	Umeweza kupata msaada wa kihisia ambao ulihitaji?<br><br>Ndio<br>Ndio kwa kiasi fulani<br>Haitoshi<br>Sijapata
Q42	Je, umewahi kutana na kuongea na mwanamke mwingine, au kundi la wanawake wenye saratani ya nyumba ya mayai kutoka utambuzi wako?<br>**CHAGUA YOTE YANAYOFAA**<br><br>Ndio, kwa kibinafsi<br>Ndio, kama mwanachama wa kikundi cha saratani<br>Ndio mtandaoni (mtandao wa kijamii, chumba cha mazungumzo, jukwaa)<br>Ndio, kwa simu<br>La
Q43	Na ungependa kukutana na wanawake wengine wenye saratani ya nyumba ya mayai?<br><br>Ndio<br>La<br>Sina uhakika

Mahitaji ya msaada wa vitendo	
Q44	Je, kama ziwepo ni aina za msaada za vitendo unaona unahitaji, au umewahi hitaji, kwa ajili ya utambuzi wako wa saratani ya nyumba ya mayai?<br>**CHAGUA YOTE YANAYOFAA**<br><br>Msaada Kwa kazi za kila siku za nyumbani (kwa mfano, ununuzi wa vitu, kusafisha, kuandaa chakula, kutunza bustani)<br>Msaada wa utunzaji wa kibinafsi (kwa mfano, kuvaa, kuoshwa, utunzaji wa vidonda)<br>Marekebisho ya nyumbani (kwa mfano, kwa kutumia kiti cha magurudumu, reli za mikono)<br>Msaada wa kutunza wanaokutegemea (wazazi, ndungu, watoto)<br>Msaada wa usafiri kuenda na kurudi hospitalini<br>Msaada wa kifedha<br>Nyingine<br>**(IKIWA UMECHAGUA MOJAWAPO ENDA KWA Q45)**<br>Sijahitaji msaada wowote wa vitendo **(ENDA KWA Q47)**
Q45 ikiwa walikua na msaasa wa mahitaji	Je, kama ziwepo ni aina gani za msaada wa vitendo umewai pata kufuatia utambuzi wako na matibabu ya saratani ya nyumba ya mayai?<br>**CHAGUA YOTE YANAYOFAA**<br><br>Msaada wa kazi za kila siku za nyumbani (kwa mfano, ununuzi wa vitu, kusafisha, kuandaa chakula, kutunza bustani)<br>Msaada wa utunzaji wa kibinafsi (kwa mfano, kuvaa, kuoshwa, utunzaji wa vidonda)<br>Marekebisho ya nyumbani (kwa mfano, kwa kutumia kiti cha magurudumu, reli za mikono)<br>Msaada wa kutunza wanaokutegemea (wazazi, ndungu, watoto)<br>Msaada wa usafiri kuenda na kurudi hospitalini<br>Msaada wa kifedha<br>Nyingine
Q46	Ni kikundi kipi au mtu yupi ambaye amekupa msaada wa vitendo zaidi?<br>**CHAGUA YOTE YANAYOFAA**<br><br>Mtu wa familia<br>Marafiki<br>Hisani<br>Shirika lisilo la serikali (NGO)<br>Wakala wa serikali<br>Shirika la dini/mtu<br>Mlezi

	Mtu mwingine<br>Hakuna mtu
Q47	Je, utambuzi wa saratani ya nyumba ya mayai umekua na athari kuhusu hali yako ya kifedha?<br><br>Ndio sana<br>Ndio kwa kiasi fulani<br>Sio sana<br>**(IKIWA UMECHAGUA MOJAWAPO ENDA KWA Q48)**<br>Hukuna kabisa **(ENDA KWA Q49)**<br>Ningependelea kutosema **(ENDA KWA Q49)**
Q48 iwapo kumekua na athari fulani za kifedha	Je, ni kwa njia ipi hali yako ya kifedha imeathiriwa na utambuzi wako? **CHAGUA YOTE YANAYOFAA**<br><br>Nimeshindwa kufanya kazi<br>Mapato yangu ya familia yamekua ya chini kushinda vile tunavyoishi<br>Mimi au familia yangu imetubidi kulipia matibabu au vipimo<br>Mimi au familia yangu imetubidi tutumie pesa za ziada kusafiri kuenda au kupata chumba cha malazi karibu na hospitali<br>Nimelazimika kuomba msaada wa kifedha kutoka kwa watu wa familia<br>Nimelazimika kuomba msaada wa kifedha kutoka kwa shirika la hisani au lisilo la serikali<br>Kwa sasa ninapata shida kutafuta pesa za chakula, kukodisha nyumba na majukumu ya kifedha<br>Mpenzi wangu ameshindwa kufanya kazi kwasababu ya kunitunza<br>Nyingine

Mahitaji ya maelezo	
Q49	Kutoka utambuzi, umekua na haja ya kupata maelezo kuhusu saratani ya nyumba ya mayai? **CHAGUA YOTE YANAYOFAA**<br><br>Saratani ya nyumba ya mayai kwa ujumla<br>Matibabu ya saratani ya nyumba ya mayai<br>Kukabiliana na athari za muda mrefu za matibabu<br>Majaribio ya kliniki<br>Uchunguzi wa maumbile<br>Kuishi na saratani ya nyumba ya mayai<br>Kupunguza wasiwasi<br>Dalili ambazo zinaweza kuonyesha kurejea kwa saratani<br>Viwango vya kuishi<br>Kudhibiti Saratani ya nyumba ya mayai isiyotibika<br>Jinsi ya kuongea na familia na marafiki

	Nyingine<br>**(IKIWA UMECHAGUA MOJAWAPO ENDA KWA Q50)**<br>Sijahitaji kupata maelezo yoyote **(ENDA KWA Q53)**
Q50 (ikiwa walihitaji maelezo)	Je, Uliweza kupata ujumbe uliohitaji?<br><br>Ndio<br>Ndio wakati mwingine<br>Maelezo kidogo tu<br>**(IKIWA UMECHAGUA MOJAWAPO ENDA KWA Q51)**<br>La **(ENDA KWA Q52)**
Q51 ikiwa walipata baadhi ya maelezo	Je, ni njia zipi zimekua muhimu kwako zaidi kupata maelezo?<br>**CHAGUA MAJIBU <u>MAWILI</u>**<br><br>Daktari wako<br>Muuguzi wako<br>Mtaalamu mwingine wa afya<br>Hisani<br>Shirika lisilokuwa la serikali<br>Wakala wa serikali<br>Tovuti<br>Kikundi cha usaidizi wa saratani ya nyumba ya mayai (mtandaoni au ana kwa ana)<br>Kikundi cha msaada cha saratani (mtandaoni au ana kwa ana)<br>Wanawake wengine ambao wamekua na saratani ya nyumba ya mayai<br>Nyingine<br>Hakuna haswa
Q52	Je, umewai tafuta maelezo mtandaoni kuhusu utambuzi huu?<br>**CHAGUA YOTE YANAYOFAA**<br><br>Ndio, na nikapata maelezo mazuri kwa kilugha<br>Ndio, lakini sikupata maelezo mazuri kwa kilugha<br>Ndio, lakini sikupata maelezo ya maana<br>Ndio, lakini nilipata maelezo yaliyonitia hofu<br>Ndio, lakini sikupata maelezo kwa kilugha<br>Sio rahisi kuingia kwenye mtandao<br>Nyingine<br>La, sijatumia mtandao kutafuta maelezo
Q53	Ikiwa hosptali hii ingeweza kupea wanawake maelezo kuhusu kuishi na saratani ya nyumba ya mayai, wafikiri yapaswa kujumuisha nini?<br>**CHAGUA YOTE YANAYOFAA**<br><br>Maelezo kuhusu matibabu na utambuzi

	Maelezo kuhusu kuishi na saratani ya nyumba ya mayai na nini cha kutarajia<br>Maelezo kuhusu jinsi ya kudhibiti afya ya kimwil na kiakili<br>Kudhibiti saratani ya nyumba ya mayai ambayo isiyotibika<br>Usaidizi nchini au wa kimataifa<br>Njia za kukutana na wanawake wengine wenye saratani ya nyumba ya mayai ana kwa ana au mtandaoni<br>Hospitali tayari hupeana maelezo ninayohitaji<br>Nyingine<br>Singependa watoe maelezo

Maswali ya mwisho	
Q54	Je, ni mambo gani kwa wakati huu yanaweza maanisha kuweza kua na maisha bora ikiwa unaishi na saratani ya nyumba ya mayai?<br>**CHAGUA YOTE YANAYOFAA**<br><br>Kujihisi kamili mwilini<br>Kujihisi kamili kiakili<br>Kuwa na uwezo wa kufanya kazi<br>Kuwa na uwezo wa kutunza na kusaidia familia<br>Kutokua mzigo mzito kwa familia yako<br>Kuwa na uwezo wa kudumisha au kuwa na uhusiano wa kimwili na mumeo<br>Kuwa na uwezo wa kufanya mambo upendayo na mambo mengine<br>Kuwa na uwezo wa kushirikiana na watu wengine<br>Kujiona sawa kimaisha<br>Kuwa na udhibiti maishani mwako<br>Kuweza kurudi kuishi maisha ya ‘kawaida’<br>Kuwa huru kutokana na hofu ya saratani<br>Nyingine
Q55	Je, unafikiri kuwa janga kubwa la COVID-19 limekua na athari kwa matibabau na hisia zinazohusiana na saratani yako?<br>**CHAGUA YOTE YANAYOFAA**<br><br>Ninaogopa au nimeogopa kutembelea hospitali<br>Nina wasiwasi kuambukizwa COVID-19<br>Nina wasiwasi janga hili laweza athiri nafasi yangu ya kupata matibabu<br>Nimekua nashangaa ikiwa nipate chanjo<br>Janga la COVID-19 limeathiri matibabu yangu<br>Janga la COVID-19 lilichangia kwa kuchelewa kwa utambuzi wangu<br>Limefanya nihisi kutengwa zaidi<br>Nyingine<br>La, janga halijaniathiri kwa njia yoyote

Q56	Je, ungependa kushiriki katika jaribio la kimatibabu, ya kulinganisha matibabu mapya na yale ya kawaida kwa hospitali hii au nyingine?<br>**CHAGUA YOTE YANAYOFAA**<br><br>La singependa<br>Kwanza ningependa kupata maelezo zaidi<br>Ningependa kushiriki katika jaribio kwa hospitali hii<br>Ningependa kushiriki katika jaribio la kimatibabu hata kama ni kusafiri kwenda hospitali nyingine
Q57	Iwapo fedha zingewekezwa katika kuboresha utambuzi na utunzaji wa wanawake walio na saratani ya nyumba ya mayai katika nchi yako, ni maeneo yapi yanahitaji kuboreshwa zaidi?<br>**CHAGUA CHAGUO <u>TATU</u>**<br><br>Uundaji wa uchunguzi upya wa kutambua saratani kabla dalili kuanza<br>Uhakikishaji kuwa wanawake wanapata vipimo vya bure vya uchunguzi<br>Kupunguza ucheleweshaji wa utambuzi<br>Kuongeza ufahamu wa saratani ya nyumba ya mayai na dalili zake<br>Uhakikishaji kuwa wanawake wanapata matibabu ya bure<br>Kuongeza idadi ya madaktari bingwa wa upasuaji<br>Kuweza kupata dawa mpya zenye kibali zilizokubalika kutoka nchi zenya mapato ya juu<br>Kuhakikisha wanawake walio hatarini ya kupata saratani ya nyumba ya mayai wanatambuliwa kutokana na historia ya familia zao<br>Kuhakikisha wanawake wanaweza pata majaribio ya kimatibabu<br>Ufadhili wa utafiti<br>Nyingine
Q58	Je, wafikiri serikali yako yaweza fanya zaidi kusaidia wanawake wenye saratani ya nyumba ya mayai kuishi maisha marefu na mazuri?<br><br>Ndio, kabisa<br>Ndio kwa kiasi fulani<br>La<br>Hawawezi fanya zaidi
Q59	Kuna kitu ambacho ni muhimu kwako kuhusu uzoefu wako wa saratani ya nyumba ya mayai ungependa kuelezea timu ya utafiti?

Asanti sana kwa kushiriki kutueleza uzoefu wako. Ikiwa una maswali au hofu, tafadhali ongea na mtu ambaye alikualika kushiriki katika utafiti huu.
