# Supplementary material for: The every woman study™ low- and middle-income countries edition protocol: A multi-country observational study to assess opportunities and challenges to improving survival and quality of life for women with ovarian cancer
Source: PLoS One. 2024 May 29;19(5):e0298154. doi: 10.1371/journal.pone.0298154 (PMC11135759; doi:10.1371/journal.pone.0298154)
Supplement: S3 File — (PDF) [file pone.0298154.s004.pdf]

EVERY WOMAN STUDY™ – LOW- AND MIDDLE-INCOME COUNTRY EDITION
COUNTRY LEAD CLINICIAN INTERVIEWS – PRO FORMA

Date of interview:

**Confirm**	Country of residence<br><br>Name of clinician<br><br>Job title
**Consent**	Have you had a chance to read the information about the study, what we will be asking, and how the information will be used?<br><br>Do you have any questions before we begin?<br><br>Do you give your consent to taking part in this interview, in the way we have outlined?
**Overview of ovarian cancer care in their country**	In your opinion, what are some key issues and/or challenges in ovarian cancer care in your country?<br><br>Prompt if needed: Factors you could consider include the availability/quality of ovarian cancer data, national cancer strategy, and/or other resource availability (e.g., diagnostics and treatments).
**Cultural context overview and opportunities to improve health outcomes**	Are there cultural beliefs and practices in your community that affect women with ovarian cancer and the care they receive?<br><br>Prompt if needed: Please consider all aspects from the role of women, routes to diagnosis, to treatments, to expectations regarding health for women.

	Are there any specific examples that might come to mind that might help understand the cultural contexts affecting ovarian cancer care more effectively?<br><br>Are there any particular issues that you feel could and should be addressed? Please indicate whether this relates to ovarian cancer specifically or would relate to other disease areas as well.
**Impact of COVID-19**	What has the impact of the COVID-19 pandemic been on the diagnosis, and treatment for women with ovarian cancer in your country. Please describe how that has changed (if it has) during the course of the pandemic.<br><br>What do you regard as the biggest impact in terms of your ability to care for women with ovarian cancer?<br><br>What are the biggest challenges that currently remain in relation to COVID-19?<br><br>How would you describe the situation now, at this point in time, in terms of cancer care being back to "normal"?<br><br>What do you think are some opportunities, if any, provided by the pandemic to improve cancer care?
**Memorable cases**	Have there been women with ovarian cancer whose cases made a significant impact on you, and the way you think about and care for women with the disease? If you are comfortable sharing, please share why they were so memorable, but without giving names/locations.

**Opportunities for improvement**	What do you think are some of the opportunities and priorities for improvement and change in ovarian cancer care in your country? Any thoughts on what approaches could be taken to address such opportunities in your country?
**Final thoughts**	Many thanks for taking part in this interview and providing your valuable insight.<br><br>Is there anything else that you wish had been discussed/highlighted about ovarian cancer in your country?<br><br>Any other final reflections?
**Consent**	Can I now check with you, that if needed, the World Ovarian Cancer Coalition may use short verbatim extracts (either written or spoken) in the final report? If so, are you happy for your full name to be used? (If not, then first name, or alias). And would you require sign-off?
