# Supplementary material for: The every woman study™ low- and middle-income countries edition protocol: A multi-country observational study to assess opportunities and challenges to improving survival and quality of life for women with ovarian cancer
Source: PLoS One. 2024 May 29;19(5):e0298154. doi: 10.1371/journal.pone.0298154 (PMC11135759; doi:10.1371/journal.pone.0298154)
Supplement: S4 File — (PDF) [file pone.0298154.s005.pdf]

THE EVERY WOMAN STUDY™ LOW- and MIDDLE-INCOME EDITION

Clinician Pro-Forma

This Pro-Forma is to be completed by the Lead in each of the countries taking part in the Every Woman Study. The answers will form the basis of a report that can be used in conjunction with the findings from the country, and to provide context in relation to any of the reports produced. As such it will form an integral part of the Study's results.

You will be very unlikely to complete it in one go. You may need to research some of your answers. Please answer questions to the best of your ability.

THE EVERY WOMAN STUDY™ LOW- and MIDDLE-INCOME EDITION

Clinician Pro-Forma

Information about the Lead Clinician

1. Which country does this information refer to?

2. What is your name?

3. How do you identify?

- ○ Male
- ○ Female
- ○ Other
- ○ Prefer not to say

4. In which year did you complete your general medical training? e.g. 2004

5. Where did you complete your general medical training?

City

Country

6. Have you received any training regarding caring for women with ovarian cancer? Please supply details of type and level, e.g. sub-speciality certified, mentorship, fellowship or informal.

7. How long have you been treating women with ovarian cancer? Please enter the number of years.

8. What is the name and location of the hospital in which you are currently working (and contributing to the study from)?

Hospital name

Hospital location

9. What kind of hospital is the centre you work in?

- A state or public hospital
- A hospital that sees both private and public/state funded patients
- A private hospital
- Other (please specify)

10. What proportion of the women with ovarian cancer that you are involved in diagnosing and treating are private patients (self-paying, or paid for by insurance)?

Enter the % (e.g. 30%). If you do not see any self-paying or patients covered by insurance, please enter 0.

11. What is your job title? Tick the answer that best applies.

- Gynecologist
- Gynecologic Oncologist
- Medical or Clinical Oncologist specialising in gynecological cancers
- Medical or Clinical Oncologist (not specialising in ovarian cancer)
- Radiation Oncologist
- Other

Clinician Pro-Forma

Information about the Lead Clinician (Continued)

12. If you answered other, what is your main job?

- ◯ Emergency doctor
- ◯ GI Internist (Gastroenterologist)
- ◯ General Surgeon
- ◯ Hepatologist
- ◯ Internal Medicine Doctor
- ◯ Urologist
- ◯ Other (please specify)

13. What role do you perform in relation to women with ovarian cancer? Tick all that apply.

- ☐ Lead clinician in a department looking after women with ovarian cancer
- ☐ Assessing women who may have ovarian cancer
- ☐ Diagnosing women
- ☐ Surgery
- ☐ Systemic Therapies
- ☐ Follow up
- ☐ Pain and symptom management
- ☐ Palliative Care
- ☐ Other (please specify)

14. Have you had, or do you have contacts with national or regional health policy makers and government health officials in connection with ovarian cancer? If yes, please provide an outline in the Comments box.

- ◯ No
- ◯ Yes

If yes, please specify:

15. Might you be willing to participate in national/international media coverage relating to the Every Woman Study, if the chance arose? Support could be provided by the Coalition.

- ○ No
- ○ Yes
- ○ Not sure

16. Do you believe that the Every Woman Study will provide opportunities to improve care or quality of life for women with ovarian cancer in your country? If so, please outline your thoughts.

[ ]

17. How would you describe this hospital?

- ☐ Cancer centre
- ☐ University Teaching Hospital
- ☐ General hospital
- ☐ Community hospital
- ☐ Private clinic
- ☐ Other (please specify)

[ ]

THE EVERY WOMAN STUDY™
LOW- and MIDDLE-INCOME EDITION

Clinician Pro-Forma

About the Hospitals and Clinics in the Study

Please complete the following questions for your centre. For assessment of resource level, please use the definition below, used in the ASCO Stratified Guidelines (Vanderpuye et al). If you do not know the answers, please consult with your fellow investigators.

Basic: Core resources or fundamental services that are absolutely necessary for any public health/primary health care system to function; basic-level services typically are applied in a single clinical interaction. Vaccination is feasible for highest need populations.

Limited: Second-tier resources or services that are intended to produce major improvements in outcome such as incidence and cost-effectiveness and are attainable with limited financial means and modest infrastructure; limited-level services may involve single or multiple interactions. Universal public health interventions feasible for greater percentage of population than primary target group.

Enhanced: Third-tier resources or services that are optional but important; enhanced-level resources should produce further improvements in outcome and increase the number and quality of options and individual choice. (Perhaps ability to track patients and links to registries).

Maximal: May use high-resource settings' guidelines. High-level/state-of-the art resources or services that may be used/available in some high-resource countries and/or may be recommended by high-resource setting guidelines that do not adapt to resource constraints but that nonetheless should be considered a lower priority than those resources or services listed in the other categories on the basis of extreme cost and/or impracticality for broad use in a resource-limited environment.

18. **Lead Centre:**

Number of women with ovarian cancer seen each year (newly diagnosed, attending for treatment or follow up)?

[ ]

19. Is this number?

- ◯ Based on local data
- ◯ An estimate
- ◯ I do not know
- ◯ Other (please specify)

[ ]

20. How would you describe the resource level of this hospital according to the definition above?

- ◯ Basic
- ◯ Limited
- ◯ Enhanced
- ◯ Maximal
- ◯ Other (please specify)

[ ]

21. Please provide some commentary on the mix of hospitals taking part in this Study in your country. Please reflect on size, location, diversity, socio-economic groupings, resource stratification, overall function (specialist or not) and to what extent these factors may be considered representative of care of women with ovarian cancer in your country.

THE EVERY WOMAN STUDY™
LOW- and MIDDLE-INCOME EDITION

Clinician Pro-Forma

Data Collection

22. What data is routinely collected in relation to women with ovarian cancer at the main hospital you work in?

- ☐ None
- ☐ Age on diagnosis
- ☐ Type of ovarian cancer
- ☐ Stage of ovarian cancer
- ☐ Family history
- ☐ Other (please specify)

23. Is this information routinely collated (i.e. collected for analysis in one central record)?

- ☐ No
- ☐ Yes, on paper
- ☐ Yes, electronically
- ☐ Other (please specify)

24. Do you share your data with other centres in your country that treat women with ovarian cancer?

- ○ Not routinely
- ○ Yes, at a regional level
- ○ Yes, at a country level
- ○ Other (please specify)

Clinician Pro-Forma

Data Collection (Continued)

25. Will participating in the Every Woman Study mean an increase in the quantity and type of data collected on women with ovarian cancer in your country?

- ◯ Yes
- ◯ No
- ◯ Not sure
- ◯ Other (please specify)

26. Will participating in the Every Woman Study mean an increase in data sharing within your country in relation to women with ovarian cancer?

- ◯ Yes
- ◯ No
- ◯ Not sure
- ◯ Other (please specify)

THE EVERY WOMAN STUDY™
LOW- and MIDDLE-INCOME EDITION

Clinician Pro-Forma

National Cancer Control Plan

27. According to our research, Argentina, Bangladesh, Brazil, Egypt, Ghana, Guatemala, India, Jamaica, Kazakhstan, Kenya, Malawi, Malaysia, Morocco, Mozambique, Nigeria, Peru, South Africa, and Zambia have national cancer control plans. Colombia, Nepal, Uganda, Uzbekistan, and Vietnam do not have national cancer control plans. Is this information correct? Please let us know whether your country has a national cancer control plan.

28. Does your country's national cancer control plan mention ovarian cancer?

- ◯ Yes
- ◯ No

Any comments?

THE EVERY WOMAN STUDY™
LOW- and MIDDLE-INCOME EDITION

Clinician Pro-Forma

Time to Diagnosis

29. Do any of the following statements reflect key issues relating to time to diagnosis in your country? Tick all that apply.

- ☐ Women do not realise their symptoms could be serious
- ☐ It is not easy for women to seek help from a health professional
- ☐ Cultural norms mean that women’s health is not a priority
- ☐ People with cancer face significant stigma
- ☐ The cost of diagnostic investigations means women do not pursue help
- ☐ Doctors women consult do not realise that ovarian cancer may be the cause of a woman’s symptoms
- ☐ Doctors do not have access to CA125 or transvaginal ultrasound for initial investigations
- ☐ The distance women may have to travel for investigations, and associated costs may be too high
- ☐ Other - please add any further thoughts below:

30. In terms of diagnostic testing in your country, what works well, and what needs to improve? Please consider clinical examination, CA125, Abdominal ultrasound, Transvaginal Ultrasound, X-Ray, MRI, PET Scan, CT Scan.

Works well:

Needs improvement:

31. Are there any local, national, or international guidelines that are used in your country relating to the assessment and diagnosis of women who may have ovarian cancer? Please provide the link for the guidelines or the name of the guidelines.

- ◯ No
- ◯ Not sure
- ◯ Yes (please provide a link below)

Clinician Pro-Forma

Surgery for Ovarian Cancer

32. In your view, do the women who receive treatment and care for ovarian cancer at your centre get the best possible surgery they could? Please consider the number of women undergoing surgery, training of surgeons, access to theatre space, duration of surgery, access to pathology, anaesthesiology, intensive care, multidisciplinary approach, waiting time for surgery, workforce availability, and any other issues you think are important.

33. Are there any examples of good practice you'd like to share?

34. What are the main opportunities to improve the number of women who can undergo and gain benefit from the surgery they receive?

35. What are the key barriers to improving surgery that need to be overcome (in order of importance)?

Most important

Next most important

Third most important

Fourth most important

Others

36. In your centre, what is the average duration of surgery for women with ovarian cancer?

37. How would this compare to other participating centres or hospitals in your country?

38. Are there any other aspects you would like us to note in relation to surgery?

THE EVERY WOMAN STUDY™
LOW- and MIDDLE-INCOME EDITION

Clinician Pro-Forma

Drug Treatments for Ovarian Cancer

39. Please indicate which of the following standard drugs used in the treatment of women with ovarian cancer are routinely available in your country, and who pays for them? Tick all that apply.

	Not routinely available	State pays	Patient or family pays	Insurance pays	NGO covers costs	Compassionate access programme (industry)
Carboplatin	☐	☐	☐	☐	☐	☐
Cisplatin	☐	☐	☐	☐	☐	☐
Paclitaxel (Taxol)	☐	☐	☐	☐	☐	☐
Pegylated Liposomal Doxorubicin Hydrochloride (Caelyx,Myocet,Doxil)	☐	☐	☐	☐	☐	☐
Gemcitabine	☐	☐	☐	☐	☐	☐
Trabectedin	☐	☐	☐	☐	☐	☐
Topotecan	☐	☐	☐	☐	☐	☐
Etoposide	☐	☐	☐	☐	☐	☐
Cyclophosphamide	☐	☐	☐	☐	☐	☐
Bleomycin	☐	☐	☐	☐	☐	☐

What are the key issues around access to these for ovarian cancer in your country? Please consider availability, affordability, reliability of supply, etc.

40. What about access to the following drugs. Are they routinely available in your country and who pays for them? Tick all that apply.

	Not routinely available	State pays	Patient or family pays	Insurance pays	NGO covers costs	Compassionate access programme (industry)
Tamoxifen	☐	☐	☐	☐	☐	☐
Anastrozole	☐	☐	☐	☐	☐	☐
Letrozole	☐	☐	☐	☐	☐	☐
Intraperitoneal chemotherapy	☐	☐	☐	☐	☐	☐
Hyperthermic Intraperitoneal chemotherapy	☐	☐	☐	☐	☐	☐
Bevacizumab	☐	☐	☐	☐	☐	☐
Olaparib	☐	☐	☐	☐	☐	☐
Rucaparib	☐	☐	☐	☐	☐	☐
Niraparib	☐	☐	☐	☐	☐	☐

What are the key issues around access to these for ovarian cancer in your country? Please consider availability, affordability, reliability of supply, etc.

41. Were you aware that all of the chemotherapy and hormone drugs outlined above are listed on the WHO's list of Essential Medicines?

- ◯ Yes
- ◯ No

Comments:

42. Is there a National Essential Medicines list in your country and if so, does it include all the epithelial ovarian cancer and non-epithelial ovarian cancer chemotherapies as well as all the hormone treatments listed?

- ◯ Yes there is a national list and some drugs are listed (please enter details in comments)
- ◯ Yes there is a national list and all drugs outlined are listed
- ◯ Yes there is a list, but none of the drugs are listed
- ◯ There is no National Essential Medicines List in my country
- ◯ I do not know

Comments:

43. Is there a significant group of women who are suspected of having ovarian cancer, or are diagnosed with ovarian cancer, who do not proceed with treatment in any form (surgery or chemotherapy)? Can you provide an estimate of the number of women who do not start treatment (as a proportion of all seen at your centre), and can you give any reasons for this?

Clinician Pro-Forma

Palliative Care and Pain Control

44. Are you and your teams able to provide sufficient pain relief for women with ovarian cancer, particularly for those with very advanced disease, or disease that is no longer treatable?

- Yes
- No
- Not sure
- Other (please specify)

45. Are there any barriers you face in terms of providing sufficient pain relief? Please give an outline.

46. Are there any opportunities to improve access to appropriate pain relief? If so, please give a brief outline.

47. Do you believe that enough is done to help women in the final stages of their disease? You might want to consider whether doctors tend to over-treat women, or families pressure doctors to over-treat women, or not? Or whether the role of palliative care is not embedded at any point in the disease pathway (or until it is too late)? Is this applicable to all cancers or are there specific issues in relation to ovarian cancer?

48. What more could be done for women with ovarian cancer approaching the end of their life in order to ease their burden?

49. What would your priority be to improve access to better pain relief and end-of-life care?

THE **EVERY WOMAN** STUDY™
LOW- and MIDDLE-INCOME EDITION

Clinician Pro-Forma

Multi-Disciplinary Working

50. Do you have multi-disciplinary team meetings or tumour boards to discuss care of women with ovarian cancer in your hospital?

- Yes
- No
- Not sure
- Other (please specify)

51. If you answered with a Yes to Q48, please list the roles of individuals who participate in these multi-disciplinary team meetings or tumour boards.

52. Do you participate in virtual or in person multi-disciplinary team meetings that involve professionals from other hospitals or countries?

- Yes, in-country
- Yes, international
- No
- Not sure

Comments

53. Do your patients have access to any form of re- or pre-habilitation? Tick all that apply.

- [ ] Access to a nutritionist pre-treatment
- [ ] Access to a nutritionist post treatment
- [ ] Access to physical rehabilitation post treatment
- [ ] Other
- [ ] No such access
- [ ] Comments

THE EVERY WOMAN STUDY™
LOW- and MIDDLE-INCOME EDITION

Clinician Pro-Forma

Family History and Genetic Testing

54. Do any centres/hospitals in your country offer genetic testing in relation to ovarian cancer?

- [ ] Yes, to identify family members at risk
- [ ] Yes, to determine treatment
- [ ] None of the above
- [ ] Other (please specify)

55. If you answered yes to either offering genetic testing to identify family members at risk or to determine treatment in Q51, please answer these two questions:

Who pays for the genetic testing?
Is it widely accessible?

56. Do you believe enough is done for women with a family history of ovarian cancer? Please consider genetic testing, risk reduction, acting on family history, surveillance and information.

57. With the advent of PARP inhibitors, do you think there is a need in your country to establish or develop existing genetic services?

58. Are there specific barriers to introducing or increasing testing capacity in your country?

THE EVERY WOMAN STUDY™
LOW- and MIDDLE-INCOME EDITION

THE EVERY WOMAN STUDY™ LOW- and MIDDLE-INCOME EDITION
WORLD OVARIAN CANCER COALITION IGCS
A JOINT INITIATIVE FOR WOMEN

Clinician Pro-Forma

Information and Support for Women

59. Prior to this study, has your hospital been able to provide any information on ovarian cancer (such as a leaflet) to women who are diagnosed?

- ○ Yes
- ○ No
- ○ Not sure
- ○ Other (please specify)

60. Does your hospital offer any of the following services for women with ovarian cancer?

- ☐ An ovarian cancer support group
- ☐ A gynaecological cancer support group
- ☐ A cancer support group (any type)
- ☐ None of the above
- ☐ Not sure
- ☐ Comments

61. Are you and your colleagues able to refer women for psychological support at your hospital? Please use the comment box to say whether or not this is typical in your country.

- ◯ Yes
- ◯ No
- ◯ I'm not sure

Comments

62. What do you believe are the greatest emotional challenges for women diagnosed with ovarian cancer in your region/country, regarding living with the disease?

63. Do you know of any examples of good practice with regards to information and support within your country, either directly relating to women with ovarian cancer, or people diagnosed with another form of cancer?

THE EVERY WOMAN STUDY™
LOW- and MIDDLE-INCOME EDITION

Clinician Pro-Forma

Workforce Issues

64. How would you describe the issues relating to the workforce in your centre and country that affect the care of women with ovarian cancer? Please think as widely as you can, such as workforce planning, training, infrastructure, specialisation, retention, access to resource and so on.

65. What would be your priorities for change in relation to these workforce issues?

66. Are these issues applicable in many disease areas or are some specific to the care of women with gynaecological cancer?

- [ ] Cancer in general
- [ ] Gynaecological cancers specifically
- [ ] Other (please specify)

67. Are there examples of good programmes to address some of these issues? Either in your hospital, area or country.

You might consider both government and non-governmental programmes that originate outside your country. If so please outline what works well in your country.

68. In your view, what are the biggest barriers to increasing and retaining clinicians, nurses and other essential staff who are vital for the care of women with ovarian cancer?

THE EVERY WOMAN STUDY™ LOW- and MIDDLE-INCOME EDITION

Clinician Pro-Forma

Collaboration and Opportunities

69. Do you routinely share information and learning with other doctors treating women with ovarian cancer, who are in other centres in your country? If so, what type of information do you share?

70. How often are you able to collaborate with other treatment centres in relation to the care of women with ovarian cancer? If this study is your first such collaboration, please let us know.

71. Do you have a national society for gynaecologic oncologists or a national oncology society? If yes, please give the name and indicate if you are a member.

- ○ Yes but I am not a member
- ○ Yes and I am a member
- ○ No
- ○ Not sure

Details (name of the society)

[ ]

72. Are there any regional, or national conferences and/or publications that you would be interested in approaching about your country's findings from the Every Woman Study?

- ○ Yes (please give details below)
- ○ No
- ○ Not sure

Conference details

[ ]

73. Are you a member of any of the following global or regional groups? Tick all that apply.

- ☐ African Organisation for Research and Training in Cancer (AORTIC)
- ☐ American Society of Clinical Oncology (ASCO)
- ☐ European Society of Gynecologic Oncology (ESGO)
- ☐ European Society of Medical Oncology (ESMO)
- ☐ International Gynecologic Cancer Society (IGCS)
- ☐ Pan-Arabian Society for Gynaecologic Oncology (PARSGO)
- ☐ World Ovarian Cancer Coalition (WOCC)
- ☐ Other (please specify)

[ ]

74. What opportunities might there be to increase collaboration and involvement with others working in ovarian cancer, either on a national, regional, or global basis?

[ ]
