# Supplementary material for: The every woman study™ low- and middle-income countries edition protocol: A multi-country observational study to assess opportunities and challenges to improving survival and quality of life for women with ovarian cancer
Source: PLoS One. 2024 May 29;19(5):e0298154. doi: 10.1371/journal.pone.0298154 (PMC11135759; doi:10.1371/journal.pone.0298154)
Supplement: S5 File — (PDF) [file pone.0298154.s006.pdf]

WHAT IS CANCER?

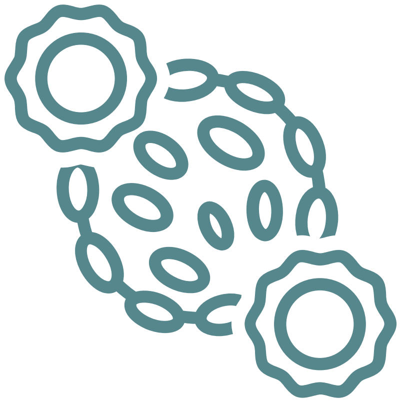

Our bodies are made up of millions of cells that are constantly being replaced so we can stay healthy and repair damage. When our body destroys an old cell, one of the remaining cells makes a copy of itself by diving in two, which replaces the old cell. While this happens millions and millions of times in our bodies without any problems, sometimes the cells keep on dividing, until eventually a lump called a tumour develops. If the tumour grows uncontrollably and goes into surrounding tissue or spreads to a different part of the body, it is described as "malignant" or cancerous.

OVARIAN CANCER IN XXXXXXXX

Every year approximately [enter Globocan annual diagnoses] women in [xxxxxx] are diagnosed with ovarian cancer. Doctors at this hospital are working with others in [xxxxxxx[and around the world to improve diagnosis and treatments for women with ovarian cancer, together with such groups as the International Gynecologic Cancer Society and the World Ovarian Cancer Coalition

Living with ovarian cancer can be challenging for women and their families. Please remember to let your doctor or nurse know of any issues affecting you. Other sources of support include:

NGO 1

NGO 2

CLINIC/HOSPITAL LOGO HERE

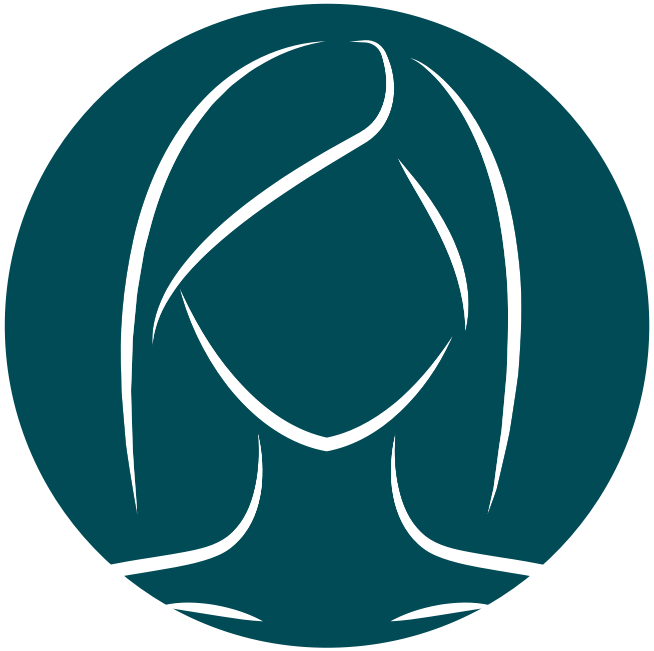

OVARIAN CANCER

THE **EVERY WOMAN** STUDY™
LOW- and MIDDLE-INCOME EDITION

WORLD OVARIAN CANCER COALITION
IGCS INTERNATIONAL GYNECOLOGIC CANCER SOCIETY
A JOINT INITIATIVE FOR WOMEN

This information has been jointly prepared by [xxxxxxxx hospital./clinic], the International Gynecologic Cancer Society and the World Ovarian Cancer Coalition. January 2022.

OVARIAN CANCER

The ovaries are two small organs, each around the size of an almond or your fingertip. They are located low in the tummy area, called the pelvis. They are part of a woman's reproductive organs, storing her supply of eggs. Each month an egg is released from the one of the ovaries and travels through the fallopian tubes into the womb. If the egg is fertilised, the woman will become pregnant.

Ovarian cancer is cancer that arises from the cells in and around the ovaries and fallopian tubes. There are different types of ovarian cancer, depending where they develop. Different forms of ovarian cancer will behave differently, and sometimes will need different types of treatment.

For all types of ovarian cancer an assessment is usually made to see if the cancer has spread, and if so, how far. This will affect what treatment you may be offered, and when. Most women are diagnosed once the cancer has already spread. It can spread to the surface of the abdomen (called the peritoneum), the bowels, the omentum (a layer of tissue that extends down from the stomach), the liver surface, the spleen, or the lungs. These tumours can often produce excess fluid in the belly, which can cause a lot of bloating and uncomfortable symptoms.

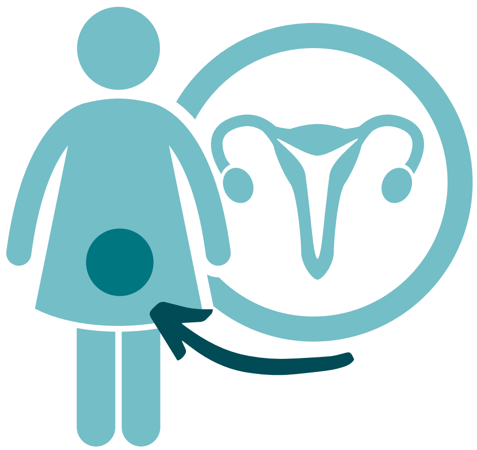

TREATMENT

Surgery to remove all or as much of the ovarian cancer as possible provides the best way of controlling the cancer, and sometimes curing it. Because this is a major operation, it may mean having to travel some distance to get the best surgery possible. If the cancer has begun to spread, then some form of chemotherapy drug treatment is usually offered as well. Sometimes the chemotherapy will be given first to shrink the tumours so the surgery will be easier. If the cancer comes back after treatment, more chemotherapy may be offered. Your doctor is the best person to talk to about treatments for your cancer.

You may or may not wish to know the details of your cancer, but they are important to your doctor as it will help them determine the best way to treat or control the disease.

WHO GETS OVARIAN CANCER?

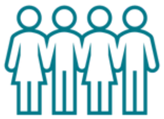

You cannot catch ovarian cancer from anyone else by close contact. Your risk of developing ovarian cancer increases as you get older, particularly after menopause (when your monthly bleeds stop and you can no longer have children). Younger women, though, can also be affected.

Most cases arise because of changes in your body as you age. Being overweight or smoking can increase the risk slightly for some less common types of ovarian cancer. Having several babies, breast feeding, or going on an oral contraceptive pill for several years can all reduce the risk.

Through no fault of anyone, for nearly one in five cases though, inherited genetic changes (known as mutations) can lead to several members of the same family being at risk of, or be affected by, cancer. This not only includes ovarian cancer, but also breast cancer (BRCA1/2), prostate cancer in men, and in some families, cancers of the bowel, womb, and pancreas. While men do not have ovaries, they can still inherit these altered genes, which means their children could be at risk of developing ovarian cancer.

If there are members of your close family - blood relatives such as parents, sisters, brothers, aunts/uncles, or grandparents - affected by any of these cancers, you should mention it to your doctor who is treating you. This may help your doctor identify others in your family who may be at risk and mean preventative action can be taken.
