# Supplementary material for: The every woman study™ low- and middle-income countries edition protocol: A multi-country observational study to assess opportunities and challenges to improving survival and quality of life for women with ovarian cancer
Source: PLoS One. 2024 May 29;19(5):e0298154. doi: 10.1371/journal.pone.0298154 (PMC11135759; doi:10.1371/journal.pone.0298154)
Supplement: S6 File — (PDF) [file pone.0298154.s007.pdf]

The Every Woman Study LMIC Edition – Eligibility Criteria and basic data

**Questions filled in by the administrator**	THIS INFORMATION SHOULD BE COMPLETED FOR ALL WOMEN AS INSTRUCTED, WHETHER OR NOT THE WOMAN CONSENTS TO TAKE PART.<br><br>This will allow us to analyse key factors about those who decline to participate.
**Unique Identifying Number**	______________
**EQ0**	In order to assess potential biases, it is important for us to know why you have selected this woman, with a confirmed diagnosis of ovarian cancer, for possible inclusion in the study. Please select the option that best describes the approach your team have taken in considering this woman:<br><br>We are reviewing ALL CASES in our records that HAVE been seen at this centre over the LAST 5 YEARS with a view to inviting eligible women<br><br>We are reviewing all women who have RECENTLY ATTENDED AN APPOINTMENT, WORKING BACKWARDS FROM THE MOST RECENT ATTENDEES IN A SEQUENTIAL MANNER, until we meet our required number of completions<br><br>We are reviewing all women DUE TO ATTEND an appointment in person or virtually in relation to their diagnosis and treatment, during the study period<br><br>Other. Please specify the other method used<br>………<br>(Other method)
EQ1	Is the patient normally resident in this country?<br><br>Yes, and was diagnosed here<br>Yes, but was diagnosed in another country (enter country of diagnosis)<br>No, but was diagnosed here<br>No, was diagnosed elsewhere and has travelled for treatment (enter country of diagnosis)<br><br>In what country was the patient diagnosed? ………………………………………
EQ2	When was the patient diagnosed with ovarian cancer?<br>Diagnosis Month…………………<br>Diagnosis Year………
EQ3	What age was the patient when she was diagnosed with ovarian cancer?<br><br>(Number)
EQ4	What type of ovarian cancer was the patient diagnosed with?

	Epithelial ovarian cancer (unspecified)<br>Epithelial (clear cell) ovarian cancer<br>Epithelial (high-grade serous) ovarian cancer<br>Epithelial (low-grade serous) ovarian cancer<br>Epithelial (endometrioid) ovarian cancer<br>Epithelial (mucinous) ovarian cancer<br>Germ cell ovarian cancer<br>Sex cord stromal ovarian cancer<br>Primary peritoneal ovarian cancer<br>Fallopian tube cancer<br>Granulosa tumour of the ovary<br>Borderline tumour<br>Teratoma of the ovary<br><br>If the patient has another type of ovarian cancer, or an additional type of ovarian cancer please enter it here.<br>…………………………………………………
EQ5	What form of staging of the patient's cancer has taken place?<br>Surgical or pathological staging<br>Clinical staging (based on physical exam, biopsy and imaging)<br>Clinical opinion only<br>I do not know
EQ6	What stage was the ovarian cancer diagnosed at? Select the answer which best describes the stage.<br><br>FIGO stage I (T1, N0, M0) Tumor limited to the ovaries (one or both)<br>FIGO stage IA (T1a, N0, M0)<br>FIGO stage IB (T1b, N0, M0)<br>FIGO stage IC (T1c, N0, M0)<br><br>FIGO stage II (T2, N0, M0) Tumor involves one or both ovaries with pelvic extension below pelvic brim<br>FIGO stage IIA (T2a, N0, M0)<br>FIGO stage IIB (T2b, N0, M0)<br><br>FIGO stage III Tumor involves one or both ovaries with microscopically confirmed peritoneal metastasis outside the pelvis and/or retroperitoneal lymph node involvement<br>FIGO stage IIIA (T1 or T2, N1, M0) or (T3a, N0 or N1, M0)<br>FIGO stage IIIB (T3b, N0 or N1, M0)<br>FIGO stage IIIC (T3c, N0 or N1, M0)<br><br>Figo Stage IV Distant metastasis including cytology-positive pleural effusion; liver or splenic parenchymal involvement; extra-abdominal organ involvement including inguinal lymph nodes; transmural intestinal involvement<br>FIGO Stage IVA (Any T, Any N, M1a)<br>FIGO Stage IVB (Any T Any N, M1b)

	I do not know
EQ7	Is the patient currently?<br><br>Undergoing or recovering from surgery<br>Undergoing treatment for newly diagnosed ovarian cancer<br>Undergoing treatment for recurrent ovarian cancer<br>In remission<br>Receiving palliative care<br>Choosing not to receive treatment<br>Other
EQ8	Has the patient's ovarian cancer ever returned?<br>Yes<br>No<br>It never went away<br><br>If the cancer returned, please enter the date it FIRST returned<br><br>Recurrence month........<br>Recurrence year............
**Inclusion Criteria**<br><br>**EQ9**	Does the patient meet the inclusion criteria?<br>Tick all that apply<br>• The patient can give informed consent for participation in the study.<br>• The patient is biologically female, aged between 18 and 99 years<br>• The patient has been diagnosed with ovarian, fallopian tube or primary peritoneal cancer within the previous five years (of the date of completing the survey)<br>• The patient has already been informed of their diagnosis of ovarian cancer at a previous appointment and understands the diagnosis<br><br>ALL ANSWERS NEED TO BE SELECTED IN ORDER TO PROCEED
**Exclusion criteria**<br><br>**EQ10**	Does the patient meet any of the following exclusion criteria?<br>Tick all that apply<br>• The patient is attending the hospital or clinic to receive their diagnosis of ovarian cancer.<br>• The patient is deemed too unwell to be able to cope with the demands of filling in the survey or responding to questions<br>• The patient is identified as having mental health concerns, learning difficulties, or medical conditions such as dementia, delirium, or psychosis to the extent that they would be unable to cope with the demands of filling in the survey or responding to questions<br>• The patient has already completed the survey on a previous visit to the hospital<br>• The patient does not meet any of the exclusion criteria.<br><br>IF ANY OF THE EXCLUSION CRITERIA ARE MET, THE PATIENT WILL BE EXCLUDED FROM THE STUDY AT THIS POINT. PLEASE RECORD THE RESULTS ON REDCap AND THE PAPER LOG.

	IF THE PATIENT IS ELIGIBLE TO PARTICIPATE PLEASE COMPLETE THE INFORMATION BELOW.

AQ2	Which tests were undertaken at any point, to decide if the patient had ovarian cancer? TICK ALL THAT APPLY<br><br>Clinical examination<br>CA125 blood test<br>Abdominal Ultrasound<br>Transvaginal ultrasound<br>MRI scan<br>CT scan<br>X-ray<br>Other<br>I do not know
AQ3	Has the patient ever had surgery to treat or control their ovarian cancer?<br><br>No (GO TO AQ5)<br><br>Yes, with complete tumour cytoreduction to no gross residual disease and all macroscopic residual disease removed. *This definition is in line with the ASCO Stratified Guidelines for Ovarian Cancer, Vanderpuye et al 2021*<br><br>Yes, but not with complete tumour cytoreduction to no gross residual disease and all macroscopic residual disease removed
AQ4	Has the patient ever had any of the following? Tick all that apply<br><br>Chemotherapy before first surgery (Neo-adjuvant chemotherapy)<br>A second operation because the first operation did not remove enough of the cancer<br>A second operation for a recurrence of ovarian cancer
AQ5	Has the patient ever had chemotherapy to treat or control their ovarian cancer?<br><br>Yes<br>No<br>(IF NO, Go to AQ7)
AQ6	Which chemotherapy drugs has the patient been treated with at any point since their diagnosis with ovarian cancer. Tick all that apply (list)<br><br>Carboplatin

	Cisplatin<br>Paclitaxel (Taxol)<br>Pegylated Liposomal Doxorubicin Hydrochloride (Caelyx,Myocet,Doxil)<br>Gemcitabine<br>Trabectedin<br>Topotecan<br>Etoposide<br>Cyclophosphamide<br>Bleomycin<br>Other
AQ7	Has the patient ever been tested to find any genetic mutations?<br><br>Yes, pre-diagnosis<br>Yes, post-diagnosis<br>No, they have not but it is available in this country<br>No, it is not available in this country<br>IF NO GO TO AQ9
AQ8 if answered yes to AQ7	Which genetic mutation was found?<br><br>BRCA1<br>BRCA2<br>Lynch Syndrome<br>Other<br>No genetic mutation was found
AQ9	Has the patient ever received one or more of the following hormone treatments? Tick all that apply<br>Tamoxifen<br>Anastrozole<br>Letrozole<br>These drugs are not available
AQ10	Are any of the following treatments available in your country, either routinely or occasionally? TICK ALL THAT APPLY<br><br>Intraperitoneal chemotherapy<br>Hyperthermic chemotherapy<br>Bevacizumab<br>Olaparib<br>Rucaparib<br>Niraparib<br>None of these treatments are available
AQ11	If any of the above treatments were selected, has this patient ever received any of them as part of her treatment in your country? TICK ALL THAT APPLY<br><br>Intraperitoneal chemotherapy

	Hyperthermic chemotherapy<br>Bevacizumab<br>Olaparib<br>Rucaparib<br>Niraparib<br>I don't know

That is all the clinical data that is required. Please upload the data to the Eligibility Criteria Instrument, mark it as complete and lock it. Please now speak in person, or by phone, with the woman to inform her of the Study, find out her preferred language, and determine how she should receive the consent form/survey (on paper, on a clinic device, by email or WhatsApp). Please note that consent should not be taken over the phone. Once you have this information please enter it in the Preferred Language Instrument
